# Four new hydroxyl fatty acids, gambaoic acids A-C and gambaoic B methyl ester, from Shrimp *Jeotgal*-derived *Bacillus* sp. SNB-066

**DOI:** 10.1038/s41429-026-00914-2

**Published:** 2026-04-03

**Authors:** Prima F. Hillman, Chaeyoung Lee, Mücahit Varlı, Rui Zhou, Sang-Ah Han, Minyi Yoo, Ji Young Lee, Jeong-Hyeon Kim, Songyi Lee, Hunmin Lee, Geum Jin Kim, Hyukjae Choi, Hangun Kim, Sang-Jip Nam

**Affiliations:** 1 Department of Chemistry, Faculty of Mathematics and Natural Sciences, Universitas Andalas, Kampus Limau Manis, Padang, 25163 Indonesia; 2 Department of Chemistry and Nanoscience, Ewha Womans University, Seoul, 03760 Republic of Korea; 3 College of Pharmacy, Sunchon National University, Sunchon, 57922 Republic of Korea; 4 Graduate School of Industrial Pharmaceutical Sciences, Ewha Womans University, Seoul, 03760 Republic of Korea; 5 Institute of Sustainable Earth and Environmental Dynamics (SEED), Pukyong National University, Busan, 48547 Republic of Korea; 6 Department of Chemistry, Pukyong National University, Busan, 48513 Republic of Korea; 7 Industry 4.0 Convergence Bionics Engineering, Pukyong National University, Busan, 48513 Republic of Korea; 8 College of Pharmacy, Yeungnam University, Gyeongsan, Gyeong-buk 38541 Republic of Korea; 9 Department of Pharmacology, School of Medicine, Dongguk University, Gyeongju, Gyeong-buk 38066 Republic of Korea; 10 Research Institute of Cell Culture, Yeungnam University, Gyeongsan, Gyeong-buk Republic of Korea; 11 Graduate Program in Innovative Biomaterials Convergence, Ewha Womans University, Seoul, 03760 Republic of Korea

**Keywords:** Natural products, Biochemistry

## Abstract

Four new hydroxyl fatty acids (HFAs), gambaoic acids A − C (**1** − **3**) and gambaoic B methyl ester (**4**), were isolated from *Bacillus* sp. SNB-066, a bacterium derived from shrimp *jeotgal*. The chemical structures of these HFAs were elucidated through extensive spectroscopic data analysis, including mass spectrometry (MS), ultraviolet (UV), and nuclear magnetic resonance (NMR) spectroscopy data. The relative configurations of compounds **2** and **3** were determined using quantum mechanics-based computational analysis with DP4^+^ statistical calculations. Further specific rotation analyses revealed the absolute configurations of both compounds **2** and **4** as 14 *R* and 15 *R*, while compound **3** was assigned as 14*S* and 15*S*. Antibacterial activity evaluated by the minimal inhibitory concentration (MIC) assay indicated that only compound **4** exhibited weak activity against the Gram-positive bacteria *B. subtilis* KCTC 1021 and *K. rhizophila* KCTC 1915, with MIC values of 64 μg/mL. Further, gambaoic acids B (**2**) and C (**3**) exhibited dose-dependent cytotoxicity toward Caco-2 cells, with gambaoic acid C (**3**) demonstrating a pronounced anti-invasive effect. These findings highlight the diverse bioactivities of the isolated hydroxyl fatty acids and reveal structural features underlying their antimicrobial and anticancer properties.

## Introduction

*Jeotgal* is a traditional Korean fermented seafood made by mixing fish and shellfish with salt and fermenting it for approximately one year. Shrimp *jeotgal* is a variation prepared by adding up to 30% sea salt to small sea shrimp (*Acetes japonicus*) [1–3]. During fermentation, the seafood undergoes autolytic and microbial proteolysis, resulting in the unique texture and rich flavor of shrimp *jeotgal* [4, 5]. Studies using both culture-dependent and culture-independent methods have revealed that endospore-forming bacilli, particularly *Bacillus* sp., are the dominant microorganisms present [4, 6–9]. These microorganisms exhibit strong proteolytic activity, which accelerates fermentation and contributes to the overall quality of the final product [10–12].

**Fig. 1:**
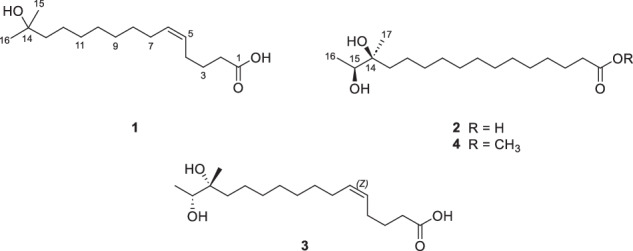
Chemical structure of compounds **1**−**4**

In addition to their role in fermentation, *Bacillus* spp. produce a variety of secondary metabolites with diverse biological activities, including antimicrobial, antialgal, and anticancer properties [13]. In this study, *Bacillus* sp. SNB-066 was isolated from shrimp *jeotgal*, and an intensive investigation of its chemical components has led to the discovery of four new hydroxyl fatty acids (HFAs), gambaoic acids A − C (**1** − **3**) and gambaoic B methyl ester (**4**) (Fig. 1). Here, this study reports the details of the isolation and structural characterization of new HFAs (**1** − **4**), as along with an evaluation of their biological activities.

## Materials and methods

### General experimental procedures

Optical rotations were acquired using a Kruss Optronic P-8000 polarimeter with a 5-cm cell. UV spectra were recorded with a 1260 Infinity Series UV visible spectrophotometer (Agilent Technologies, Santa Clara, CA, USA) using a 0.1 cm path length. IR spectra were obtained using a Varian Scimitar Series spectrometer. NMR spectra were acquired with an Agilent 400-MR DD2 NMR spectrometer (400 MHz for ^1^H and 100 MHz for ^13^C, Agilent Technologies, Santa Clara, CA, USA) at the Ewha Drug Development Research Core Center, using CD_3_OD as the solvent and its signal as an internal reference (*δ*_H_ 3.31/*δ*_C_ 49.1). Low resolution LC/MS data were collected using an Agilent Technologies 1260 quadrupole LC/MS system (Agilent Technologies, Santa Clara, CA, USA) and Waters Alliance Micromass ZQ LC-MS system (Waters Corp, Milford, MA, USA) at the National Research Facilities and Equipment Center (NanoBioEnergy Materials Center) at Ewha Womans University. Analyses were performed using a reversed-phase column (Phenomenex Luna C18 (2) 100 Å, 50 mm × 4.6 mm, 5 µm) at a flow rate 1.0 mL/min. Fractions were purified by semi-preparative HPLC, a Waters 616 quaternary HPLC pump and a Waters 996 photodiode array detector using reversed phase column (Phenomenex Luna C18 (2) 100 Å, 250 nm × 10 mm, 5 µm) at a flow rate of 2.0 mL/min. High-resolution FAB-MS spectra were acquired using a JEOL JMS-AX505WA mass spectrometer (JEOL Ltd., Tokyo, Japan) at Seoul National University. High-resolution ESI-TOF-MS spectra were acquired using Agilent 6230 TOF LC/MS equipped at Ewha Drug Development Research Core Center.

### Collection and phylogenetic analysis of strain SNB-066

For the isolation of halophilic or halotolerant bacteria, a type of salted shrimp (saeu-*jeotgal*) was purchased from a traditional market in Suwon, Korea. The sample was filtered through sterile gauze, and the filtrate was used for bacterial selection. The filtrate was diluted to an appropriate concentration for bacterial isolation using 1× PBS. The diluted sample was then spread on solid media and incubated at 27 °C for over 48 h. The media used for bacterial isolation included nutrient medium (Difco, USA) and marine medium (MBcell, Korea), both supplemented with 1.5% (w/v) agar. The final NaCl concentrations of the media were adjusted to 8% (w/v) and 15% (w/v). To ensure the selection of diverse colonies, approximately five colonies per sample were selected based on morphological characteristics such as size, color, and shape. These selected colonies were then purified using the same media employed during the isolation process.

The selected bacteria were phylogenetically identified through 16S rRNA sequence analysis. Strain SNB-066 was assigned as a member of the genus *Bacillus* sp. with 99.9% identity. The 16S rRNA gene sequence has been deposited in GenBank (accession number PV460738.1).

### Cultivation, extraction and isolation

*Bacillus* strain SNB-066 was cultured in 80 L of 2.5-L Ultra Yield Flasks each containing 1 L of the medium (10 g/L of soluble starch, 2 g/L of yeast, 4 g/L of peptone dissolved in 1 L seawater) at 25 °C with shaking at 120 rpm. After 7 days, the broth was extracted with EtOAc (80 L overall) to afford 1.64 g of EtOAc extract. The crude EtOAc extract was fractionated by C18 resin open column chromatography, eluting with a step gradient from 20 to 100% MeOH in distilled water (DW) (20/80, 30/70, 40/60, 50/50, 60/40, 70/30, 100/0, 100/0) to produce fractions F1-F8. The sixth fraction (210.1 mg) was purified by reversed-phase HPLC chromatography, eluting with 55% CH_3_CN in 1% TFA water to acquire gambaoic acid C (**3**, 3.9 mg, *t*_R_ = 16.1 min), gambaoic acid B (**2**, 2.9 mg, *t*_R_ = 19.6 min), and gambaoic acid A (**1**, 2.3 mg, *t*_R_ = 29.5 min).

A second fermentation batch under identical conditions yielded 2.9 g of extract. Following the same fractionation procedure, the sixth fraction (139.7 mg) was purified by reversed-phase HPLC using 30–100% gradient of CH_3_CN in water, affording gambaoic acid C (**3**, 4.5 mg, *t*_R_ = 25.6 min), gambaoic acid B (**2**, 6.0 mg, *t*_R_ = 28.0 min), and gambaoic B methyl ester (**4**, 3.5 mg, *t*_R_ = 35.0 min). The isolation and purification of compounds **1**–**4** were performed based on their physicochemical properties, including polarity and chromatographic behavior, following a physicochemical property-guided purification strategy.

*Gambaoic acid A (****1****)*: pale yellow oil; UV (MeOH) λ_max_ 201 nm; IR (KBr) *ν*_max_ 3419, 2928, 2853, 2360, 1714 cm^-1^; ^1^H, ^13^C and 2D NMR (400 MHz, CD_3_OD), see Table S1; HR-FAB-MS data [M + H-H_2_O]^+^ at *m/z* 253.2162 (calcd for C_16_H_29_O_2_, 253.2168).

*Gambaoic acid B (****2****)*: dark yellow oil; [α]_D_^25^ – 64.00 (*c* 1.0, MeOH); UV (MeOH) λ_max_ 201 nm; IR (KBr) *ν*_max_ 3445, 2924, 2852, 1635, 1457, 1375, 1188, 501 cm^-1^; ^1^H, ^13^C and 2D NMR (400 MHz, CD_3_OD), see Table S1; HR-FAB-MS data [M + H-H_2_O]^+^ at *m/z* 285.2431(calcd for C_17_H_33_O_3_, 285.2430).

*Gambaoic acid C (****3****)*: dark yellow oil; [α]_D_^25^ + 8.57 (*c* 0.14, MeOH); UV (MeOH) λ_max_ 201 nm; IR (KBr) *ν*_max_ 3445, 2929, 2854, 1705, 1645, 1456, 1375, 1187, 517 cm^-1^; ^1^H, ^13^C and 2D NMR (400 MHz, CD_3_OD), see Table S1; HR-ESI-TOF-MS data [M–H]^–^ at *m/z* 299.2226 (calcd for C_17_H_31_O_4_, 299.2222).

*Gambaoic B methyl ester (****4****)*: dark yellow oil; [α]_D_^25^ – 29.56 (*c* 0.1, MeOH); UV (MeOH) λ_max_ 201 nm; IR (KBr) *ν*_max_ 3448, 1635, 528 cm^-1^; ^1^H, ^13^C and 2D NMR (400 MHz, CD_3_OD), see Table S1; HR-ESI-TOF-MS data [M+Na]^+^ at *m/z* 339.2507 (calcd for C_18_H_36_O_4_Na, 339.2511).

### Conformational analysis, computational calculation for NMR and specific rotation

Conformational analyses for **2a, 2b, 3a** and **3b** were performed by using Molecular Mechanics Force Field (MMFF) with Spartan 18 package (Wavefunction, Irvine, CA, USA). Several conformers within 5% relative energy Boltzmann distribution window were selected and subjected to further computational calculation for geometric optimization. The selected conformers were applied to optimization of geometries at B3LYP/6-31 G(d,p) level of density functional theory (DFT) in PCM model of MeOH by Gaussian 16 (Gaussian Inc., Wallingford, CT, USA) [14]. The geometric optimized conformers were performed computational calculation with B3LYP/6-31 + G(d,p) level of GIAO method in PCM model of MeOH for NMR calculation by Gaussian 16. DP4+ probability analyses were performed using the Excel spreadsheet provided from reference [15]. The selected conformers of conformational analysis by Spartan 18 were further subjected to geometric optimization and specific rotation calculation by Gaussian 16 in PCM model of methanol with B3LYP/6-311 + G(2 d,p) and B3LYP/6-311 + + G(2 d,2p) basis sets respectively. The overall calculated specific rotations were generated according to the distributions derived from relative Gibbs free energy.

### Antibacterial activity

Bacterial cultures (three Gram-positive bacteria *Bacillus subtilis* KCTC1021, *Staphylococcus aureus* KCTC1927, and *Kocuria. rhizophila* KCTC1915 and three Gram-negative bacteria *Escherichia coli* KCTC2441, *Klebsiella pneumonia* KCTC2690, and *Salmonella typhimurium* KCTC2515) were grown overnight in Mueller Hinton broth (MHB) and adjusted to McFarland standard 0.5 (1.5 × 10^8^ cfu/mL). The test compounds, as well as the positive controls (vancomycin and ampicillin), were dissolved in DMSO at a concentration of 256 μg/mL and DMSO used as the negative control. Then, 100 μL of each compound was added to the first well of a sterile 96-well plate that already contained 50 μL of MHB (Mueller-Hinton Broth). Subsequently, the compounds were serially diluted to obtain final concentrations of 128, 64, 32, 16, 8, 4, 2, 1, 0.5, 0.25 and 0.125 μg/mL. Next, 50 μL of appropriately adjusted bacterial cultures were added to each well. The total concentration of inoculum bacteria was equivalent to 5.0 × 10^5^ cfu/mL. The 96-well plates were then incubated at 37 °C for 18–24 h. To determine the MIC value, growth suppression was evaluated through visual observation to check whether microbial growth and inhibition were observed, and 96-well used in the experiment was visually examined against light. Each sample was tested in triplicate, and the experiment was repeated three times [4].

### Cell culture

Human cancer cells Caco-2 cells (*Homo sapiens*; CVCL_0025; #HTB-37) and human gastric cancer AGS cells (*Homo sapiens*; CVCL_0139; #CRL-1739) were cultured in Dulbecco’s Modified Eagle’s Medium (DMEM; #CM001-050, GenDepot, Katy, TX, USA) or Roswell Park Memorial Institute (RPMI; #CM058-050) supplemented with 10% fetal bovine serum (FBS; #F0600-050, GenDepot) and 1% penicillin/streptomycin (#CA005-010, GenDepot). Cells were maintained at 37 °C in a humidified incubator with 5% CO₂.

### Cell viability assay

Cell viability was assessed using the MTT assay. Caco-2 cells were seeded into 96-well plates at a density of 3 × 10³ cells per well in 100 µL of complete growth medium (DMEM supplemented with 10% fetal bovine serum and 1% penicillin-streptomycin) and incubated overnight at 37 °C in a humidified atmosphere with 5% CO₂. The following day, cells were treated with test compounds at various concentrations, while the control group received the corresponding volume of DMSO control. After 48 h of treatment, 10 µL of MTT solution (5 mg/mL in PBS) was added to each well and incubated for 3 h at 37 °C. The resulting formazan crystals were dissolved by adding 100 µL of DMSO per well, and the absorbance was measured at 570 nm using a microplate reader [16].

### Transwell invasion assay

Cell invasion was assessed using Transwell inserts (Corning, Corning, NY, USA) equipped with polycarbonate membranes (8 μm pore size), which were pre-coated with 1% (w/v) gelatin. Caco-2 cells were suspended in medium containing 0.2% (v/v) bovine serum albumin (BSA) and incubated with either the test compound or DMSO as a control for 24 h. The lower compartments of the chambers were filled with 600 µL of DMEM medium supplemented with 0.2% (v/v) BSA and 10 µg/mL fibronectin to serve as a chemoattractant. Following 24 h of incubation, cells that had migrated through the membrane were fixed and stained using the Diff-Quik staining kit. Images of the invaded cells were captured under a microscope, and cell quantification was conducted using IMT iSolution software version 21.1 (IMT i-Solution Inc., Northampton, NJ, USA) [17].

### Statistical analysis

Statistical analyses were performed using Sigma Plot 12.5 software (Systat Software Inc., Chicago, IL, USA). The statistical significance between two groups was compared using the Student’s *t*-test and *p* values  <  0.05 were considered statistically significant.

## Results and discussion

Compound **1** was obtained as a pale-yellow oil, and its molecular formula was deduced as C_16_H_30_O_3_, based on analysis of HR-FAB-MS data [M + H-H_2_O]^+^ at *m/z* 253.2162 (calcd for C_16_H_29_O_2_, 253.2168, Fig. S6). The ^1^H NMR spectrum of **1** revealed two olefinic protons at *δ*_H_ 5.41 (1H, ttd, H-6) and 5.35 (1H, ttd, H-5); ten methylene groups at *δ*_H_ 2.28 (2H, td, *J* = 7.4, 4.6 Hz, H-2), 2.08 (2H, m, H-4), 2.05 (2H, m, H-7), 1.65 (2H, m, H-3), 1.43 (2H, m, H-13), and 1.30–1.39 (10H, m, H-8–12); and two methyl groups at *δ*_H_ 1.17 (6H, s, H-15, H-16). The ^13^C NMR and HSQC spectra displayed 16 carbon signals, comprising of one carboxylic carbon at *δ*_C_ 177.8 (C-1); two olefinic carbons at *δ*_C_ 131.9 (C-6) and 129.7 (C-5); one oxygenated quaternary carbon at *δ*_C_ 71.5 (C-14); ten methylene carbons at *δ*_C_ 44.9 (C-13), 34.4 (C-2), 31.4 (C-12), 30.8 (C-10), 30.6 (C-9), 30.3 (C-8), 28.1 (C-7), 27.5 (C-4), 26.1 (C-3), and 25.4 (C-11); and two methyl carbons at *δ*_C_ 29.1 (C-15, C-16) (Fig. S1and S2).

**Fig. 2:**
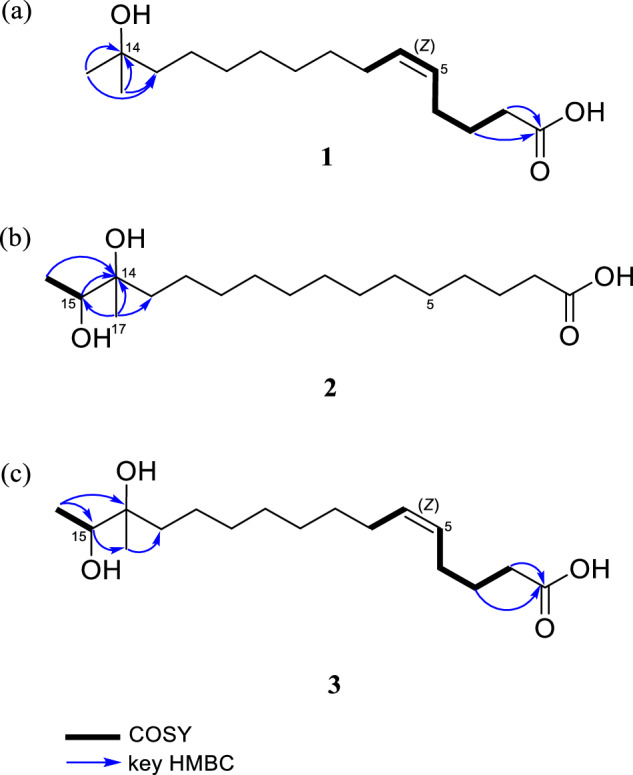
COSY and key HMBC correlations of compound **a** **1**, **b** **2**, and **c** **3**

The structure of compound **1** was elucidated using 2D NMR spectroscopic data analysis (Fig. 2a, Fig. S3–S5). The COSY crosspeaks H-2/H-3/H-4/H-5/H-6/H-7 together with HMBC correlations from H-2 to C-1, C-3, C-4; from H-3 to C-1, C-2, C-4, C-5; and from H-4 to C-2, C-3, C-5, C-6, confirmed the presence of olefinic carbons between C-5 and C-6. Furthermore, HMBC correlations from the geminal methyl singlets H-15 and H-16 to carbons C-13 and C-14 established the connectivity of the C-13/C-14/C-15/C-16 fragment. The attachment of a hydroxy group at C-14 was confirmed by its chemical shift (*δ*_C_ 71.5) and the molecular formula of **1**. The geometry of the olefinic protons was assigned as *Z* based on the observed coupling constant (^3^*J*_HH_ = 11.3 Hz), consistent with typical values for cis-configured double bonds. Thus, the structure of **1** was assigned as (5*Z*)-14-methyl-14-hydroxypentadec-5-enoic acid (gambaoic acid A).

Gambaoic acid B (**2**) was isolated as a dark yellow oil, and its molecular formula was deduced as C_17_H_34_O_4_ by HR-FAB-MS data [M + H-H_2_O]^+^ at *m/z* 285.2431(calcd for C_17_H_33_O_3_, 285.2430, Fig. S12). The ^1^H NMR spectrum of compound **2** showed one oxygenated methine at *δ*_H_ 3.56 (1H, q, *J* = 6.5 Hz, H-15); twelve methylene groups at *δ*_H_ 2.28 (2H, t, *J* = 7.4 Hz, H-2), 1.60 (2H, m, H-3), 1.40 (2H, m, H-13), and 1.31–1.39 (18H, m, H-4–12); and two methyl groups at *δ*_H_ 1.12 (3H, d, *J* = 6.5 Hz, H-16) and 1.08 (3H, s, H-17). The ^13^C NMR and HSQC spectroscopic data displayed 17 carbons signals, comprising one carboxylic carbon at *δ*_C_ 177.7 (C-1); one oxygenated quaternary carbon at *δ*_C_ 75.7 (C-14); one oxygenated methine at *δ*_C_ 74.1 (C-15); twelve methylene carbons at *δ*_C_ 39.2 (C-13), 35.0 (C-2), 31.6 (C-11), 30.8 (C-10), 30.7 (C-7, C-8, C-9), 30.6 (C-6), 30.4 (C-5), 30.2 (C-4), 26.1 (C-3), and 24.4 (C-12); and two methyl carbons at *δ*_C_ 21.6 (C-17) and 17.6 (C-16) (Fig. S7 and S8). The COSY correlation between H-15 and H-16 was observed. Moreover, HMBC correlations from H-15 to C-13, C-14, C-16, C-17; from H-16 to C-14, C-15; and from H-17 to C-13, C-14, C-15 confirmed the assignment of the dimethyl-diol moiety (Fig. 2b, Fig. S9–S11). Therefore, the gross structure of **2** was determined as 14-methyl-14,15-dihydroxyhexadecanoic acid, designated gambaoic acid B.

Compound **3** was obtained as a dark yellow oil with its molecular formula determined as C_17_H_32_O_4_, based on HR-ESI-TOF-MS data, showing an ion peak at [M–H]^–^ *m/z* 299.2226 (calcd for C_17_H_31_O_4_, 299.2222, Fig. S18). The ^1^H and ^13^C NMR spectroscopic data of **3** closely resembled those of compound **1** (Fig. S13–S17), with notable differences in the oxygenated methine group H-15 (*δ*_H_ 3.56, *δ*_C_ 74.1) and the methyl doublet H-16 (*δ*_H_ 1.12, *δ*_C_ 17.6). Furthermore, the structural assignment was supported by COSY correlations between H-15 (*δ*_H_ 3.56) and H-16 (*δ*_H_ 1.12), as well as HMBC correlations from H-15 to C-17 (*δ*_C_ 21.6); from H-16 to C-14 (*δ*_C_ 75.7), C-15; and from H-17 to C-13 (*δ*_C_ 39.2). The *Z* geometry of the double bond was established based on the coupling constant value (^3^*J*_HH_ = 11.3 Hz). Consequently, compound **3** was identified as (5*Z*)-14,15-dihydroxyhexadec-5-enoic acid (gambaoic acid C) (Fig. 2c).

Gambaoic B methyl ester (**4**) was isolated as a dark yellow oil, with its molecular formula determined as C_18_H_36_O_4_, based on HR-ESI-TOF-MS data, showing [M+Na]^+^ at *m/z* 339.2507 (calcd for C_18_H_36_O_4_Na 339.2511, Fig. S24). The ^1^H NMR spectrum of **4** was nearly identical to that of **2** except for the appearance of a methoxy group at *δ*_H_ 3.65 (3H, s, H-18) (Fig. S19–S23). This group was attached to the carbonyl group at C-1, based on the observation of HMBC correlation from H-18 to C-1. Thus, compound **4** was identified as the methyl ester derivative of gambaoic acid B (**2**).

**Fig. 3:**
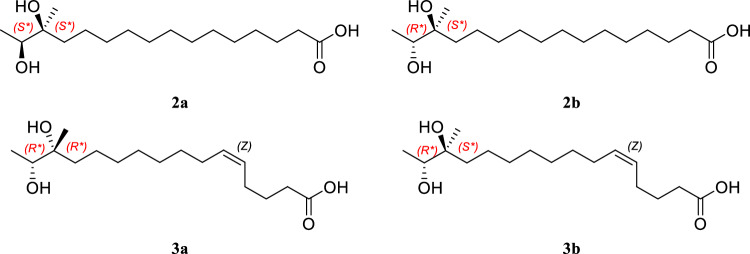
Structures of diastereomers of compounds **2** and **3**

To determine the relative configurations of compounds **2** and **3**, quantum mechanics-based computational analyses were carried out using DP4+ statistical calculations [14]. The conformers of two sets of diastereomers (**2a** and **2b**; **3a** and **3b**) were investigated using Spartan 18 software (Fig. 3). Subsequently, the structures of the low-energy conformers (with relative energies below 5 kJ/mol) were further optimized using Gaussian 16 software [14]. These energy-minimized conformers analyzed by the Gauge-Independent Atomic Orbital (GIAO) method, and shielding tensor values were calculated, considering the Boltzmann distribution of each conformer [18, 19].

By comparing the experimental chemical shift values with the calculated shielding tensor values in DP4+ analysis, our computational analysis indicated that compound **2** corresponded to diastereomer **2a** with a 100.00% probability across all data. For compound **3**, the DP4+ results based on the experimental 1D NMR data indicated that diastereomer **3a** was the most likely configuration, with a 99.99% probability.

The absolute configurations of compounds **2** − **4** were then assigned by comparing the calculated and observed specific rotations. The observed and calculated specific rotation values for **2** and **4** (calculated [α]_D_ + 8.05 for **2a** with 14*S*, 15*S*; observed [α]_D_^25^ − 64.00 for **2**, [α]_D_^25^ − 29.56 for **4**) supported the 14 *R* and 15 *R* configurations. Similarly, the absolute configurations of compound **3** (calculated [α]_D_ − 18.74 for **3a** with 14 *R*, 15 *R*; observed [α]_D_^25^ + 8.57) were assigned as 14*S* and 15*S*.

To explore the biological activities of the isolated compounds, **1** − **4** were tested for antibacterial activity with the minimal inhibitory concentration (MIC) assay. Compound **4** exhibited weak antibacterial activities against Gram-positive bacteria *B. subtilis* KCTC 1021 and *K. rhizophila* KCTC 1915 with MIC values of 64 μg/mL, respectively. In contrast, compounds **1** − **3** did not show any antibacterial activities against tested pathogens (Table 1).

**Table 1:** MIC results of compounds 1−4

Compound	MIC (μg/mL)^a^					
	Gram (+) bacteria			Gram (–) bacteria		
	*B. subtilis* KCTC1021	*K. rhizophila* KCTC1915	*S. aureus* KCTC1927	*E. coli* KCTC2441	*S. typhimurium* KCTC2515	*K. pneumonia* KCTC2690
**1**	>128	>128	>128	>128	>128	>128
**2**	>128	>128	>128	>128	>128	>128
**3**	>128	>128	>128	>128	>128	>128
**4**	64	64	>128	>128	>128	>128
Ampicillin	0.25	0.25	0.25	4	2	>128
Vancomycin	0.25	0.5	0.5	>128	>128	>128
DMSA	>128	>128	>128	>128	>128	>128

^a^Each sample was tested in triplicate and repeated three times

**Fig. 4:**
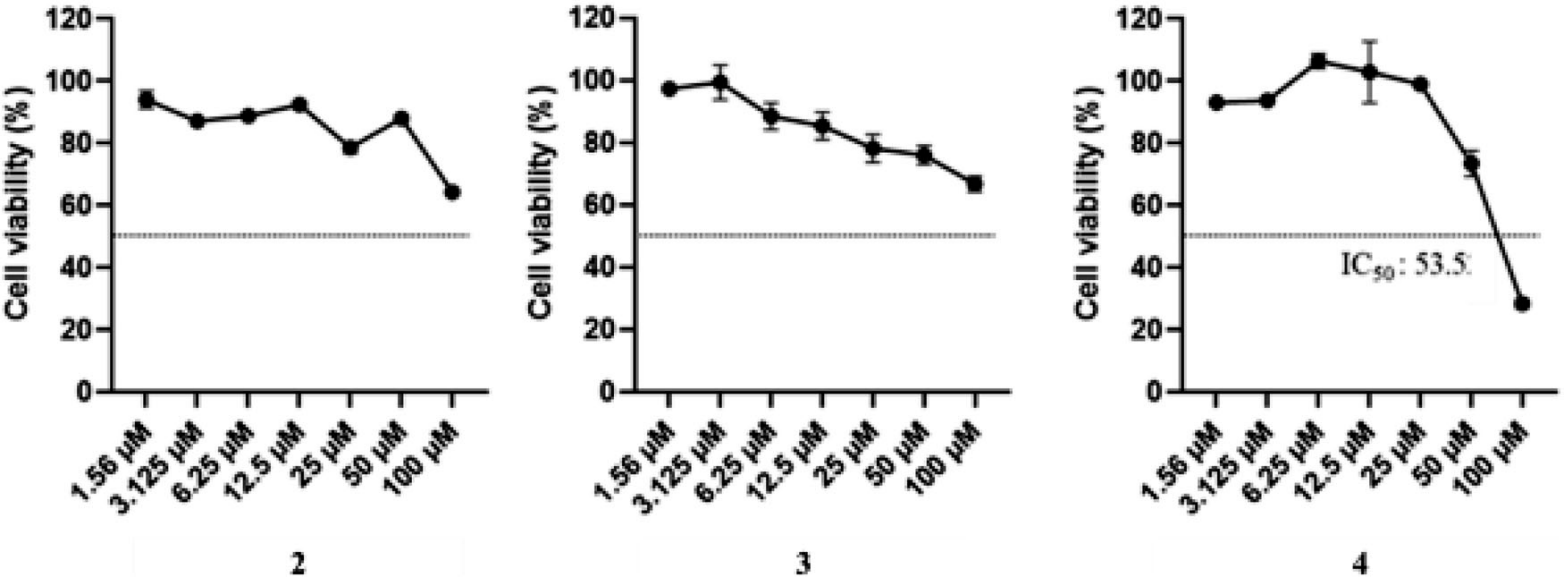
Effects of compounds **2**−**4** on Caco-2 cell viability. Caco-2 cells were treated with hydroxyl fatty acids at concentrations of 1.56–100 µM for 48 h, and viability was assessed using the MTT assay. DMSO-treated cells served as vehicle control. *p < 0.05; **p < 0.01; ***p < 0.001 vs. DMSO; *n*=3. Statistical significance was determined using a one-tailed Student's *t*-test

The cytotoxicity of compounds **2** − **4** was further evaluated in Caco-2 cells using the MTT assay after 48 h of treatment (Fig. 4). In the DMSO-treated control group, cell viability remained stable at 100% across all concentrations. Gambaoic acid B (**2**) induced a dose-dependent reduction in Caco-2 cell viability, with the strongest effect at 100 µM ( ~ 64%), followed by moderate decreases at 50 µM ( ~ 75–80%) and 25 µM ( ~ 80–85%). Gambaoic acid C (**3**) also reduced cell viability in a concentration-dependent manner but to a lesser extent, maintaining ~66% viability at 100 µM and >80% viability at intermediate concentrations, indicating a milder cytotoxic profile. In contrast, gambaoic B methyl ester (**4**) exhibited markedly stronger cytotoxicity, reducing viability to ~28% at 100 µM and to ~73% at 50 µM with an IC₅₀ value of 53.5 µM. Collectively, these results indicate that the methyl ester derivative (**4**) is substantially more potent than the corresponding acids (**2** and **3**) in reducing Caco-2 cell viability.

**Fig. 5:**
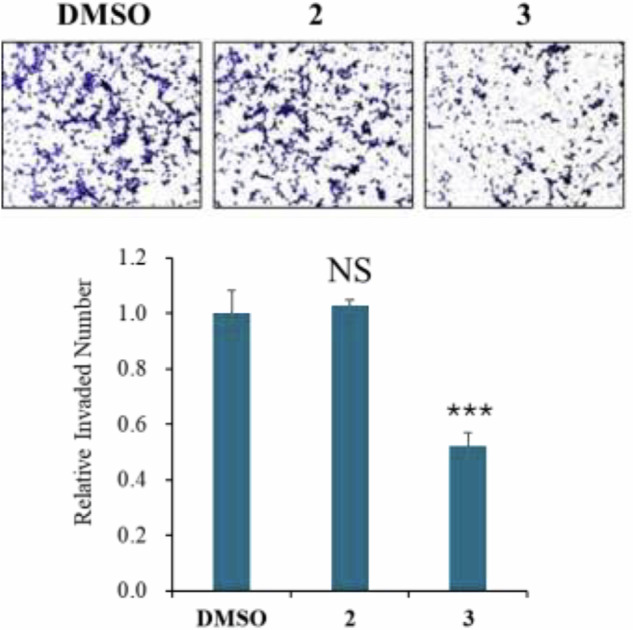
Effects of compounds **2** and **3** on the invasive capacity of Caco-2 cells. Caco-2 cells were treated with 10 µM of **2** or **3**, and cell invasion was assessed using a gelatin-coated Transwell invasion assay. Representative images of invaded cells stained with Diff-Quik are shown. The number of invaded cells was quantified and normalized to the DMSO control group. ***p < 0.001 vs. DMSO; NS, not significant; *n*=3. Statistical significance was determined using a one-tailed Student's *t*-test

Cancer cell invasion is a critical step in tumor progression, enabling cancer cells to penetrate surrounding tissues and spread to distant sites. This process drives metastasis, a leading cause of cancer mortality. Enhanced invasive ability is associated with poor prognosis in many cancers, including colorectal cancer [17–19]. Therefore, we examined the effects of compounds **2** and **3** on the invasive capacity in Caco-2 cells due to their poor cytotoxic effects (Fig. 5). Treatment with gambaoic acid C (**3**) significantly reduced Caco-2 cell invasion, as demonstrated by the Transwell invasion assay. After 24 h of exposure to 10 µM of compound **3**, the number of invaded cells was markedly lower than in the DMSO control group (***p < 0.001), indicating a strong anti-invasive effect. In contrast, gambaoic acid B (**2**) at the same concentration showed no significant effect, with invaded cell numbers comparable to the control group (NS). These findings suggest that gambaoic acid C (**3**) possessed a distinct inhibitory effect on colorectal cancer cell invasion, whereas gambaoic acid B (**2**) lacks this activity. The observed difference in bioactivity between the two analogs highlights the importance of structural or functional variations and warrants further investigation into their mechanisms of action.

Hydroxy fatty acids (HFAs), characterized by the presence of hydroxy groups on the aliphatic carbon chain, may occur in saturated or unsaturated forms and occasionally exhibit chain branching [20]. The isolation of gambaoic acids A − C (**1** − **3**), and gambaoic B methyl ester (**4**), from *Bacillus* sp. SNB-066 adds to the repertoire of bacterial-derived metabolites. Compounds with similar structural features have previously been reported from hypersaline cyanobacterial mats and from the fruit of *Capsicum annuum* L [21, 22]. In addition, hydroxylated unsaturated fatty acids, ieodomycins A–D, isolated from a marine-derived *Bacillus* sp. exhibited antimicrobial activity [23]. Collectively, these findings place compounds **1**–**4** within an expanding class of hydroxylated bacterial fatty acid metabolites. The structural diversity of microbial fatty acids, particularly from marine *Bacillus* strains, continues to broaden our understanding of microbial biosynthetic potential. Further study of their biosynthetic pathways and biological functions may provide valuable insights for natural product-based drug discovery, particularly in identifying novel pharmacophores from marine microbial metabolites.

The limited availability of isolated material restricted additional biological evaluation in the present study. Future studies involving large-scale fermentation or chemical synthesis will enable more comprehensive biological characterization.

## Supplementary information

Four new hydroxyl fatty acids, gambaoic acids A-C and gambaoic B methyl ester, from Shrimp Jeotgal-derived Bacillus sp. SNB-066.
