# Correction: Four new hydroxyl fatty acids, gambaoic acids A-C and gambaoic B methyl ester, from Shrimp *Jeotgal*-derived *Bacillus* sp. SNB-066

**DOI:** 10.1038/s41429-026-00922-2

**Published:** 2026-04-17

**Authors:** Prima F. Hillman, Chaeyoung Lee, Mücahit Varlı, Rui Zhou, Sang-Ah Han, Minyi Yoo, Ji Young Lee, Jeong-Hyeon Kim, Songyi Lee, Hunmin Lee, Geum Jin Kim, Hyukjae Choi, Hangun Kim, Sang-Jip Nam

**Affiliations:** 1 Department of Chemistry, Faculty of Mathematics and Natural Sciences, Universitas Andalas, Padang, Indonesia; 2 Department of Chemistry and Nanoscience, Ewha Womans University, Seoul, Republic of Korea; 3 College of Pharmacy, Sunchon National University, Sunchon, Republic of Korea; 4 Graduate School of Industrial Pharmaceutical Sciences, Ewha Womans University, Seoul, Republic of Korea; 5 Institute of Sustainable Earth and Environmental Dynamics (SEED), Pukyong National University, Busan, Republic of Korea; 6 Department of Chemistry, Pukyong National University, Busan, Republic of Korea; 7 Industry 4.0 Convergence Bionics Engineering, Pukyong National University, Busan, Republic of Korea; 8 College of Pharmacy, Yeungnam University, Gyeongsan, Gyeong-buk Republic of Korea; 9 Department of Pharmacology, School of Medicine, Dongguk University, Gyeongju, Gyeong-buk Republic of Korea; 10 Research Institute of Cell Culture, Yeungnam University, Gyeongsan, Gyeong-buk Republic of Korea; 11 Graduate Program in Innovative Biomaterials Convergence, Ewha Womans University, Seoul, Republic of Korea

**Keywords:** Natural products, Biochemistry

Correction to: *The Journal of Antibiotics*

10.1038/s41429-026-00914-2; published online 03 April 2026

In this article, the author’s name, Mücahit Varlı, was incorrectly written as Mücahit Varli. The original article has been corrected.

