# Supplementary material for: Four new hydroxyl fatty acids, gambaoic acids A-C and gambaoic B methyl ester, from Shrimp Jeotgal-derived Bacillus sp. SNB-066
Source: J Antibiot (Tokyo). 2026 Apr 3;79(6):359–66. doi: 10.1038/s41429-026-00914-2 (PMC13212154; doi:10.1038/s41429-026-00914-2)
Supplement: Supplementary file 1 — Four new hydroxyl fatty acids, gambaoic acids A-C and gambaoic B methyl ester, from Shrimp Jeotgal-derived Bacillus sp. SNB-066. [file 41429_2026_914_MOESM1_ESM.docx]

**Supporting Information**

**Four new hydroxyl fatty acids, gambaoic acids A-C and gambaoic B methyl ester, from Shrimp Jeotgal-derived *Bacillus* sp. SNB-066**

**Prima F. Hillman^1,†^, Chaeyoung Lee^2,†^, Mücahit Varlı^3^, Rui Zhou^3^, Sang-Ah Han^4^, Minyi Yoo^2^, Ji Young Lee^5^, Jeong-Hyeon Kim^2^, Songyi Lee^6,7^, Hunmin Lee^8^, Geum Jin Kim^9^, Hyukjae Choi^8,10^ , Hangun Kim^3,*^ and Sang-Jip Nam^2,11,*^**

^1^Department of Chemistry, Faculty of Mathematics and Natural Sciences, Universitas Andalas, Kampus Limau Manis, Padang 25163, Indonesia

^2^Department of Chemistry and Nanoscience, Ewha Womans University, Seoul 03760, Republic of Korea

^3^College of Pharmacy, Sunchon National University, Sunchon 57922, Republic of Korea

^4^Graduate School of Industrial Pharmaceutical Sciences, Ewha Womans University, Seoul 03760, Republic of Korea

^5^Institute of Sustainable Earth and Environmental Dynamics (SEED), Pukyong National University, Busan 48547, Republic of Korea

^6^Department of Chemistry, Pukyong National University, Busan 48513, Republic of Korea.

^7^Industry 4.0 Convergence Bionics Engineering, Pukyong National University, Busan 48513, Republic of Korea

^8^College of Pharmacy, Yeungnam University, Gyeongsan, Gyeong-buk 38541, Republic of Korea

^9^Department of Pharmacology, School of Medicine, Dongguk University, Gyeongju, Gyeong-buk 38066, Republic of Korea

^10^Research Institute of Cell Culture, Yeungnam University, Gyeongsan, Gyeong‐buk, Republic of Korea

^11^Graduate Program in Innovative Biomaterials Convergence, Ewha Womans University, Seoul 03760, Republic of Korea

**^†^** These authors contributed equally to this work as first authors

**Corresponding Authors:** [sjnam@ewha.ac.kr](mailto:sjnam@ewha.ac.kr); hangunkim@sunchon.ac.kr

**Table of Contents**

1. **NMR spectra and HRMS data**

**Figure S1.** ^1^H NMR spectrum of gambaoic acid A (**1**) in CD_3_OD 4

**Figure S2.** ^13^C NMR spectrum of gambaoic acid A (**1**) in CD_3_OD 5

**Figure S3.** ^1^H-^1^H COSY gambaoic acid A (**1**) in CD_3_OD 6

**Figure S4.** HSQC NMR spectrum of gambaoic acid A (**1**) in CD_3_OD 7

**Figure S5.** HMBC NMR spectrum of gambaoic acid A (**1**) in CD_3_OD 8

**Figure S6.** HRMS spectrum of gambaoic acid A (**1**) 9

**Figure S7.** ^1^H NMR spectrum of gambaoic acid B (**2**) in CD_3_OD 10

**Figure S8.** ^13^C NMR spectrum of gambaoic acid B (**2**) in CD_3_OD 11

**Figure S9.** ^1^H-^1^H COSY gambaoic acid B (**2**) in CD_3_OD 12

**Figure S10.** HSQC NMR spectrum of gambaoic acid B (**2**) in CD_3_OD 13

**Figure S11.** HMBC NMR spectrum of gambaoic acid B (**2**) in CD_3_OD 14

**Figure S12.** HRMS spectrum of gambaoic acid B (**2**) 15

**Figure S13.** ^1^H NMR spectrum of gambaoic acid C (**3**) in CD_3_OD 16

**Figure S14.** ^13^C NMR spectrum of gambaoic acid C (**3**) in CD_3_OD 17

**Figure S15.** ^1^H-^1^H COSY gambaoic acid C (**3**) in CD_3_OD 18

**Figure S16.** HSQC NMR spectrum of gambaoic acid C (**3**) in CD_3_OD 19

**Figure S17.** HMBC NMR spectrum of gambaoic acid C (**3**) in CD_3_OD 20

**Figure S18.** HRMS spectrum of gambaoic acid C (**3**) 21

**Figure S19.** ^1^H NMR spectrum of gambaoic B methyl ester (**4**) in CD_3_OD 22

**Figure S20.** ^13^C NMR spectrum of gambaoic B methyl ester (**4**) in CD_3_OD 23

**Figure S21.** ^1^H-^1^H COSY gambaoic B methyl ester (**4**) in CD_3_OD 24

**Figure S22.** HSQC NMR spectrum of gambaoic B methyl ester (**4**) in CD_3_OD 25

**Figure S23.** HMBC NMR spectrum of gambaoic B methyl ester (**4**) in CD_3_OD 26

**Figure S24.** HRMS spectrum of gambaoic B methyl ester (**4**) 27

**Table S1.** NMR table of compounds **1**–**4** 28

1. **Computational NMR Chemical Shift Calculations for DP4+ Analyses data**

**Figure S25.** Computational calculation of NMR (A) Experimental ^1^H and ^13^C chemical shift of compound **2**, and calculated shielding tensors values of possible stereoisomers [**2a** (14*S*^*^, 15*S*^*^, isomer 1) and **2b** (14*S*^*^, 15*R*^*^, isomer 2)], (B) DP4+ analysis results of **2** 29

**Figure S26.** Computational calculation of NMR (A) Experimental ^1^H and ^13^C chemical shift of compound **3**, and calculated shielding tensors values of possible stereoisomers [**3a** (14*R*^*^, 15*R*^*^, isomer 1) and **3b** (14*S*^*^, 15*R*^*^, isomer 2)], (B) DP4+ analysis results of **3** 31

**Table S2.** NMR calculation of **2a** (14*S*^*^, 15*S*^*^), (A) Boltzmann distribution of energy minimized conformers, (B) Optimized Z-matrixes of **2a** conformers in the MeOH (Ǻ) 33

**Table S3.** NMR calculation of **2b** (14*S*^*^, 15*R*^*^), (A) Boltzmann distribution of energy minimized conformers, (B) Optimized Z-matrixes of **2b** conformers in the MeOH (Ǻ) 47

**Table S4.** Specific rotation calculation of **2a** (14*S*, 15*S*), (A) Boltzmann distribution of energy minimized conformers, and calculated optical rotation value (B) Optimized Z-matrixes of **2a** conformers in the MeOH (Ǻ) 60

**Table S5.** NMR calculation of **3a** (14*R*^*^, 15*R*^*^), (A) Boltzmann distribution of energy minimized conformers, (B) Optimized Z-matrixes of **3a** conformers in the MeOH (Ǻ) 74

**Table S6.** NMR calculation of **3b** (14*S*^*^, 15*R*^*^), (A) Boltzmann distribution of energy minimized conformers, (B) Optimized Z-matrixes of **3b** conformers in the MeOH (Ǻ) 90

**Table S7.** Specific rotation calculation of **3a** (14*R*, 15*R*), (A) Boltzmann distribution of energy minimized conformers, and calculated optical rotation value, (B) Optimized Z-matrixes of **3a** conformers in the MeOH (Ǻ) 103

1. **NMR spectra and HRMS data**

**Figure S1.** ^1^H NMR spectrum of gambaoic acid A (**1**) in CD_3_OD

**Figure S2.** ^13^C NMR spectrum of gambaoic acid A (**1**) in CD_3_OD

**Figure S3.** ^1^H-^1^H COSY gambaoic acid A (**1**) in CD_3_OD

**Figure S4.** HSQC NMR spectrum of gambaoic acid A (**1**) in CD_3_OD

**Figure S5.** HMBC NMR spectrum of gambaoic acid A (**1**) in CD_3_OD

**Figure S6.** HRMS spectrum of gambaoic acid A (**1**)

*_
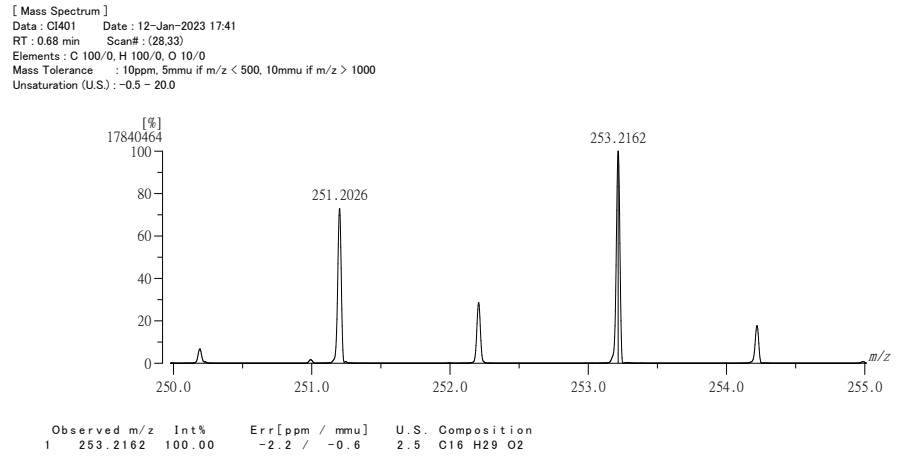
_*

**Figure S7.** ^1^H NMR spectrum of gambaoic acid B (**2**) in CD_3_OD

**Figure S8.** ^13^C NMR spectrum of gambaoic acid B (**2**) in CD_3_OD

**Figure S9.** ^1^H-^1^H COSY gambaoic acid B (**2**) in CD_3_OD

**Figure S10.** HSQC NMR spectrum of gambaoic acid B (**2**) in CD_3_OD

**Figure S11.** HMBC NMR spectrum of gambaoic acid B (**2**) in CD_3_OD

**Figure S12.** HRMS spectrum of gambaoic acid B (**2**)

*_
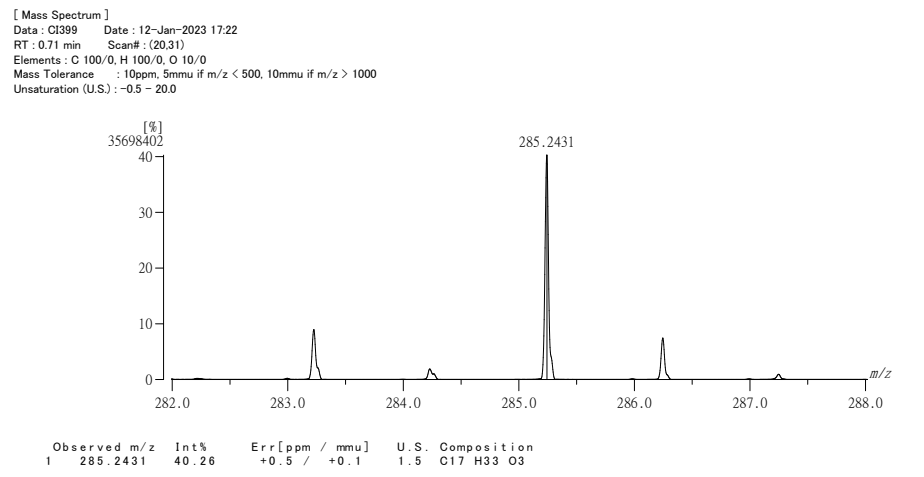
_*

**Figure S13.** ^1^H NMR spectrum of gambaoic acid C (**3**) in CD_3_OD

**Figure S14.** ^13^C NMR spectrum of gambaoic acid C (**3**) in CD_3_OD

**Figure S15.** ^1^H-^1^H COSY gambaoic acid C (**3**) in CD_3_OD

**Figure S16.** HSQC NMR spectrum of gambaoic acid C (**3**) in CD_3_OD

**Figure S17.** HMBC NMR spectrum of gambaoic acid C (**3**) in CD_3_OD

*****__*

**Figure S18.** HRMS spectrum of gambaoic acid C (**3**)

**Figure S19.** ^1^H NMR spectrum of gambaoic B methyl ester (**4**) in CD_3_OD

**Figure S20.** ^13^C NMR spectrum of gambaoic B methyl ester (**4**) in CD_3_OD

**Figure S21.** ^1^H-^1^H COSY spectrum of gambaoic B methyl ester (**4**) in CD_3_OD

**Figure S22.** HSQC NMR spectrum of gambaoic B methyl ester (**4**) in CD_3_OD

**Figure S23.** HMBC NMR spectrum of gambaoic B methyl ester (**4**) in CD_3_OD

**Figure S24.** HRMS spectrum of gambaoic B methyl ester (**4**)

**Table S1.** NMR table of compounds **1**–**4**

| No. | **1** | | | | **2** | | **3** | | | | **4** | |
| --- | --- | --- | --- | --- | --- | --- | --- | --- | --- | --- | --- | --- |
|  | δ_C_ ^a^, type^b^ | δ_H_ ^a^ (*J* in Hz) | COSY | HMBC (^1^H to ^13^C) | δ_C_ ^a^, type^b^ | δ_H_ ^a^ (*J* in Hz) | δ_C_ ^a^, type^b^ | δ_H_ ^a^ (*J* in Hz) | COSY | HMBC  (^1^H to ^13^C) | δ_C_ ^a^, type^b^ | δ_H_ ^a^ (*J* in Hz) |
| 1 | 177.8, qC |  |  |  | 177.7, qC |  | 177.8, qC |  |  |  | 176.0, qC |  |
| 2 | 34.4, CH_2_ | 2.28, td (7.4, 4.6) | 3 | C-1, 3, 4 | 35.0, CH_2_ | 2.28, t (7.4) | 34.5, CH_2_ | 2.28, t (7.4) | 3 | C-1, 3, 4 | 34.8, CH_2_ | 2.31, t (7.6) |
| 3 | 26.1, CH_2_ | 1.65, m | 2, 4 | C-1, 2, 4, 5 | 26.1, CH_2_ | 1.60, m | 26.2, CH_2_ | 1.65, m | 2, 4 | C-1, 2, 4 | 26.0, CH_2_ | 1.59, t (7.6) |
| 4 | 27.5, CH_2_ | 2.08, m | 3, 5 | C-2, 3, 5, 6 | 30.2, CH_2_ | 1.31, m | 27.6, CH_2_ | 2.08, m | 3, 4 |  | 30.2, CH_2_ | 1.30, m |
| 5 | 129.7, CH | 5.35, ttd (11.3) | 4, 6 |  | 30.4, CH_2_ | 1.31, m | 129.7, CH | 5.35, ttd (11.3) | 4 |  | 30.4, CH_2_ | 1.30, m |
| 6 | 131.9, CH | 5.41, ttd (11.3) | 5, 7 |  | 30.6, CH_2_ | 1.31, m | 131.9, CH | 5.41, ttd (11.3) | 7 |  | 30.6, CH_2_ | 1.30, m |
| 7 | 28.1, CH_2_ | 2.05, m | 6 |  | 30.7, CH_2_ | 1.31, m | 28.1, CH_2_ | 2.05, m | 6 |  | 30.7, CH_2_ | 1.30, m |
| 8 | 30.3, CH_2_ | 1.33, m |  |  | 30.7, CH_2_ | 1.31, m | 30.7, CH_2_ | 1.33, m |  |  | 30.7, CH_2_ | 1.30, m |
| 9 | 30.6, CH_2_ | 1.33, m |  |  | 30.7, CH_2_ | 1.31, m | 30.7, CH_2_ | 1.33, m |  |  | 30.7, CH_2_ | 1.30, m |
| 10 | 30.8, CH_2_ | 1.33, m |  |  | 30.8, CH_2_ | 1.31, m | 30.8, CH_2_ | 1.33, m |  |  | 30.8, CH_2_ | 1.30, m |
| 11 | 25.4, CH_2_ | 1.34, m |  |  | 31.6, CH_2_ | 1.31, m | 31.5, CH_2_ | 1.31, m |  |  | 31.6, CH_2_ | 1.30, m |
| 12 | 31.4, CH_2_ | 1.39, m |  |  | 24.4, CH_2_ | 1.39, m | 24.4, CH_2_ | 1.39, m |  |  | 24.4, CH_2_ | 1.38, m |
| 13 | 44.9, CH_2_ | 1.43, m |  | C-11, 14 | 39.2, CH_2_ | 1.40, m | 39.2, CH_2_ | 1.40, 1.48 |  |  | 39.1, CH_2_ | 1.39, m |
| 14 | 71.5, qC |  |  |  | 75.7, qC |  | 75.7, qC |  |  |  | 75.7, qC |  |
| 15 | 29.1, CH_3_ | 1.17, s | 16 | C-14, 16 | 74.1, CH | 3.56, q (6.5) | 74.1, CH | 3.56, q (6.5) | 16 | C-13, 14, 16, 17 | 74.1, CH | 3.56, q (6.5) |
| 16 | 29.1, CH_3_ | 1.17, s | 15 | C-14, 15 | 17.6, CH_3_ | 1.12, d (6.5) | 17.6, CH_3_ | 1.12, d (6.5) | 15 | C-14, 15 | 17.5, CH_3_ | 1.11, d (6.5) |
| 17 |  |  |  |  | 21.6, CH_3_ | 1.08, s | 21.6, CH_3_ | 1.08, s |  | C-13, 14, 15 | 21.6, CH_3_ | 1.08, s |
| 18 |  |  |  |  |  |  |  |  |  |  | 51.9, CH_3_ | 3.65, s |
| ^a^ 400MHz for ^1^H NMR and 100MHz for ^13^C NMR.  ^b^ Numbers of attached protons were determined by analysis of 2D spectra | | | | | | | | | | | | |

1. **Computational NMR Chemical Shift Calculations for DP4+ Analyses data**

**Figure S25.** Computational calculation of NMR (A) Experimental ^1^H and ^13^C chemical shift of compound **2**, and calculated shielding tensors values of possible stereoisomers [**2a** (14*S*^*^, 15*S*^*^, isomer 1) and **2b** (14*S*^*^, 15*R*^*^, isomer 2)], (B) DP4^+^ analysis results of **2**

(A)

**
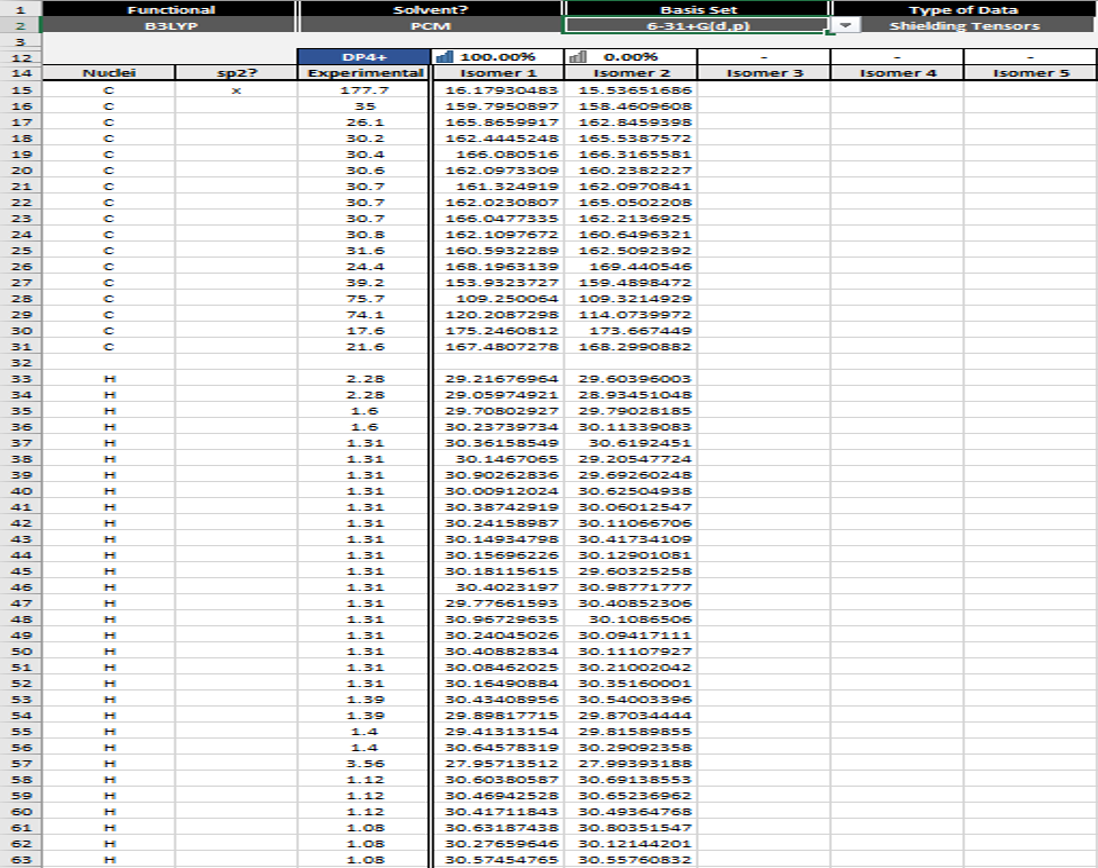
**

(B)

**
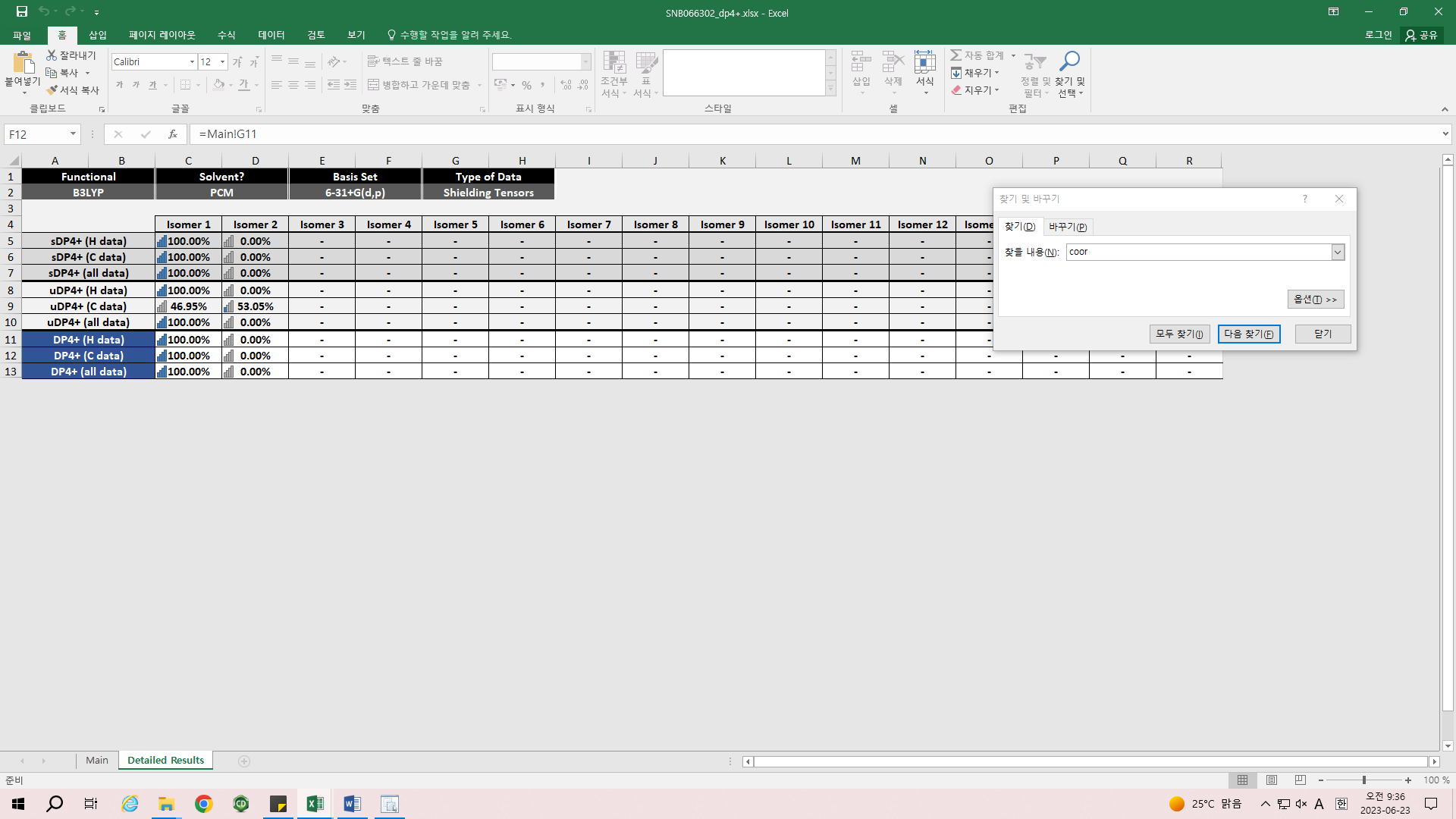
**

**Figure S26.** Computational calculation of NMR (A) Experimental ^1^H and ^13^C chemical shift of compound **3**, and calculated shielding tensors values of possible stereoisomers [**3a** (14*R*^*^, 15*R*^*^, isomer 1) and **3b** (14*S*^*^, 15*R*^*^, isomer 2)], (B) DP4^+^ analysis results of **3**

(A)

**
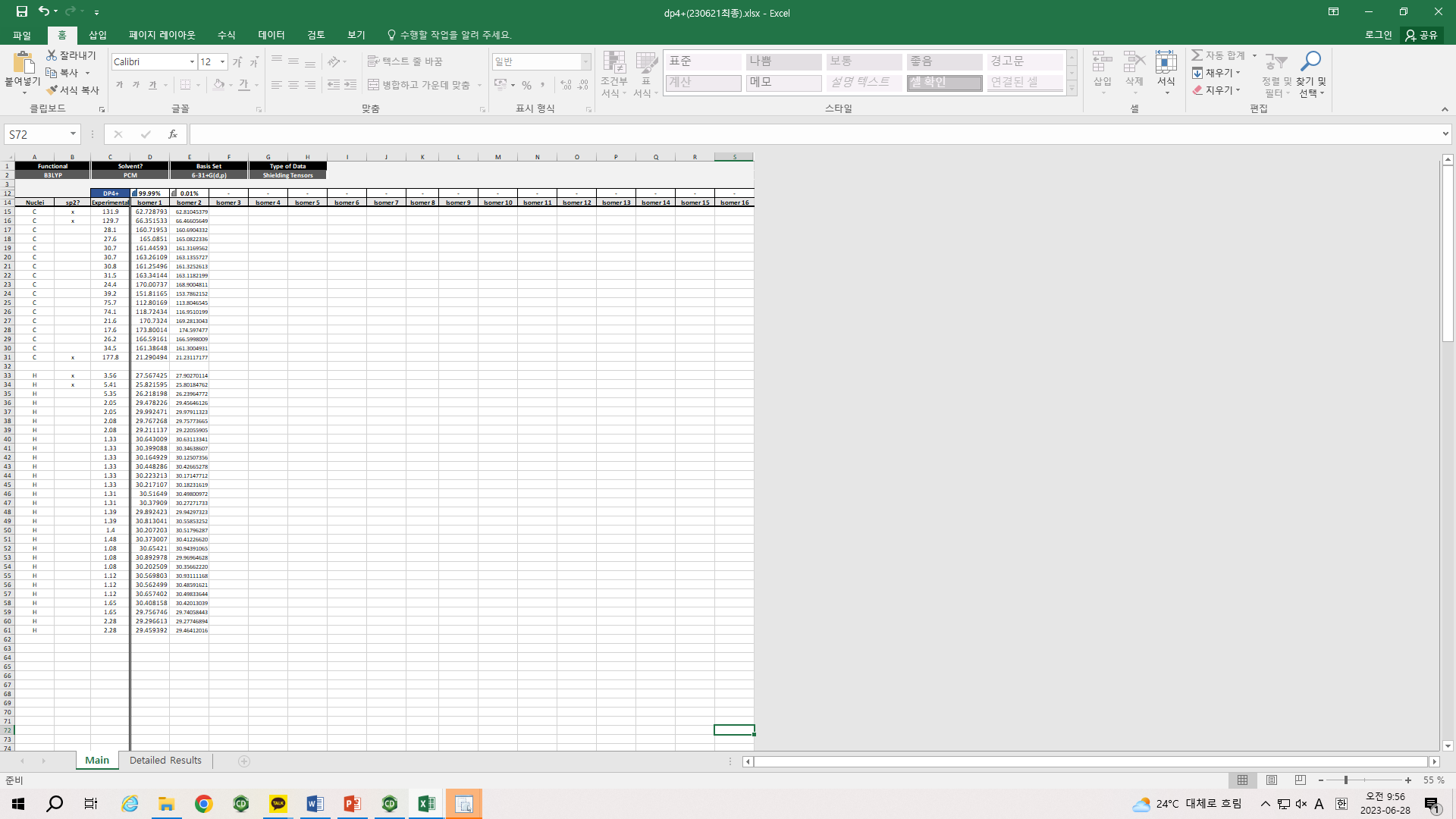
**

(B)

**
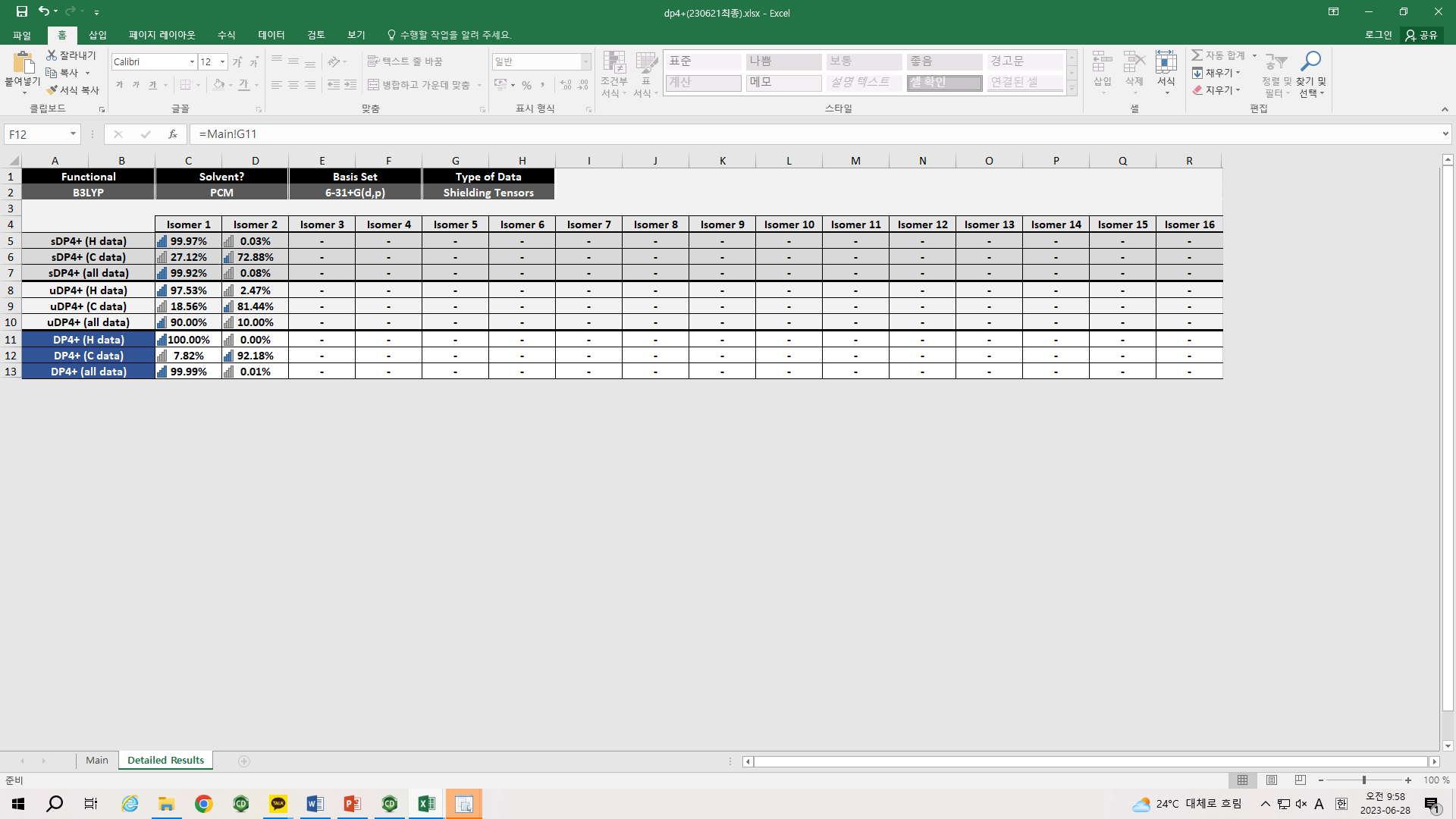
**

**Table S2.** NMR calculation of **2a** (14*S*^*^, 15*S*^*^), (A) Boltzmann distribution of energy minimized conformers, (B) Optimized Z-matrixes of **2a** conformers in the MeOH (Ǻ)

(A)

| Conformer | Calculated Energy (G)  (atomic units) | Relative Energy (kcal/mol) | Boltzmann Weights (%) |
| --- | --- | --- | --- |
| 1 | -969.292698 | 0.000000 | 99.131756126 |
| 2 | -969.288441 | 2.671308 | 0.000000520 |
| 3 | -969.288226 | 2.806222 | 0.000000199 |
| 4 | -969.290345 | 1.476530 | 0.002627227 |
| 5 | -969.289984 | 1.703061 | 0.000521594 |
| 6 | -969.289107 | 2.253387 | 0.000010269 |
| 7 | -969.291355 | 0.842745 | 0.242104861 |
| 8 | -969.287348 | 3.357176 | 0.000000004 |
| 9 | -969.291225 | 0.924321 | 0.135252817 |
| 10 | -969.288843 | 2.419049 | 0.000003148 |
| 11 | -969.288293 | 2.764179 | 0.000000268 |
| 12 | -969.291044 | 1.037901 | 0.060130014 |
| 13 | -969.291482 | 0.763052 | 0.427588026 |
| 14 | -969.288943 | 2.356298 | 0.000004926 |

(B)

| **Conformer 1** | | | | | | | |
| --- | --- | --- | --- | --- | --- | --- | --- |
| Atom | X | Y | Z | Atom | X | Y | Z |
| C | 2.526436 | -0.5454 | 1.299953 | H | -0.4448 | 4.230752 | -1.38016 |
| C | 2.905285 | -0.13136 | -0.15166 | H | -0.09297 | 2.55575 | -1.74691 |
| C | 2.043553 | 1.042636 | -0.65601 | H | -1.09499 | 3.555709 | 0.974384 |
| C | 2.018183 | 2.291316 | 0.23608 | H | -0.78925 | 1.878913 | 0.552306 |
| C | 1.342146 | 3.506777 | -0.42652 | H | -2.83768 | 3.708046 | -0.8112 |
| C | -0.09545 | 3.290991 | -0.93093 | H | -2.5237 | 2.053707 | -1.28579 |
| C | -1.10409 | 2.843423 | 0.136174 | H | -4.57447 | 2.281017 | 0.10437 |
| C | -2.53123 | 2.728553 | -0.41933 | H | -3.64507 | 2.988878 | 1.41333 |
| C | -3.59098 | 2.259246 | 0.593711 | H | -2.4254 | 0.849687 | 1.762135 |
| C | -3.3678 | 0.861779 | 1.198629 | H | -4.15796 | 0.671723 | 1.937656 |
| C | -3.35134 | -0.28825 | 0.181492 | H | -2.59082 | -0.08355 | -0.5818 |
| C | -3.06065 | -1.6445 | 0.838058 | H | -4.314 | -0.32623 | -0.34895 |
| C | -3.02751 | -2.84974 | -0.11824 | H | -2.10034 | -1.58275 | 1.363955 |
| C | -2.05027 | -2.72804 | -1.2989 | H | -3.81739 | -1.84146 | 1.608992 |
| C | -0.61783 | -2.46594 | -0.88768 | H | -4.02772 | -3.01852 | -0.53552 |
| O | 0.073488 | -1.79465 | -1.80498 | H | -2.77444 | -3.74654 | 0.457662 |
| C | 3.212324 | -1.82505 | 1.792075 | H | -2.04874 | -3.66421 | -1.87304 |
| O | 1.116152 | -0.64856 | 1.466286 | H | -2.35256 | -1.94663 | -2.00105 |
| O | 2.581093 | -1.21522 | -1.07709 | H | 1.009989 | -1.64172 | -1.48651 |
| C | 4.402286 | 0.171651 | -0.28216 | H | 4.30265 | -1.74573 | 1.790565 |
| O | -0.13381 | -2.83683 | 0.178901 | H | 2.889592 | -2.03029 | 2.816089 |
| H | 2.837024 | 0.282509 | 1.947849 | H | 2.926051 | -2.68712 | 1.178758 |
| H | 1.023606 | 0.669326 | -0.77882 | H | 0.814687 | -1.4666 | 1.025595 |
| H | 2.407502 | 1.307304 | -1.65722 | H | 3.183718 | -1.95681 | -0.92427 |
| H | 1.505813 | 2.051746 | 1.173941 | H | 4.6296 | 0.495097 | -1.30174 |
| H | 3.040273 | 2.582862 | 0.5087 | H | 4.707527 | 0.964705 | 0.406328 |
| H | 1.959298 | 3.835951 | -1.2736 | H | 5.014313 | -0.70854 | -0.06254 |
| H | 1.34684 | 4.336606 | 0.293046 |  |  |  |  |

**Table S2:** (continued)

| **Conformer 2** | | | | | | | |
| --- | --- | --- | --- | --- | --- | --- | --- |
| Atom | X | Y | Z | Atom | X | Y | Z |
| C | 3.281605 | 0.793567 | -0.52134 | H | -0.64058 | -2.31093 | -1.07533 |
| C | 3.140527 | -0.69098 | -0.07968 | H | -0.24832 | -3.96538 | -0.65801 |
| C | 1.647181 | -1.00831 | 0.170153 | H | -2.42167 | -4.19989 | 0.553682 |
| C | 1.347105 | -2.3131 | 0.92467 | H | -2.59532 | -3.88007 | -1.16327 |
| C | -0.15891 | -2.62307 | 1.020615 | H | -4.25244 | -2.65151 | 0.191057 |
| C | -0.78927 | -3.07681 | -0.30477 | H | -2.98844 | -1.82418 | 1.075956 |
| C | -2.28555 | -3.42442 | -0.21279 | H | -3.34883 | -1.61109 | -1.96046 |
| C | -3.23344 | -2.25108 | 0.095482 | H | -2.26507 | -0.63747 | -0.98957 |
| C | -3.23744 | -1.14527 | -0.97128 | H | -4.32258 | 0.589534 | -1.65262 |
| C | -4.35892 | -0.10475 | -0.80351 | H | -5.3281 | -0.61682 | -0.87819 |
| C | -4.34128 | 0.695407 | 0.512459 | H | -4.50221 | 0.01073 | 1.354485 |
| C | -3.06582 | 1.510372 | 0.783949 | H | -5.20285 | 1.377185 | 0.51358 |
| C | -2.79341 | 2.627112 | -0.23336 | H | -3.14657 | 1.95873 | 1.783403 |
| C | -1.54546 | 3.454459 | 0.106212 | H | -2.19888 | 0.83793 | 0.825741 |
| C | -0.25457 | 2.675557 | -0.07532 | H | -3.65931 | 3.299243 | -0.27642 |
| O | 0.634085 | 2.91228 | 0.891551 | H | -2.66611 | 2.209019 | -1.23725 |
| C | 4.662067 | 1.188649 | -1.04226 | H | -1.48037 | 4.321481 | -0.56406 |
| O | 2.955227 | 1.633685 | 0.607496 | H | -1.59112 | 3.844471 | 1.127104 |
| O | 3.891898 | -0.73445 | 1.156253 | H | 1.474013 | 2.395684 | 0.71941 |
| C | 3.758099 | -1.65312 | -1.10038 | H | 4.887498 | 0.703194 | -1.99554 |
| O | -0.04209 | 1.92719 | -1.01935 | H | 4.689378 | 2.270396 | -1.19865 |
| H | 2.518732 | 1.004113 | -1.27672 | H | 5.441709 | 0.923168 | -0.32196 |
| H | 1.15175 | -1.0197 | -0.80675 | H | 3.281654 | 1.148993 | 1.387007 |
| H | 1.206386 | -0.1761 | 0.728367 | H | 3.712721 | -1.57485 | 1.59823 |
| H | 1.851245 | -3.16543 | 0.450553 | H | 3.598189 | -2.69258 | -0.79981 |
| H | 1.747545 | -2.24132 | 1.945007 | H | 3.305574 | -1.51907 | -2.08848 |
| H | -0.67985 | -1.73674 | 1.406111 | H | 4.834624 | -1.49178 | -1.18455 |
| H | -0.3105 | -3.4139 | 1.767303 |  |  |  |  |

**Table S2:** (continued)

| **Conformer 3** | | | | | | | |
| --- | --- | --- | --- | --- | --- | --- | --- |
| Atom | X | Y | Z | Atom | X | Y | Z |
| C | 3.433835 | -0.40388 | 0.996869 | H | -0.55676 | 2.39889 | 0.993712 |
| C | 2.991874 | 0.373051 | -0.27148 | H | -0.22054 | 3.897434 | 0.151679 |
| C | 1.680901 | 1.133876 | -0.02369 | H | -2.51539 | 4.040432 | -0.69317 |
| C | 1.128898 | 1.893795 | -1.24448 | H | -2.50023 | 3.89662 | 1.056244 |
| C | -0.37916 | 2.183185 | -1.15224 | H | -4.31306 | 2.570228 | 0.083511 |
| C | -0.80912 | 2.971159 | 0.092823 | H | -3.20772 | 1.700401 | -0.95724 |
| C | -2.30317 | 3.3424 | 0.128319 | H | -3.11861 | 1.587726 | 2.105384 |
| C | -3.29252 | 2.165714 | 0.032338 | H | -2.14631 | 0.614326 | 1.02406 |
| C | -3.12684 | 1.096698 | 1.122318 | H | -4.03005 | -0.67402 | 1.958388 |
| C | -4.22793 | 0.019798 | 1.131345 | H | -5.18873 | 0.497814 | 1.366401 |
| C | -4.40148 | -0.77511 | -0.17776 | H | -4.75009 | -0.0969 | -0.96622 |
| C | -3.15106 | -1.5094 | -0.68958 | H | -5.20964 | -1.50553 | -0.03505 |
| C | -2.66414 | -2.65403 | 0.209486 | H | -3.36727 | -1.91705 | -1.68605 |
| C | -1.37727 | -3.31799 | -0.314 | H | -2.33671 | -0.78745 | -0.8351 |
| C | -0.17475 | -2.40393 | -0.16635 | H | -3.44897 | -3.41618 | 0.286004 |
| O | 0.487834 | -2.20791 | -1.30716 | H | -2.47296 | -2.29391 | 1.22599 |
| C | 3.939719 | 0.44227 | 2.161255 | H | -1.16303 | -4.21849 | 0.273735 |
| O | 4.473162 | -1.29933 | 0.540821 | H | -1.48557 | -3.62139 | -1.35882 |
| O | 2.702195 | -0.65011 | -1.26828 | H | 1.270652 | -1.60265 | -1.16156 |
| C | 4.110073 | 1.26777 | -0.82097 | H | 3.175274 | 1.14087 | 2.513605 |
| O | 0.145394 | -1.89542 | 0.900302 | H | 4.205263 | -0.20756 | 3.001279 |
| H | 2.566455 | -0.99545 | 1.31502 | H | 4.830295 | 1.010511 | 1.879388 |
| H | 1.837817 | 1.833375 | 0.804002 | H | 4.486905 | -2.06709 | 1.126889 |
| H | 0.941562 | 0.402198 | 0.321555 | H | 3.430607 | -1.28957 | -1.15881 |
| H | 1.675059 | 2.836936 | -1.37383 | H | 3.83303 | 1.651612 | -1.80527 |
| H | 1.311137 | 1.302927 | -2.14945 | H | 4.296281 | 2.120783 | -0.16169 |
| H | -0.91503 | 1.22547 | -1.18195 | H | 5.040437 | 0.702081 | -0.92157 |
| H | -0.69001 | 2.732401 | -2.05176 |  |  |  |  |

**Table S2:** (continued)

| **Conformer 4** | | | | | | | |
| --- | --- | --- | --- | --- | --- | --- | --- |
| Atom | X | Y | Z | Atom | X | Y | Z |
| C | -4.39174 | 0.373 | -0.40105 | H | 0.781825 | 1.35224 | 0.717554 |
| C | -3.06446 | -0.17716 | 0.176139 | H | 1.009991 | 1.655722 | -0.99352 |
| C | -2.06386 | 0.919936 | 0.580568 | H | 1.754298 | 3.634984 | 1.228086 |
| C | -1.70718 | 1.955315 | -0.4979 | H | 2.129732 | 3.829871 | -0.47645 |
| C | -0.53007 | 2.869283 | -0.10816 | H | 4.060723 | 2.999025 | 0.997852 |
| C | 0.825056 | 2.152742 | -0.03192 | H | 3.142725 | 1.53332 | 1.279796 |
| C | 2.002412 | 3.077113 | 0.314872 | H | 4.326203 | 2.531501 | -1.3607 |
| C | 3.325699 | 2.323544 | 0.54008 | H | 3.152468 | 1.238111 | -1.33065 |
| C | 3.936953 | 1.71763 | -0.73444 | H | 5.476847 | 0.396952 | -1.46036 |
| C | 5.066194 | 0.699845 | -0.48758 | H | 5.888866 | 1.192402 | 0.048667 |
| C | 4.658243 | -0.56541 | 0.293076 | H | 4.400985 | -0.29827 | 1.326485 |
| C | 3.486078 | -1.33976 | -0.32356 | H | 5.53222 | -1.22674 | 0.366102 |
| C | 3.08463 | -2.58802 | 0.473108 | H | 2.614259 | -0.67879 | -0.39218 |
| C | 1.818761 | -3.2632 | -0.07381 | H | 3.734734 | -1.62776 | -1.35512 |
| C | 0.574409 | -2.41064 | 0.11259 | H | 2.907021 | -2.31386 | 1.519341 |
| O | -0.28444 | -2.5178 | -0.90246 | H | 3.90514 | -3.31543 | 0.469872 |
| C | -5.27979 | 1.123619 | 0.586153 | H | 1.923934 | -3.5147 | -1.13332 |
| O | -5.07454 | -0.78294 | -0.93129 | H | 1.635511 | -4.20621 | 0.45787 |
| O | -2.45296 | -0.91795 | -0.91611 | H | -1.07599 | -1.9268 | -0.75322 |
| C | -3.29619 | -1.14196 | 1.347884 | H | -4.76349 | 1.992124 | 1.006183 |
| O | 0.372642 | -1.71365 | 1.09783 | H | -6.17783 | 1.485978 | 0.075952 |
| H | -4.13252 | 1.037051 | -1.23457 | H | -5.59539 | 0.47427 | 1.406922 |
| H | -1.16044 | 0.390819 | 0.900189 | H | -5.69187 | -0.48131 | -1.61056 |
| H | -2.45158 | 1.432673 | 1.469408 | H | -3.19654 | -1.38457 | -1.33858 |
| H | -1.46623 | 1.432643 | -1.43174 | H | -2.3436 | -1.59858 | 1.628349 |
| H | -2.57872 | 2.586316 | -0.71156 | H | -3.6965 | -0.62159 | 2.222951 |
| H | -0.74458 | 3.350827 | 0.856905 | H | -3.99847 | -1.93145 | 1.066915 |
| H | -0.4603 | 3.682631 | -0.84299 |  |  |  |  |

**Table S2:** (continued)

| **Conformer 5** | | | | | | | |
| --- | --- | --- | --- | --- | --- | --- | --- |
| Atom | X | Y | Z | Atom | X | Y | Z |
| C | 3.953923 | -0.03028 | -0.24697 | H | -2.12526 | -3.64465 | -1.73826 |
| C | 2.848557 | -0.92294 | 0.412337 | H | -1.22829 | -4.13507 | -0.31346 |
| C | 1.781846 | -1.27937 | -0.65284 | H | -2.18498 | -2.11103 | 0.913308 |
| C | 0.532396 | -2.01166 | -0.14138 | H | -3.34079 | -3.34753 | 0.464168 |
| C | -0.4359 | -2.37949 | -1.27701 | H | -4.02408 | -2.04064 | -1.53882 |
| C | -1.62469 | -3.25647 | -0.84055 | H | -2.89997 | -0.76647 | -1.1236 |
| C | -2.67908 | -2.57405 | 0.050306 | H | -5.2747 | -1.70283 | 0.566601 |
| C | -3.54109 | -1.53909 | -0.68834 | H | -5.3016 | -0.29813 | -0.47888 |
| C | -4.64563 | -0.89297 | 0.171938 | H | -5.02005 | 0.023462 | 2.081067 |
| C | -4.19566 | -0.01259 | 1.357326 | H | -3.36303 | -0.49568 | 1.885382 |
| C | -3.8074 | 1.442289 | 1.035459 | H | -3.60833 | 1.952011 | 1.988623 |
| C | -2.58647 | 1.623876 | 0.122238 | H | -4.67084 | 1.959374 | 0.593049 |
| C | -2.03054 | 3.053634 | 0.126729 | H | -1.79226 | 0.936741 | 0.437541 |
| C | -0.86191 | 3.251097 | -0.84392 | H | -2.84689 | 1.350959 | -0.90789 |
| C | 0.379303 | 2.461358 | -0.48789 | H | -1.70512 | 3.317927 | 1.139579 |
| O | 1.222981 | 2.335307 | -1.51155 | H | -2.8236 | 3.761236 | -0.14434 |
| C | 5.26883 | 0.06117 | 0.523258 | H | -1.14384 | 2.993015 | -1.87058 |
| O | 3.442949 | 1.314611 | -0.42163 | H | -0.55754 | 4.305918 | -0.87408 |
| O | 2.275998 | -0.18712 | 1.50505 | H | 2.053328 | 1.87775 | -1.19432 |
| C | 3.456314 | -2.18866 | 1.024395 | H | 5.800401 | -0.89325 | 0.529958 |
| O | 0.605039 | 2.002367 | 0.628791 | H | 5.913509 | 0.807647 | 0.051913 |
| H | 4.146571 | -0.39051 | -1.26234 | H | 5.090147 | 0.362655 | 1.561143 |
| H | 2.271731 | -1.89528 | -1.41882 | H | 3.259251 | 1.640661 | 0.475169 |
| H | 1.470851 | -0.35931 | -1.16312 | H | 1.65325 | 0.474618 | 1.147813 |
| H | 0.824391 | -2.93479 | 0.374678 | H | 2.671808 | -2.83116 | 1.429391 |
| H | 0.02787 | -1.38955 | 0.606691 | H | 4.01445 | -2.75909 | 0.275281 |
| H | 0.12567 | -2.92507 | -2.0476 | H | 4.13287 | -1.93603 | 1.843611 |
| H | -0.79752 | -1.46436 | -1.76468 |  |  |  |  |

**Table S2:** (continued)

| **Conformer 6** | | | | | | | |
| --- | --- | --- | --- | --- | --- | --- | --- |
| Atom | X | Y | Z | Atom | X | Y | Z |
| C | 3.547889 | -0.73669 | 0.913647 | H | -1.10943 | 1.885917 | -1.44386 |
| C | 3.202777 | 0.250769 | -0.23251 | H | -0.75071 | 1.334779 | 0.179975 |
| C | 2.115011 | 1.242379 | 0.207 | H | -2.25469 | 3.912122 | -0.51609 |
| C | 1.640212 | 2.226113 | -0.876 | H | -1.90274 | 3.375133 | 1.118707 |
| C | 0.409137 | 3.046576 | -0.44663 | H | -4.26543 | 2.853759 | 0.291775 |
| C | -0.8945 | 2.237073 | -0.42383 | H | -3.56303 | 1.780142 | -0.90326 |
| C | -2.11036 | 3.014599 | 0.100675 | H | -3.41728 | 1.501417 | 2.146396 |
| C | -3.4106 | 2.189987 | 0.104024 | H | -2.49758 | 0.4761 | 1.069817 |
| C | -3.42421 | 1.056175 | 1.14203 | H | -4.54789 | -0.62782 | 1.867398 |
| C | -4.61864 | 0.091383 | 1.041395 | H | -5.55284 | 0.644962 | 1.208015 |
| C | -4.74363 | -0.67454 | -0.29231 | H | -5.07198 | 0.016694 | -1.07844 |
| C | -3.46677 | -1.37905 | -0.78136 | H | -5.54923 | -1.4147 | -0.19263 |
| C | -2.92433 | -2.46961 | 0.152711 | H | -3.66885 | -1.82748 | -1.76328 |
| C | -1.59227 | -3.05916 | -0.33899 | H | -2.68391 | -0.62917 | -0.95321 |
| C | -0.43804 | -2.08132 | -0.19929 | H | -3.6599 | -3.27834 | 0.239709 |
| O | 0.33445 | -2.02432 | -1.28569 | H | -2.77042 | -2.07258 | 1.161339 |
| C | 4.29223 | -0.1477 | 2.108229 | H | -1.32874 | -3.93656 | 0.265213 |
| O | 4.339995 | -1.7695 | 0.288944 | H | -1.66298 | -3.39211 | -1.37826 |
| O | 2.628667 | -0.57067 | -1.28968 | H | 1.101319 | -1.40272 | -1.13913 |
| C | 4.454339 | 0.938501 | -0.79403 | H | 3.711544 | 0.640349 | 2.596218 |
| O | -0.22832 | -1.42672 | 0.813455 | H | 4.475169 | -0.93121 | 2.850448 |
| H | 2.595588 | -1.16885 | 1.248738 | H | 5.259072 | 0.26432 | 1.806851 |
| H | 2.49463 | 1.817272 | 1.060069 | H | 4.250885 | -2.5756 | 0.813737 |
| H | 1.269324 | 0.65141 | 0.573223 | H | 3.227931 | -1.33874 | -1.33268 |
| H | 2.456032 | 2.913979 | -1.12664 | H | 4.210696 | 1.460849 | -1.72164 |
| H | 1.405531 | 1.67561 | -1.79566 | H | 4.858397 | 1.668339 | -0.08626 |
| H | 0.288949 | 3.897811 | -1.12988 | H | 5.233298 | 0.201881 | -1.00907 |
| H | 0.591346 | 3.479797 | 0.547666 |  |  |  |  |

**Table S2:** (continued)

| **Conformer 7** | | | | | | | |
| --- | --- | --- | --- | --- | --- | --- | --- |
| Atom | X | Y | Z | Atom | X | Y | Z |
| C | -2.35434 | -0.22019 | 1.006927 | H | 0.654572 | -3.81889 | 0.376821 |
| C | -2.7071 | -1.11242 | -0.22209 | H | 2.037912 | -4.1846 | -0.63138 |
| C | -1.5984 | -2.14541 | -0.53333 | H | 2.58938 | -2.9728 | 1.512692 |
| C | -0.30149 | -1.59367 | -1.14491 | H | 1.682782 | -1.58642 | 0.953295 |
| C | 0.736971 | -2.68515 | -1.47046 | H | 4.19494 | -2.76716 | -0.34925 |
| C | 1.4185 | -3.35623 | -0.26113 | H | 3.288109 | -1.44968 | -1.05321 |
| C | 2.291225 | -2.42964 | 0.605501 | H | 5.294558 | -0.65705 | 0.17073 |
| C | 3.556545 | -1.91001 | -0.09516 | H | 4.727963 | -1.40719 | 1.652534 |
| C | 4.386187 | -0.91048 | 0.734197 | H | 2.778759 | 0.139037 | 1.73544 |
| C | 3.656124 | 0.388404 | 1.124516 | H | 4.310411 | 0.980871 | 1.777378 |
| C | 3.205457 | 1.255191 | -0.06169 | H | 2.758798 | 0.620073 | -0.83673 |
| C | 2.166742 | 2.313651 | 0.327618 | H | 4.07925 | 1.731722 | -0.52757 |
| C | 1.649663 | 3.133933 | -0.86462 | H | 1.323235 | 1.795768 | 0.805607 |
| C | 0.316366 | 3.845521 | -0.57134 | H | 2.574437 | 2.988605 | 1.092378 |
| C | -0.80139 | 2.837129 | -0.39072 | H | 1.495951 | 2.474934 | -1.72675 |
| O | -1.48384 | 2.987938 | 0.739345 | H | 2.395809 | 3.878245 | -1.16422 |
| C | -2.49885 | -0.865 | 2.37777 | H | 0.384608 | 4.47867 | 0.317031 |
| O | -3.20301 | 0.957745 | 0.968725 | H | 0.041957 | 4.485854 | -1.41793 |
| O | -2.91218 | -0.21325 | -1.33544 | H | -2.18022 | 2.268081 | 0.826205 |
| C | -4.05091 | -1.82559 | -0.04291 | H | -1.85295 | -1.74286 | 2.468712 |
| O | -1.02965 | 1.953654 | -1.21392 | H | -2.20369 | -0.15156 | 3.152648 |
| H | -1.31692 | 0.117619 | 0.884704 | H | -3.53174 | -1.16655 | 2.568489 |
| H | -2.02138 | -2.87649 | -1.23435 | H | -3.56079 | 0.960006 | 0.057855 |
| H | -1.38069 | -2.70104 | 0.386566 | H | -2.15071 | 0.398502 | -1.39455 |
| H | -0.55185 | -1.07273 | -2.07614 | H | -4.36073 | -2.25275 | -1.00048 |
| H | 0.148471 | -0.83841 | -0.48972 | H | -3.9751 | -2.63666 | 0.685659 |
| H | 1.504959 | -2.24917 | -2.12068 | H | -4.82699 | -1.13058 | 0.290762 |
| H | 0.248333 | -3.4643 | -2.07114 |  |  |  |  |

**Table S2:** (continued)

| **Conformer 8** | | | | | | | |
| --- | --- | --- | --- | --- | --- | --- | --- |
| Atom | X | Y | Z | Atom | X | Y | Z |
| C | 3.755907 | 0.116649 | 0.97002 | H | 0.479261 | 3.728034 | -0.10541 |
| C | 3.054916 | -0.54557 | -0.2419 | H | 0.145723 | 3.571084 | -1.82227 |
| C | 2.852346 | 0.41033 | -1.43319 | H | -1.98873 | 2.334165 | -1.27235 |
| C | 2.162787 | 1.769727 | -1.20484 | H | -1.96774 | 4.011209 | -0.75788 |
| C | 0.666357 | 1.707272 | -0.86751 | H | -1.3679 | 1.668115 | 1.124364 |
| C | -0.01186 | 3.084275 | -0.84994 | H | -1.45132 | 3.358387 | 1.568064 |
| C | -1.52209 | 3.026893 | -0.55897 | H | -3.86914 | 3.432669 | 0.978983 |
| C | -1.86874 | 2.611694 | 0.878527 | H | -3.5023 | 2.264454 | 2.23636 |
| C | -3.37382 | 2.46961 | 1.16475 | H | -5.15418 | 1.351294 | 0.702835 |
| C | -4.10819 | 1.375177 | 0.367531 | H | -4.14324 | 1.643935 | -0.69673 |
| C | -3.50412 | -0.02956 | 0.508859 | H | -3.35054 | -0.25978 | 1.572548 |
| C | -4.37717 | -1.12245 | -0.12299 | H | -2.50925 | -0.03873 | 0.048841 |
| C | -3.82669 | -2.55557 | -0.0223 | H | -5.36616 | -1.10007 | 0.354409 |
| C | -2.55365 | -2.82177 | -0.84333 | H | -4.54883 | -0.88142 | -1.18215 |
| C | -1.27022 | -2.34743 | -0.18641 | H | -4.60259 | -3.24708 | -0.36969 |
| O | -0.38535 | -1.87271 | -1.07024 | H | -3.62663 | -2.81031 | 1.025084 |
| C | 5.203385 | 0.544794 | 0.747752 | H | -2.43117 | -3.9046 | -0.97995 |
| O | 3.666729 | -0.85973 | 2.027893 | H | -2.62803 | -2.38936 | -1.84565 |
| O | 1.744248 | -0.93405 | 0.242307 | H | 0.438827 | -1.5843 | -0.58496 |
| C | 3.787915 | -1.81292 | -0.70886 | H | 5.28477 | 1.277609 | -0.06059 |
| O | -1.04472 | -2.42155 | 1.012086 | H | 5.594361 | 1.010706 | 1.657844 |
| H | 3.157285 | 0.992111 | 1.250432 | H | 5.837443 | -0.31271 | 0.507619 |
| H | 2.289225 | -0.14693 | -2.19339 | H | 3.730578 | -0.39428 | 2.872069 |
| H | 3.840736 | 0.590135 | -1.87119 | H | 1.893668 | -1.31417 | 1.125637 |
| H | 2.691132 | 2.343934 | -0.43245 | H | 3.189631 | -2.31865 | -1.47218 |
| H | 2.287814 | 2.346893 | -2.13129 | H | 4.76478 | -1.58033 | -1.14211 |
| H | 0.534867 | 1.206785 | 0.095185 | H | 3.937393 | -2.5011 | 0.127054 |
| H | 0.16045 | 1.069749 | -1.60673 |  |  |  |  |

**Table S2:** (continued)

| **Conformer 9** | | | | | | | |
| --- | --- | --- | --- | --- | --- | --- | --- |
| Atom | X | Y | Z | Atom | X | Y | Z |
| C | 2.095481 | -0.86436 | 1.137826 | H | -0.1986 | 4.126529 | -1.64747 |
| C | 2.741592 | -0.4211 | -0.22608 | H | 0.039511 | 2.410918 | -1.88304 |
| C | 2.107256 | 0.855385 | -0.80766 | H | -0.94445 | 3.778232 | 0.675819 |
| C | 2.193072 | 2.142354 | 0.024346 | H | -0.50023 | 2.083937 | 0.61317 |
| C | 1.576648 | 3.363972 | -0.68835 | H | -2.66085 | 3.358151 | -1.14206 |
| C | 0.109674 | 3.207858 | -1.13104 | H | -2.19417 | 1.671396 | -1.17057 |
| C | -0.88199 | 2.910621 | 0.003224 | H | -4.26881 | 1.982176 | 0.106422 |
| C | -2.28182 | 2.544242 | -0.5091 | H | -3.52494 | 3.183361 | 1.140813 |
| C | -3.32163 | 2.255598 | 0.588886 | H | -2.08872 | 1.536234 | 2.214227 |
| C | -2.93001 | 1.171268 | 1.61217 | H | -3.76389 | 1.041712 | 2.315623 |
| C | -2.54952 | -0.20305 | 1.033719 | H | -2.15792 | -0.82619 | 1.848104 |
| C | -3.7052 | -0.95267 | 0.355796 | H | -1.71969 | -0.07806 | 0.327196 |
| C | -3.37329 | -2.38188 | -0.10836 | H | -4.54494 | -1.01031 | 1.061702 |
| C | -2.37155 | -2.47439 | -1.27192 | H | -4.07269 | -0.37758 | -0.50488 |
| C | -0.91665 | -2.32168 | -0.87927 | H | -4.30219 | -2.86461 | -0.43159 |
| O | -0.19693 | -1.68727 | -1.7984 | H | -2.99068 | -2.97156 | 0.73281 |
| C | 2.783715 | -0.34143 | 2.394926 | H | -2.44319 | -3.46857 | -1.73338 |
| O | 2.103377 | -2.29981 | 1.208277 | H | -2.60414 | -1.75105 | -2.05887 |
| O | 2.396175 | -1.46181 | -1.19004 | H | 0.771662 | -1.62731 | -1.53384 |
| C | 4.270534 | -0.35321 | -0.17247 | H | 2.873212 | 0.747312 | 2.394164 |
| O | -0.4502 | -2.77573 | 0.163761 | H | 2.20471 | -0.63799 | 3.273829 |
| H | 1.052325 | -0.52132 | 1.129833 | H | 3.785673 | -0.76879 | 2.491324 |
| H | 1.054178 | 0.624083 | -1.00074 | H | 1.21911 | -2.59348 | 0.905671 |
| H | 2.566423 | 1.030086 | -1.7898 | H | 2.56665 | -2.28683 | -0.69509 |
| H | 1.691551 | 1.99923 | 0.987766 | H | 4.663554 | -0.18611 | -1.17918 |
| H | 3.23973 | 2.372752 | 0.257208 | H | 4.621652 | 0.45621 | 0.472569 |
| H | 2.18284 | 3.599936 | -1.57318 | H | 4.687781 | -1.29187 | 0.206685 |
| H | 1.662361 | 4.231014 | -0.02007 |  |  |  |  |

**Table S2:** (continued)

| **Conformer 10** | | | | | | | |
| --- | --- | --- | --- | --- | --- | --- | --- |
| Atom | X | Y | Z | Atom | X | Y | Z |
| C | 2.249296 | 0.371281 | -1.09207 | H | -0.55737 | 4.141821 | -0.95878 |
| C | 1.451809 | 1.356383 | -0.22403 | H | 0.625926 | 3.978217 | 0.326161 |
| C | 0.632409 | 2.340018 | -1.07477 | H | -1.50994 | 3.699241 | 1.363059 |
| C | -0.11236 | 3.417218 | -0.26296 | H | -0.78334 | 2.10915 | 1.325431 |
| C | -1.20668 | 2.892216 | 0.683049 | H | -2.15372 | 1.742914 | -0.89103 |
| C | -2.45657 | 2.339974 | -0.02285 | H | -3.051 | 3.173711 | -0.4231 |
| C | -3.31491 | 1.475124 | 0.910228 | H | -3.57923 | 2.062652 | 1.800589 |
| C | -4.61125 | 0.912553 | 0.301226 | H | -2.69417 | 0.645775 | 1.272738 |
| C | -4.45088 | 0.093646 | -0.99633 | H | -5.30552 | 1.738878 | 0.096477 |
| C | -3.36267 | -0.99254 | -0.97967 | H | -5.10342 | 0.293907 | 1.062809 |
| C | -3.51134 | -2.06931 | 0.103414 | H | -4.23143 | 0.77528 | -1.82796 |
| C | 3.101644 | -0.69834 | -0.37001 | H | -5.41957 | -0.36557 | -1.23606 |
| C | 4.21991 | -0.13968 | 0.55301 | H | -2.38347 | -0.51213 | -0.86393 |
| C | 5.127265 | 0.915219 | -0.07284 | H | -3.34175 | -1.48129 | -1.96315 |
| C | 3.698178 | -1.66154 | -1.4001 | H | -4.43561 | -2.63776 | -0.05636 |
| O | 2.264768 | -1.51136 | 0.490759 | H | -3.58962 | -1.60743 | 1.092596 |
| C | 3.559593 | 0.381771 | 1.723513 | H | 5.909715 | 1.198037 | 0.638245 |
| O | 4.826581 | -1.00499 | 0.852732 | H | 5.619493 | 0.535603 | -0.97319 |
| O | -2.32283 | -3.04325 | 0.117918 | H | 4.566074 | 1.815948 | -0.3345 |
| C | -1.02194 | -2.35567 | 0.498274 | H | 4.289177 | -2.43857 | -0.90708 |
| O | 0.011781 | -2.74019 | -0.25379 | H | 2.89593 | -2.14698 | -1.96357 |
| H | -0.92292 | -1.54112 | 1.405907 | H | 4.337434 | -1.13418 | -2.11282 |
| H | 1.546737 | -0.17333 | -1.73695 | H | 2.190562 | -1.01396 | 1.324752 |
| H | 2.906208 | 0.929786 | -1.77074 | H | 4.178099 | 0.331939 | 2.464204 |
| H | 2.127108 | 1.91794 | 0.430005 | H | -2.19826 | -3.53405 | -0.85162 |
| H | 0.787062 | 0.786716 | 0.435446 | H | -2.49385 | -3.83414 | 0.859412 |
| H | -0.07711 | 1.779308 | -1.69835 | H | 0.833836 | -2.25164 | 0.041966 |
| H | 1.308189 | 2.846594 | -1.77756 |  |  |  |  |

**Table S2:** (continued)

| **Conformer 11** | | | | | | | |
| --- | --- | --- | --- | --- | --- | --- | --- |
| Atom | X | Y | Z | Atom | X | Y | Z |
| C | -3.69525 | -0.51325 | -0.85331 | H | -1.13699 | 4.472029 | -0.72428 |
| C | -2.79867 | -0.58411 | 0.407847 | H | -0.10872 | 4.832027 | 0.651057 |
| C | -2.50549 | 0.794587 | 1.027656 | H | 1.201847 | 4.201543 | -1.3581 |
| C | -1.80752 | 1.807915 | 0.109119 | H | 0.379228 | 2.662009 | -1.46268 |
| C | -1.25771 | 3.020013 | 0.87481 | H | 2.319872 | 3.553553 | 0.730706 |
| C | -0.45931 | 4.013025 | 0.008393 | H | 1.340942 | 2.104788 | 0.87355 |
| C | 0.740745 | 3.413205 | -0.74832 | H | 3.334875 | 2.584853 | -1.39833 |
| C | 1.814766 | 2.773375 | 0.143407 | H | 2.304819 | 1.179219 | -1.20401 |
| C | 2.845285 | 1.95351 | -0.6434 | H | 4.607739 | 2.071727 | 0.601096 |
| C | 3.914718 | 1.298468 | 0.245102 | H | 3.434426 | 0.893395 | 1.145908 |
| C | 4.713911 | 0.182222 | -0.45412 | H | 5.61225 | -0.04613 | 0.135126 |
| C | 3.925464 | -1.12209 | -0.67926 | H | 5.074126 | 0.556395 | -1.4217 |
| C | 3.686347 | -1.93219 | 0.604271 | H | 2.962474 | -0.89023 | -1.15328 |
| C | 2.716231 | -3.11292 | 0.413396 | H | 4.465016 | -1.75297 | -1.39748 |
| C | 1.294682 | -2.68518 | 0.099881 | H | 3.291397 | -1.28749 | 1.395646 |
| O | 0.808601 | -1.8172 | 0.994154 | H | 4.644398 | -2.32252 | 0.968563 |
| C | -5.13704 | -0.07397 | -0.61585 | H | 3.046356 | -3.77267 | -0.39352 |
| O | -3.64955 | -1.84018 | -1.41591 | H | 2.681456 | -3.70729 | 1.334956 |
| O | -1.52746 | -1.10292 | -0.05636 | H | -0.10953 | -1.54982 | 0.704469 |
| C | -3.36491 | -1.54043 | 1.466468 | H | -5.18345 | 0.927246 | -0.177 |
| O | 0.647989 | -3.08893 | -0.85625 | H | -5.67572 | -0.04318 | -1.56825 |
| H | -3.21414 | 0.181609 | -1.55262 | H | -5.659 | -0.77061 | 0.045405 |
| H | -1.8649 | 0.613499 | 1.900997 | H | -3.86731 | -1.77535 | -2.35491 |
| H | -3.43985 | 1.210771 | 1.421939 | H | -1.73525 | -1.81774 | -0.68396 |
| H | -0.99098 | 1.291515 | -0.40458 | H | -2.62704 | -1.68139 | 2.261433 |
| H | -2.4945 | 2.159233 | -0.67222 | H | -4.27872 | -1.14399 | 1.918593 |
| H | -0.62771 | 2.662393 | 1.699765 | H | -3.59282 | -2.51479 | 1.026619 |
| H | -2.08878 | 3.559019 | 1.349353 |  |  |  |  |

**Table S2:** (continued)

| **Conformer 12** | | | | | | | |
| --- | --- | --- | --- | --- | --- | --- | --- |
| Atom | X | Y | Z | Atom | X | Y | Z |
| C | 2.277163 | -1.05991 | 1.160122 | H | 0.794614 | 4.523866 | 0.049944 |
| C | 2.65201 | -0.05868 | 0.015847 | H | -0.61821 | 4.618836 | -0.98139 |
| C | 1.702721 | 1.147993 | 0.037651 | H | -1.24232 | 4.025213 | 1.291551 |
| C | 1.948729 | 2.210593 | -1.05099 | H | -0.2107 | 2.614122 | 1.29829 |
| C | 0.703873 | 3.005847 | -1.48817 | H | -2.63899 | 3.008863 | -0.53142 |
| C | 0.025099 | 3.911284 | -0.44023 | H | -1.61667 | 1.583655 | -0.52105 |
| C | -0.83549 | 3.234638 | 0.645779 | H | -3.23141 | 2.595806 | 1.875604 |
| C | -2.00237 | 2.388164 | 0.116277 | H | -2.21879 | 1.167606 | 1.886808 |
| C | -2.85734 | 1.78284 | 1.238458 | H | -4.67045 | 0.680786 | 1.627652 |
| C | -4.06193 | 0.95544 | 0.75513 | H | -4.69973 | 1.596236 | 0.131066 |
| C | -3.73553 | -0.32661 | -0.03349 | H | -3.17253 | -0.0702 | -0.9393 |
| C | -2.97497 | -1.38978 | 0.774172 | H | -4.68174 | -0.76322 | -0.38178 |
| C | -2.85528 | -2.75659 | 0.078814 | H | -1.97226 | -1.02344 | 1.031827 |
| C | -2.04071 | -2.75807 | -1.23042 | H | -3.49149 | -1.54224 | 1.731417 |
| C | -0.58262 | -2.42365 | -0.9979 | H | -3.8584 | -3.13724 | -0.148 |
| O | -0.1548 | -1.38615 | -1.70771 | H | -2.39451 | -3.47378 | 0.766778 |
| C | 2.894347 | -0.74841 | 2.518966 | H | -2.06937 | -3.76405 | -1.66502 |
| O | 2.670244 | -2.38356 | 0.766752 | H | -2.45829 | -2.06901 | -1.96704 |
| O | 2.39454 | -0.76077 | -1.23657 | H | 0.811719 | -1.18485 | -1.51243 |
| C | 4.134149 | 0.326421 | 0.030035 | H | 2.671417 | 0.274635 | 2.836881 |
| O | 0.123516 | -3.05985 | -0.21738 | H | 2.490922 | -1.43337 | 3.269735 |
| H | 1.183899 | -1.03861 | 1.256657 | H | 3.980322 | -0.87389 | 2.494896 |
| H | 1.754545 | 1.603643 | 1.032205 | H | 1.856882 | -2.80362 | 0.422025 |
| H | 0.68655 | 0.750603 | -0.06739 | H | 2.81906 | -1.62866 | -1.09907 |
| H | 2.728428 | 2.90867 | -0.72022 | H | 4.387721 | 0.882377 | -0.87545 |
| H | 2.342912 | 1.710677 | -1.94349 | H | 4.377654 | 0.949617 | 0.894901 |
| H | -0.03212 | 2.307521 | -1.90744 | H | 4.762936 | -0.56928 | 0.066988 |
| H | 1.009176 | 3.64624 | -2.32579 |  |  |  |  |

**Table S2:** (continued)

| **Conformer 13** | | | | | | | |
| --- | --- | --- | --- | --- | --- | --- | --- |
| Atom | X | Y | Z | Atom | X | Y | Z |
| C | 2.756155 | -0.39715 | 0.878872 | H | -1.75766 | 4.014167 | -1.07877 |
| C | 3.184239 | 0.620894 | -0.22265 | H | -1.62016 | 2.289502 | -1.36288 |
| C | 2.370336 | 1.934192 | -0.16656 | H | -1.92298 | 3.698452 | 1.343237 |
| C | 0.845413 | 1.814219 | -0.31178 | H | -1.64544 | 1.977764 | 1.206416 |
| C | 0.159259 | 3.173317 | -0.52291 | H | -4.0503 | 2.606368 | 1.573854 |
| C | -1.37308 | 3.081924 | -0.64382 | H | -4.05931 | 3.474688 | 0.049318 |
| C | -2.09644 | 2.833139 | 0.689238 | H | -3.68864 | 1.339783 | -1.19832 |
| C | -3.61567 | 2.611839 | 0.564912 | H | -5.1467 | 1.316858 | -0.22842 |
| C | -4.0503 | 1.32442 | -0.16142 | H | -2.48487 | 0.04112 | 0.57832 |
| C | -3.57861 | 0.029726 | 0.514299 | H | -3.94352 | 0.004409 | 1.551673 |
| C | -4.02877 | -1.24352 | -0.21562 | H | -3.68922 | -1.19621 | -1.25921 |
| C | -3.54926 | -2.55553 | 0.435617 | H | -5.12561 | -1.26379 | -0.26392 |
| C | -2.0233 | -2.71009 | 0.561548 | H | -3.98086 | -2.6273 | 1.442605 |
| C | -1.30015 | -2.83171 | -0.8005 | H | -3.95428 | -3.40483 | -0.13048 |
| C | 0.14201 | -2.38704 | -0.74081 | H | -1.79359 | -3.59273 | 1.166668 |
| O | 0.84776 | -2.98893 | 0.212547 | H | -1.6188 | -1.85641 | 1.11576 |
| C | 3.265454 | -0.1208 | 2.285828 | H | -1.33312 | -3.87173 | -1.1467 |
| O | 3.199921 | -1.72091 | 0.478237 | H | -1.77851 | -2.21748 | -1.56528 |
| O | 2.996973 | -0.04086 | -1.49302 | H | 1.769408 | -2.59577 | 0.271052 |
| C | 4.683143 | 0.933276 | -0.16863 | H | 2.929474 | 0.859883 | 2.635718 |
| O | 0.602732 | -1.52866 | -1.49009 | H | 2.874804 | -0.8748 | 2.975339 |
| H | 1.660073 | -0.41916 | 0.903542 | H | 4.356967 | -0.15369 | 2.327617 |
| H | 2.755313 | 2.569107 | -0.97491 | H | 3.439798 | -1.61193 | -0.46427 |
| H | 2.606742 | 2.456119 | 0.769479 | H | 2.092616 | -0.41434 | -1.53272 |
| H | 0.600678 | 1.167391 | -1.16335 | H | 4.967701 | 1.48944 | -1.06601 |
| H | 0.42191 | 1.32737 | 0.574466 | H | 4.934158 | 1.539294 | 0.705113 |
| H | 0.565376 | 3.629673 | -1.43534 | H | 5.274609 | 0.013527 | -0.13467 |
| H | 0.422256 | 3.853466 | 0.299962 |  |  |  |  |

**Table S2:** (continued)

| **Conformer 14** | | | | | | | |
| --- | --- | --- | --- | --- | --- | --- | --- |
| Atom | X | Y | Z | Atom | X | Y | Z |
| C | 3.149837 | 0.831864 | -0.10382 | H | -0.36337 | -2.49368 | 0.903565 |
| C | 3.537009 | -0.63619 | 0.213933 | H | -0.92569 | -1.23207 | -0.17863 |
| C | 2.307853 | -1.58016 | 0.168059 | H | -1.81812 | -4.15446 | -0.39674 |
| C | 1.553898 | -1.6904 | -1.16628 | H | -2.46522 | -2.79943 | -1.29889 |
| C | 0.366713 | -2.67126 | -1.13295 | H | -3.97757 | -3.45813 | 0.544251 |
| C | -0.72746 | -2.30878 | -0.11612 | H | -2.68248 | -3.30281 | 1.718155 |
| C | -2.0375 | -3.08004 | -0.32686 | H | -2.60923 | -0.84255 | 1.372797 |
| C | -3.07967 | -2.87354 | 0.788026 | H | -4.16535 | -1.41159 | 1.937123 |
| C | -3.49236 | -1.41696 | 1.068462 | H | -3.54783 | -0.69482 | -0.97964 |
| C | -4.19981 | -0.70997 | -0.09719 | H | -5.07763 | -1.30447 | -0.38558 |
| C | -4.6651 | 0.722077 | 0.231233 | H | -5.2555 | 0.691808 | 1.156934 |
| C | -3.54513 | 1.767782 | 0.390123 | H | -5.35322 | 1.064281 | -0.55387 |
| C | -2.87288 | 2.160311 | -0.93377 | H | -3.9682 | 2.672365 | 0.846999 |
| C | -1.71263 | 3.151976 | -0.77659 | H | -2.78805 | 1.399177 | 1.092527 |
| C | -0.44928 | 2.623242 | -0.12785 | H | -3.62261 | 2.618586 | -1.59071 |
| O | -0.34681 | 1.291674 | -0.12681 | H | -2.50748 | 1.270399 | -1.45553 |
| C | 4.308041 | 1.821532 | -0.02535 | H | -1.40026 | 3.52179 | -1.76247 |
| O | 2.140541 | 1.183023 | 0.876949 | H | -2.01314 | 4.038872 | -0.20854 |
| O | 4.076362 | -0.68265 | 1.546531 | H | 0.530297 | 1.052134 | 0.282792 |
| C | 4.652772 | -1.12907 | -0.70929 | H | 5.031776 | 1.644702 | -0.8249 |
| O | 0.427977 | 3.353294 | 0.32383 | H | 3.937108 | 2.845431 | -0.14121 |
| H | 2.704299 | 0.874442 | -1.1065 | H | 4.818691 | 1.736785 | 0.937002 |
| H | 1.616186 | -1.25875 | 0.953958 | H | 1.975512 | 2.146089 | 0.841573 |
| H | 2.667756 | -2.57348 | 0.465211 | H | 3.427314 | -0.2277 | 2.10569 |
| H | 1.172757 | -0.70525 | -1.46464 | H | 4.849428 | -2.18832 | -0.51902 |
| H | 2.245587 | -2.0041 | -1.95771 | H | 4.381361 | -1.01604 | -1.76252 |
| H | 0.732206 | -3.6881 | -0.93165 | H | 5.577431 | -0.57626 | -0.52907 |
| H | -0.07713 | -2.70194 | -2.13737 |  |  |  |  |

**Table S3.** NMR calculation of **2b** (14*S*^*^, 15*R*^*^), (A) Boltzmann distribution of energy minimized conformers, (B) Optimized Z-matrixes of **2b** conformers in the MeOH (Ǻ)

(A)

| Conformer | Calculated Energy (G)  (atomic units) | Relative Energy (kcal/mol) | Boltzmann Weights (%) |
| --- | --- | --- | --- |
| 1 | -969.292112 | 0.000000 | 99.291103015 |
| 2 | -969.290980 | 0.710341 | 0.623889558 |
| 3 | -969.289627 | 1.559361 | 0.001456961 |
| 4 | -969.290495 | 1.014683 | 0.071081386 |
| 5 | -969.290106 | 1.258784 | 0.012448854 |
| 6 | -969.285143 | 4.373114 | 0.000000000 |
| 7 | -969.284234 | 4.943520 | 0.000000000 |
| 8 | -969.288421 | 2.316138 | 0.000006572 |
| 9 | -969.288117 | 2.506900 | 0.000001684 |
| 10 | -969.287309 | 3.013928 | 0.000000045 |
| 11 | -969.288554 | 2.232679 | 0.000011923 |
| 12 | -969.285959 | 3.861066 | 0.000000000 |
| 13 | -969.284862 | 4.549444 | 0.000000000 |

(B)

| **Conformer 1** | | | | | | | |
| --- | --- | --- | --- | --- | --- | --- | --- |
| Atom | X | Y | Z | Atom | X | Y | Z |
| C | -2.2499 | 1.110556 | -0.48307 | H | 0.207378 | 4.560766 | -0.07407 |
| C | -1.20909 | 2.106361 | 0.051358 | H | 1.168837 | 4.175429 | -1.49017 |
| C | -0.38673 | 2.742365 | -1.07808 | H | 1.348723 | 2.783372 | 1.238906 |
| C | 0.693864 | 3.730844 | -0.60489 | H | 2.468518 | 3.962466 | 0.590985 |
| C | 1.792463 | 3.146068 | 0.301986 | H | 3.045558 | 2.369781 | -1.2826 |
| C | 2.619259 | 2.017724 | -0.33133 | H | 1.960559 | 1.177921 | -0.58107 |
| C | 3.751812 | 1.523279 | 0.580183 | H | 3.319608 | 1.138961 | 1.513822 |
| C | 4.669449 | 0.458971 | -0.04919 | H | 4.373034 | 2.381445 | 0.87149 |
| C | 3.990742 | -0.85964 | -0.46301 | H | 5.479664 | 0.232693 | 0.6577 |
| C | 3.388118 | -1.65313 | 0.704783 | H | 5.15314 | 0.893844 | -0.93461 |
| C | 2.78811 | -3.01343 | 0.308026 | H | 4.734785 | -1.48725 | -0.97307 |
| C | -2.98397 | 0.275236 | 0.578315 | H | 3.216197 | -0.6466 | -1.20966 |
| C | -3.8599 | -0.8426 | -0.06268 | H | 2.625432 | -1.05434 | 1.209856 |
| C | -5.06363 | -0.33463 | -0.85299 | H | 4.177245 | -1.84194 | 1.445706 |
| C | -3.79297 | 1.128307 | 1.559467 | H | 2.470037 | -3.54567 | 1.212967 |
| O | -1.93082 | -0.39944 | 1.335332 | H | 3.576711 | -3.6291 | -0.14276 |
| O | -3.10978 | -1.66364 | -0.94572 | H | -5.61554 | -1.19061 | -1.25065 |
| H | -4.23863 | -1.44685 | 0.782232 | H | -5.7434 | 0.248237 | -0.22734 |
| C | 1.61272 | -2.99934 | -0.69773 | H | -4.74826 | 0.28365 | -1.69859 |
| C | 0.296534 | -2.46639 | -0.17633 | H | -4.36247 | 0.496964 | 2.251466 |
| O | 0.369297 | -1.23045 | 0.313721 | H | -3.12481 | 1.759081 | 2.15033 |
| O | -0.75289 | -3.10286 | -0.21718 | H | -4.49995 | 1.776306 | 1.035073 |
| H | -3.00109 | 1.648865 | -1.07247 | H | -2.33427 | -0.94728 | 2.024753 |
| H | -1.76455 | 0.408891 | -1.17025 | H | -2.38092 | -2.09521 | -0.45996 |
| H | -0.54184 | 1.588936 | 0.747926 | H | 1.417392 | -4.01458 | -1.04977 |
| H | -1.70168 | 2.899745 | 0.628526 | H | 1.879843 | -2.39942 | -1.57605 |
| H | -1.06515 | 3.269929 | -1.7624 | H | -0.52499 | -0.95636 | 0.670237 |
| H | 0.07698 | 1.945727 | -1.67464 |  |  |  |  |

**Table S3:** (continued)

| **Conformer 2** | | | | | | | |
| --- | --- | --- | --- | --- | --- | --- | --- |
| Atom | X | Y | Z | Atom | X | Y | Z |
| C | 1.928531 | -1.27805 | -0.34103 | H | -1.65342 | -2.83664 | 1.089263 |
| C | 0.855228 | -2.14125 | 0.338557 | H | -1.06843 | -4.25802 | 0.253496 |
| C | -0.351 | -2.39638 | -0.57319 | H | -3.43508 | -4.03608 | -0.21917 |
| C | -1.44849 | -3.24094 | 0.089221 | H | -2.54432 | -3.74844 | -1.70354 |
| C | -2.76061 | -3.32371 | -0.71376 | H | -4.31402 | -2.11826 | -1.6139 |
| C | -3.49325 | -1.98175 | -0.89778 | H | -2.81232 | -1.26342 | -1.36891 |
| C | -4.06561 | -1.38123 | 0.399835 | H | -3.3785 | -1.56187 | 1.236491 |
| C | -4.33682 | 0.131422 | 0.322435 | H | -4.99236 | -1.91116 | 0.655218 |
| C | -3.06292 | 0.987379 | 0.402628 | H | -5.00361 | 0.422302 | 1.145224 |
| C | -3.34169 | 2.492096 | 0.316436 | H | -4.88066 | 0.362631 | -0.60496 |
| C | -2.11433 | 3.400658 | 0.49721 | H | -2.36862 | 0.695885 | -0.39605 |
| C | 3.022969 | -0.72833 | 0.597995 | H | -2.54539 | 0.769004 | 1.346588 |
| C | 3.991931 | 0.256405 | -0.13474 | H | -4.08018 | 2.759737 | 1.08425 |
| C | 4.469322 | -0.15356 | -1.51958 | H | -3.81549 | 2.718453 | -0.64964 |
| C | 3.840426 | -1.84866 | 1.246645 | H | -1.64936 | 3.205141 | 1.469836 |
| O | 2.432658 | 0.010699 | 1.685592 | H | -2.46209 | 4.439062 | 0.522185 |
| O | 3.373159 | 1.557672 | -0.2631 | H | 5.205728 | 0.566797 | -1.88612 |
| H | 4.864425 | 0.383644 | 0.520584 | H | 4.942557 | -1.13875 | -1.4924 |
| C | -1.04096 | 3.284369 | -0.59857 | H | 3.641881 | -0.18749 | -2.23361 |
| C | 0.150626 | 2.406366 | -0.27399 | H | 4.573917 | -1.4267 | 1.940551 |
| O | 1.023881 | 2.365371 | -1.28198 | H | 3.191561 | -2.52199 | 1.811055 |
| O | 0.326868 | 1.825153 | 0.792121 | H | 4.375154 | -2.43675 | 0.494979 |
| H | 2.403176 | -1.85635 | -1.14175 | H | 1.676423 | 0.524082 | 1.340596 |
| H | 1.437682 | -0.42751 | -0.83245 | H | 3.173868 | 1.829126 | 0.647932 |
| H | 0.513407 | -1.64001 | 1.252135 | H | -0.61643 | 4.268685 | -0.83046 |
| H | 1.286365 | -3.09949 | 0.654746 | H | -1.46323 | 2.929509 | -1.54706 |
| H | -0.02247 | -2.88595 | -1.50122 | H | 1.867412 | 1.924582 | -0.98112 |
| H | -0.76023 | -1.4239 | -0.87382 |  |  |  |  |

**Table S3:** (continued)

| **Conformer 3** | | | | | | | |
| --- | --- | --- | --- | --- | --- | --- | --- |
| Atom | X | Y | Z | Atom | X | Y | Z |
| C | 1.904569 | 1.109986 | 0.055815 | H | 0.244801 | 3.965159 | 0.567235 |
| C | 1.489988 | 2.085576 | -1.06228 | H | -0.32519 | 2.4063 | 1.125669 |
| C | 0.011075 | 2.503605 | -1.00823 | H | -1.9239 | 4.550458 | -0.36244 |
| C | -0.43456 | 3.138537 | 0.316174 | H | -2.11183 | 4.060223 | 1.311969 |
| C | -1.87629 | 3.681363 | 0.308002 | H | -2.82743 | 2.422444 | -1.1791 |
| C | -2.96781 | 2.68432 | -0.12187 | H | -3.9405 | 3.192386 | -0.06899 |
| C | -3.03406 | 1.390128 | 0.700331 | H | -3.17092 | 1.632384 | 1.764341 |
| C | -4.16261 | 0.455605 | 0.240744 | H | -2.07301 | 0.867093 | 0.629222 |
| C | -4.21789 | -0.89915 | 0.969373 | H | -4.07251 | 0.290165 | -0.8413 |
| C | -3.0122 | -1.83043 | 0.747044 | H | -5.12487 | 0.965849 | 0.382367 |
| C | -2.83696 | -2.30193 | -0.70368 | H | -5.12989 | -1.42805 | 0.66064 |
| C | 3.083681 | 0.18249 | -0.31678 | H | -4.32373 | -0.71776 | 2.047451 |
| C | 3.488606 | -0.74714 | 0.860273 | H | -3.12961 | -2.71204 | 1.391064 |
| C | 4.110079 | -0.06498 | 2.076923 | H | -2.09373 | -1.3338 | 1.086222 |
| C | 4.316575 | 0.966622 | -0.78401 | H | -2.70342 | -1.44623 | -1.37367 |
| O | 2.718117 | -0.64985 | -1.42644 | H | -3.7455 | -2.82122 | -1.03143 |
| O | 2.306015 | -1.47226 | 1.281357 | H | 4.3475 | -0.81095 | 2.844013 |
| H | 4.206062 | -1.46616 | 0.441311 | H | 5.044839 | 0.436278 | 1.815663 |
| C | -1.63904 | -3.25262 | -0.89701 | H | 3.430035 | 0.668895 | 2.51824 |
| C | -0.32221 | -2.54036 | -0.6761 | H | 5.159174 | 0.290137 | -0.96223 |
| O | 0.427909 | -3.07584 | 0.280581 | H | 4.091031 | 1.474084 | -1.72422 |
| O | 0.012881 | -1.54754 | -1.31828 | H | 4.62379 | 1.718275 | -0.05252 |
| H | 2.150915 | 1.667143 | 0.966366 | H | 1.792947 | -0.94249 | -1.30956 |
| H | 1.055686 | 0.466631 | 0.31221 | H | 2.534701 | -1.97591 | 2.075377 |
| H | 1.672834 | 1.606972 | -2.03102 | H | -1.63335 | -3.62645 | -1.9271 |
| H | 2.125606 | 2.980077 | -1.03061 | H | -1.706 | -4.11288 | -0.22615 |
| H | -0.60205 | 1.615951 | -1.21122 | H | 1.220442 | -2.49467 | 0.45602 |
| H | -0.18806 | 3.205946 | -1.82989 |  |  |  |  |

**Table S3:** (continued)

| **Conformer 4** | | | | | | | |
| --- | --- | --- | --- | --- | --- | --- | --- |
| Atom | X | Y | Z | Atom | X | Y | Z |
| C | -1.3778 | -0.74946 | -0.4813 | H | -0.04436 | -3.92902 | 0.868885 |
| C | -1.40145 | -2.21781 | -0.94124 | H | 0.574767 | -2.30045 | 1.021001 |
| C | -1.6E-05 | -2.85604 | -0.99761 | H | 2.042791 | -4.67855 | -0.23912 |
| C | 0.60017 | -3.18783 | 0.377228 | H | 2.329751 | -4.01365 | 1.359355 |
| C | 2.036731 | -3.74256 | 0.336013 | H | 2.91061 | -2.63545 | -1.31854 |
| C | 3.102943 | -2.79661 | -0.24933 | H | 4.07956 | -3.2966 | -0.19491 |
| C | 3.186833 | -1.43447 | 0.449994 | H | 3.367478 | -1.58196 | 1.524573 |
| C | 4.267388 | -0.50109 | -0.11797 | H | 2.212124 | -0.93935 | 0.376242 |
| C | 4.142033 | 0.949548 | 0.384701 | H | 4.214064 | -0.50439 | -1.21631 |
| C | 2.96296 | 1.704487 | -0.24696 | H | 5.258273 | -0.89765 | 0.138434 |
| C | 2.648018 | 3.051108 | 0.415432 | H | 5.071478 | 1.495838 | 0.178271 |
| C | -2.74058 | -0.03454 | -0.40126 | H | 4.028913 | 0.94209 | 1.478255 |
| C | -3.73127 | -0.66977 | 0.618331 | H | 2.06446 | 1.07835 | -0.19546 |
| C | -3.14447 | -0.92579 | 2.009437 | H | 3.166224 | 1.86023 | -1.31571 |
| C | -3.40467 | 0.091558 | -1.774 | H | 3.509656 | 3.724222 | 0.334184 |
| O | -2.52556 | 1.340115 | 0.041412 | H | 2.463361 | 2.899039 | 1.484897 |
| O | -4.88758 | 0.165313 | 0.722829 | H | -3.93981 | -1.28237 | 2.669298 |
| H | -4.08785 | -1.61905 | 0.202851 | H | -2.35232 | -1.67965 | 1.994549 |
| C | 1.425053 | 3.745244 | -0.20231 | H | -2.73787 | -0.00646 | 2.445611 |
| C | 0.143902 | 2.963132 | 0.001151 | H | -4.31978 | 0.681235 | -1.69427 |
| O | -0.69989 | 3.050528 | -1.03077 | H | -2.72768 | 0.57909 | -2.48173 |
| O | -0.10755 | 2.317637 | 1.01496 | H | -3.66916 | -0.89191 | -2.17098 |
| H | -0.89025 | -0.67765 | 0.49794 | H | -1.82395 | 1.369665 | 0.723116 |
| H | -0.75183 | -0.17452 | -1.17598 | H | -4.53341 | 1.063926 | 0.824949 |
| H | -1.85262 | -2.27386 | -1.93836 | H | 1.561738 | 3.922573 | -1.27299 |
| H | -2.04208 | -2.81641 | -0.28038 | H | 1.271272 | 4.727479 | 0.263274 |
| H | 0.668498 | -2.18347 | -1.55081 | H | -1.49798 | 2.495873 | -0.82207 |
| H | -0.05416 | -3.78056 | -1.58744 |  |  |  |  |

**Table S3:** (continued)

| **Conformer 5** | | | | | | | |
| --- | --- | --- | --- | --- | --- | --- | --- |
| Atom | X | Y | Z | Atom | X | Y | Z |
| C | -1.70501 | -1.36618 | 0.381533 | H | 1.793282 | -2.6591 | -1.42645 |
| C | -0.69556 | -2.15926 | -0.4622 | H | 1.278088 | -4.21534 | -0.81424 |
| C | 0.548228 | -2.55515 | 0.345164 | H | 3.48812 | -4.31451 | -0.12744 |
| C | 1.644449 | -3.23003 | -0.49863 | H | 2.837005 | -3.52575 | 1.294562 |
| C | 3.006549 | -3.3911 | 0.216878 | H | 4.254469 | -2.19099 | -1.07605 |
| C | 3.997901 | -2.23121 | -0.00749 | H | 4.934456 | -2.45448 | 0.521948 |
| C | 3.474563 | -0.85832 | 0.427216 | H | 3.221897 | -0.885 | 1.497373 |
| C | 4.424353 | 0.317744 | 0.167741 | H | 2.536962 | -0.66747 | -0.10201 |
| C | 3.816048 | 1.676061 | 0.565548 | H | 4.698223 | 0.33894 | -0.89691 |
| C | 2.582866 | 2.070602 | -0.26053 | H | 5.360127 | 0.165512 | 0.721805 |
| C | 1.900869 | 3.355919 | 0.220252 | H | 4.578523 | 2.460157 | 0.468771 |
| C | -3.00384 | -0.94924 | -0.34168 | H | 3.542418 | 1.645978 | 1.629994 |
| C | -3.87262 | 0.027027 | 0.520194 | H | 1.848321 | 1.257495 | -0.23663 |
| C | -4.00169 | -0.29815 | 2.000663 | H | 2.872008 | 2.184251 | -1.31479 |
| C | -3.8581 | -2.16018 | -0.72869 | H | 2.59569 | 4.200797 | 0.145854 |
| O | -2.70936 | -0.26862 | -1.57523 | H | 1.633395 | 3.263083 | 1.279662 |
| O | -3.35955 | 1.375204 | 0.422439 | H | -4.69881 | 0.399045 | 2.473472 |
| H | -4.87405 | 0.036405 | 0.068578 | H | -4.38492 | -1.31179 | 2.145254 |
| C | 0.634348 | 3.707301 | -0.59036 | H | -3.03891 | -0.21652 | 2.512517 |
| C | -0.46522 | 2.67644 | -0.45264 | H | -4.74152 | -1.83327 | -1.2856 |
| O | -1.02078 | 2.663141 | 0.759735 | H | -3.29486 | -2.84604 | -1.36521 |
| O | -0.8131 | 1.924611 | -1.35921 | H | -4.19205 | -2.70955 | 0.156367 |
| H | -1.97355 | -1.95523 | 1.266462 | H | -1.9794 | 0.361998 | -1.42271 |
| H | -1.20904 | -0.46118 | 0.759172 | H | -3.3881 | 1.588042 | -0.52448 |
| H | -0.39225 | -1.55501 | -1.32588 | H | 0.873304 | 3.791359 | -1.65341 |
| H | -1.16483 | -3.06284 | -0.87078 | H | 0.237033 | 4.668752 | -0.24829 |
| H | 0.256882 | -3.23222 | 1.160558 | H | -1.80373 | 2.046404 | 0.755325 |
| H | 0.949191 | -1.66042 | 0.833492 |  |  |  |  |

**Table S3:** (continued)

| **Conformer 6** | | | | | | | |
| --- | --- | --- | --- | --- | --- | --- | --- |
| Atom | X | Y | Z | Atom | X | Y | Z |
| C | 2.995138 | 1.034139 | -0.1236 | H | 0.549944 | 4.403175 | -0.10795 |
| C | 1.755483 | 1.213703 | -1.01384 | H | 1.75263 | 3.507849 | 0.79436 |
| C | 1.301559 | 2.67778 | -1.15625 | H | -0.37608 | 3.242724 | 1.893433 |
| C | 0.875884 | 3.385105 | 0.145526 | H | 0.104726 | 1.688221 | 1.252081 |
| C | -0.2323 | 2.69058 | 0.954801 | H | -1.45254 | 2.070992 | -0.72524 |
| C | -1.58801 | 2.564071 | 0.24505 | H | -1.98205 | 3.56701 | 0.025177 |
| C | -2.60597 | 1.777295 | 1.082654 | H | -2.69857 | 2.254965 | 2.067777 |
| C | -4.01391 | 1.654879 | 0.474536 | H | -2.20043 | 0.775186 | 1.276914 |
| C | -4.09591 | 0.987695 | -0.91307 | H | -4.46074 | 2.655465 | 0.398447 |
| C | -3.39351 | -0.37376 | -1.04318 | H | -4.64522 | 1.101489 | 1.18148 |
| C | -3.9103 | -1.46597 | -0.09655 | H | -3.66971 | 1.662807 | -1.66579 |
| C | 3.641016 | -0.37055 | -0.07929 | H | -5.15564 | 0.87498 | -1.17932 |
| C | 2.727098 | -1.50812 | 0.457699 | H | -2.31628 | -0.23738 | -0.88409 |
| C | 2.049068 | -1.23876 | 1.79176 | H | -3.49692 | -0.72521 | -2.07844 |
| C | 4.942073 | -0.31659 | 0.733644 | H | -4.96572 | -1.67452 | -0.3095 |
| O | 3.94456 | -0.83135 | -1.4182 | H | -3.85913 | -1.12619 | 0.943328 |
| O | 1.710023 | -1.80596 | -0.51165 | H | 1.406341 | -2.08261 | 2.053175 |
| H | 3.371203 | -2.39521 | 0.558542 | H | 2.789608 | -1.10873 | 2.585127 |
| C | -3.11274 | -2.78198 | -0.20851 | H | 1.419582 | -0.34637 | 1.749444 |
| C | -1.67011 | -2.56637 | 0.208093 | H | 5.423881 | -1.29833 | 0.75481 |
| O | -0.80494 | -2.72721 | -0.7985 | H | 5.63855 | 0.395051 | 0.278729 |
| O | -1.33643 | -2.24181 | 1.337961 | H | 4.762923 | 0.006292 | 1.763467 |
| H | 3.775145 | 1.72982 | -0.46843 | H | 4.357146 | -0.10532 | -1.9061 |
| H | 2.77306 | 1.337999 | 0.905307 | H | 2.145561 | -1.72453 | -1.377 |
| H | 0.932603 | 0.599151 | -0.63781 | H | -3.14873 | -3.17436 | -1.22827 |
| H | 1.981199 | 0.825883 | -2.01442 | H | -3.54498 | -3.53143 | 0.462868 |
| H | 0.472723 | 2.709741 | -1.8745 | H | 0.10936 | -2.46746 | -0.50863 |
| H | 2.113481 | 3.260652 | -1.61279 |  |  |  |  |

**Table S3:** (continued)

| **Conformer 7** | | | | | | | |
| --- | --- | --- | --- | --- | --- | --- | --- |
| Atom | X | Y | Z | Atom | X | Y | Z |
| C | 2.292381 | 1.956405 | -0.02671 | H | 4.014168 | -0.13692 | 1.214107 |
| C | 2.338735 | 0.763574 | -0.99686 | H | 4.815421 | -1.39071 | 0.291647 |
| C | 3.631722 | -0.06883 | -0.92111 | H | 1.838601 | -1.29042 | 1.008548 |
| C | 3.86686 | -0.84517 | 0.388647 | H | 3.055733 | -2.31155 | 1.739083 |
| C | 2.7627 | -1.83986 | 0.790867 | H | 3.423509 | -3.41899 | -0.52359 |
| C | 2.475279 | -2.94061 | -0.24146 | H | 2.082612 | -2.49358 | -1.16307 |
| C | 1.51215 | -4.03627 | 0.254735 | H | 1.972741 | -4.5403 | 1.115393 |
| C | 0.106634 | -3.56235 | 0.666876 | H | 1.415244 | -4.80127 | -0.52784 |
| C | -0.74806 | -2.98958 | -0.47284 | H | 0.191973 | -2.81092 | 1.462925 |
| C | -2.0766 | -2.40157 | 0.020458 | H | -0.42865 | -4.40959 | 1.116196 |
| C | -2.95484 | -1.83742 | -1.10513 | H | -0.93934 | -3.77559 | -1.21727 |
| C | 1.016883 | 2.82916 | -0.08664 | H | -0.19301 | -2.20183 | -0.99942 |
| C | -0.259 | 2.034317 | 0.284305 | H | -1.85804 | -1.60967 | 0.748845 |
| C | -0.22442 | 1.385562 | 1.668533 | H | -2.64137 | -3.1671 | 0.570598 |
| C | 0.857108 | 3.503018 | -1.45649 | H | -3.32925 | -2.65779 | -1.72861 |
| O | 1.092231 | 3.847458 | 0.935192 | H | -2.35352 | -1.19675 | -1.76139 |
| O | -1.41039 | 2.897596 | 0.181232 | H | -1.20231 | 0.949445 | 1.886317 |
| H | -0.42742 | 1.271036 | -0.48009 | H | 0.52618 | 0.592786 | 1.725739 |
| C | -4.16546 | -1.01914 | -0.5975 | H | 0.008549 | 2.130429 | 2.434871 |
| C | -3.71394 | 0.234095 | 0.122519 | H | 0.000627 | 4.17907 | -1.44603 |
| O | -3.23827 | 1.157432 | -0.72697 | H | 1.757535 | 4.077585 | -1.70234 |
| O | -3.72745 | 0.383581 | 1.334002 | H | 0.701901 | 2.769774 | -2.25298 |
| H | 2.420634 | 1.615345 | 1.005494 | H | 1.74955 | 4.499736 | 0.656307 |
| H | 3.146798 | 2.615425 | -0.23625 | H | -1.26743 | 3.632442 | 0.797162 |
| H | 2.234981 | 1.130324 | -2.02457 | H | -4.76755 | -1.61064 | 0.097024 |
| H | 1.477496 | 0.106127 | -0.82606 | H | -4.79367 | -0.7323 | -1.44691 |
| H | 4.491158 | 0.594022 | -1.09033 | H | -2.79262 | 1.88719 | -0.22418 |
| H | 3.631028 | -0.77535 | -1.76022 |  |  |  |  |

**Table S3:** (continued)

| **Conformer 8** | | | | | | | |
| --- | --- | --- | --- | --- | --- | --- | --- |
| Atom | X | Y | Z | Atom | X | Y | Z |
| C | -1.3235 | -0.92315 | -0.21362 | H | 1.336509 | -1.37855 | -1.05852 |
| C | -1.19745 | -2.44348 | -0.4136 | H | 1.011554 | -2.73682 | -2.11192 |
| C | 0.215811 | -2.98199 | -0.12344 | H | 3.385829 | -2.63709 | -1.58812 |
| C | 1.30016 | -2.47404 | -1.08484 | H | 2.699683 | -4.11963 | -0.95898 |
| C | 2.7104 | -3.03021 | -0.8178 | H | 4.324254 | -3.11463 | 0.610526 |
| C | 3.294584 | -2.7328 | 0.578338 | H | 2.740963 | -3.30957 | 1.329889 |
| C | 3.288424 | -1.2552 | 1.004981 | H | 3.614921 | -1.192 | 2.051751 |
| C | 4.178021 | -0.3233 | 0.166194 | H | 2.256048 | -0.88422 | 0.997619 |
| C | 3.960065 | 1.167298 | 0.487352 | H | 3.997466 | -0.48427 | -0.90503 |
| C | 2.688362 | 1.744382 | -0.15318 | H | 5.228888 | -0.59005 | 0.338744 |
| C | 2.307787 | 3.133731 | 0.372053 | H | 4.823896 | 1.752415 | 0.14659 |
| C | -2.73855 | -0.3253 | -0.34548 | H | 3.915357 | 1.297811 | 1.578132 |
| C | -3.74828 | -0.83894 | 0.717552 | H | 1.847433 | 1.06168 | 0.02299 |
| C | -3.24273 | -0.77635 | 2.161499 | H | 2.825964 | 1.786702 | -1.24266 |
| C | -3.30945 | -0.51744 | -1.75518 | H | 3.161729 | 3.815669 | 0.278435 |
| O | -2.65737 | 1.106812 | -0.07809 | H | 2.074626 | 3.068906 | 1.440804 |
| O | -4.97089 | -0.10626 | 0.593226 | H | -4.05558 | -1.06253 | 2.834456 |
| H | -4.000003 | -1.87655 | 0.471946 | H | -2.4041 | -1.45676 | 2.335315 |
| C | 1.117475 | 3.767158 | -0.37176 | H | -2.92423 | 0.237816 | 2.42267 |
| C | -0.14146 | 2.929081 | -0.36968 | H | -4.28216 | -0.02898 | -1.83656 |
| O | -0.47964 | 2.473099 | 0.840789 | H | -2.63292 | -0.0927 | -2.50493 |
| O | -0.80294 | 2.695212 | -1.37754 | H | -3.44032 | -1.57858 | -1.98461 |
| H | -0.92709 | -0.65611 | 0.773189 | H | -2.28254 | 1.562476 | -0.86 |
| H | -0.69332 | -0.4047 | -0.946 | H | -4.6955 | 0.823118 | 0.536311 |
| H | -1.48084 | -2.71 | -1.43966 | H | 1.36567 | 3.962948 | -1.41791 |
| H | -1.90488 | -2.9628 | 0.244616 | H | 0.86555 | 4.729736 | 0.091091 |
| H | 0.189528 | -4.0792 | -0.16755 | H | -1.29593 | 1.91549 | 0.743316 |
| H | 0.482259 | -2.72665 | 0.910596 |  |  |  |  |

**Table S3:** (continued)

| **Conformer 9** | | | | | | | |
| --- | --- | --- | --- | --- | --- | --- | --- |
| Atom | X | Y | Z | Atom | X | Y | Z |
| C | -2.53651 | -1.35326 | 0.652484 | H | 1.195796 | -1.81276 | -1.28981 |
| C | -1.34507 | -1.50344 | -0.3068 | H | 0.457589 | -3.3985 | -1.36598 |
| C | -0.166 | -2.24603 | 0.338842 | H | 2.676543 | -3.92724 | -0.76936 |
| C | 0.907309 | -2.67492 | -0.67311 | H | 1.840842 | -3.98079 | 0.770277 |
| C | 2.161271 | -3.29979 | -0.02965 | H | 3.845659 | -2.79175 | 1.237499 |
| C | 3.171893 | -2.28243 | 0.535185 | H | 2.637726 | -1.52703 | 1.125739 |
| C | 4.019175 | -1.6043 | -0.55245 | H | 3.361676 | -1.15052 | -1.30427 |
| C | 5.01495 | -0.54694 | -0.04292 | H | 4.58393 | -2.38173 | -1.08508 |
| C | 4.395089 | 0.67604 | 0.657675 | H | 5.61183 | -0.19724 | -0.89642 |
| C | 3.356415 | 1.425909 | -0.18508 | H | 5.724066 | -1.025 | 0.646795 |
| C | 2.813144 | 2.689905 | 0.494895 | H | 5.204676 | 1.364927 | 0.935118 |
| C | -3.80887 | -0.65392 | 0.111988 | H | 3.927989 | 0.368183 | 1.602658 |
| C | -3.70944 | 0.885431 | -0.06313 | H | 2.515942 | 0.755522 | -0.39781 |
| C | -3.2949 | 1.676277 | 1.168021 | H | 3.791831 | 1.694232 | -1.15842 |
| C | -5.00588 | -0.99418 | 1.01222 | H | 3.604679 | 3.445503 | 0.564212 |
| O | -4.0988 | -1.0878 | -1.23853 | H | 2.510651 | 2.452278 | 1.521031 |
| O | -2.79735 | 1.213178 | -1.12704 | H | -3.33616 | 2.746203 | 0.942426 |
| H | -4.71303 | 1.211048 | -0.37591 | H | -3.97323 | 1.485564 | 2.003269 |
| C | 1.609366 | 3.291527 | -0.23821 | H | -2.27298 | 1.433951 | 1.467642 |
| C | 0.365845 | 2.425376 | -0.13672 | H | -5.9084 | -0.47533 | 0.675683 |
| O | -0.51399 | 2.673053 | -1.11202 | H | -5.1998 | -2.07102 | 0.978427 |
| O | 0.175908 | 1.606734 | 0.750487 | H | -4.81664 | -0.7248 | 2.055518 |
| H | -2.83679 | -2.35964 | 0.977891 | H | -4.02631 | -2.05164 | -1.27179 |
| H | -2.21701 | -0.83649 | 1.565107 | H | -2.92704 | 0.530956 | -1.80633 |
| H | -1.00753 | -0.52083 | -0.64653 | H | 1.826659 | 3.47085 | -1.29654 |
| H | -1.66962 | -2.05098 | -1.20116 | H | 1.345687 | 4.268873 | 0.187789 |
| H | -0.53348 | -3.14248 | 0.85927 | H | -1.31612 | 2.093721 | -1.00579 |
| H | 0.277505 | -1.60501 | 1.112149 |  |  |  |  |

**Table S3:** (continued)

| **Conformer 10** | | | | | | | |
| --- | --- | --- | --- | --- | --- | --- | --- |
| Atom | X | Y | Z | Atom | X | Y | Z |
| C | 2.249296 | 0.371281 | -1.09207 | H | -0.55737 | 4.141821 | -0.95878 |
| C | 1.451809 | 1.356383 | -0.22403 | H | 0.625926 | 3.978217 | 0.326161 |
| C | 0.632409 | 2.340018 | -1.07477 | H | -1.50994 | 3.699241 | 1.363059 |
| C | -0.11236 | 3.417218 | -0.26296 | H | -0.78334 | 2.10915 | 1.325431 |
| C | -1.20668 | 2.892216 | 0.683049 | H | -2.15372 | 1.742914 | -0.89103 |
| C | -2.45657 | 2.339974 | -0.02285 | H | -3.051 | 3.173711 | -0.4231 |
| C | -3.31491 | 1.475124 | 0.910228 | H | -3.57923 | 2.062652 | 1.800589 |
| C | -4.61125 | 0.912553 | 0.301226 | H | -2.69417 | 0.645775 | 1.272738 |
| C | -4.45088 | 0.093646 | -0.99633 | H | -5.30552 | 1.738878 | 0.096477 |
| C | -3.36267 | -0.99254 | -0.97967 | H | -5.10342 | 0.293907 | 1.062809 |
| C | -3.51134 | -2.06931 | 0.103414 | H | -4.23143 | 0.77528 | -1.82796 |
| C | 3.101644 | -0.69834 | -0.37001 | H | -5.41957 | -0.36557 | -1.23606 |
| C | 4.21991 | -0.13968 | 0.55301 | H | -2.38347 | -0.51213 | -0.86393 |
| C | 5.127265 | 0.915219 | -0.07284 | H | -3.34175 | -1.48129 | -1.96315 |
| C | 3.698178 | -1.66154 | -1.4001 | H | -4.43561 | -2.63776 | -0.05636 |
| O | 2.264768 | -1.51136 | 0.490759 | H | -3.58962 | -1.60743 | 1.092596 |
| O | 3.559593 | 0.381771 | 1.723513 | H | 5.909715 | 1.198037 | 0.638245 |
| H | 4.826581 | -1.00499 | 0.852732 | H | 5.619493 | 0.535603 | -0.97319 |
| C | -2.32283 | -3.04325 | 0.117918 | H | 4.566074 | 1.815948 | -0.3345 |
| C | -1.02194 | -2.35567 | 0.498274 | H | 4.289177 | -2.43857 | -0.90708 |
| O | 0.011781 | -2.74019 | -0.25379 | H | 2.89593 | -2.14698 | -1.96357 |
| O | -0.92292 | -1.54112 | 1.405907 | H | 4.337434 | -1.13418 | -2.11282 |
| H | 1.546737 | -0.17333 | -1.73695 | H | 2.190562 | -1.01396 | 1.324752 |
| H | 2.906208 | 0.929786 | -1.77074 | H | 4.178099 | 0.331939 | 2.464204 |
| H | 2.127108 | 1.91794 | 0.430005 | H | -2.19826 | -3.53405 | -0.85162 |
| H | 0.787062 | 0.786716 | 0.435446 | H | -2.49385 | -3.83414 | 0.859412 |
| H | -0.07711 | 1.779308 | -1.69835 | H | 0.833836 | -2.25164 | 0.041966 |
| H | 1.308189 | 2.846594 | -1.77756 |  |  |  |  |

**Table S3:** (continued)

| **Conformer 11** | | | | | | | |
| --- | --- | --- | --- | --- | --- | --- | --- |
| Atom | X | Y | Z | Atom | X | Y | Z |
| C | 1.929087 | 0.802315 | 0.311499 | H | -0.65426 | 1.590467 | -0.34363 |
| C | 2.007062 | 2.21425 | -0.2937 | H | -0.46427 | 2.799592 | -1.59162 |
| C | 0.815051 | 3.098298 | 0.125598 | H | -1.74134 | 4.444003 | -0.32345 |
| C | -0.51823 | 2.664757 | -0.50181 | H | -1.69716 | 3.463813 | 1.131864 |
| C | -1.75286 | 3.406363 | 0.03507 | H | -3.06851 | 2.518908 | -1.43948 |
| C | -3.08065 | 2.730897 | -0.36042 | H | -3.91389 | 3.427185 | -0.19746 |
| C | -3.3496 | 1.431243 | 0.414933 | H | -3.5174 | 1.676893 | 1.473946 |
| C | -4.5355 | 0.613296 | -0.11264 | H | -2.45175 | 0.805342 | 0.386059 |
| C | -4.85217 | -0.64276 | 0.722941 | H | -4.33839 | 0.330399 | -1.15548 |
| C | -3.70101 | -1.65452 | 0.872315 | H | -5.43357 | 1.245646 | -0.13986 |
| C | -3.29118 | -2.36185 | -0.42881 | H | -5.72261 | -1.15122 | 0.285911 |
| C | 2.930942 | -0.24049 | -0.20817 | H | -5.16165 | -0.32336 | 1.727358 |
| C | 4.411637 | 0.081237 | 0.118718 | H | -3.99491 | -2.41644 | 1.606504 |
| C | 4.681887 | 0.44147 | 1.577362 | H | -2.82709 | -1.14962 | 1.301595 |
| C | 2.757743 | -0.5028 | -1.71174 | H | -3.14598 | -1.63193 | -1.23117 |
| O | 2.603701 | -1.46243 | 0.513001 | H | -4.09967 | -3.02954 | -0.74955 |
| O | 5.123637 | -1.11761 | -0.24302 | H | 5.760782 | 0.529486 | 1.748082 |
| H | 4.734994 | 0.908918 | -0.52877 | H | 4.232625 | 1.401782 | 1.843457 |
| C | -1.99652 | -3.17245 | -0.287 | H | 4.285167 | -0.32887 | 2.244187 |
| C | -0.75157 | -2.30189 | -0.26532 | H | 3.389749 | -1.33661 | -2.02672 |
| O | 0.283938 | -2.89014 | 0.338013 | H | 1.713426 | -0.74645 | -1.92317 |
| O | -0.69029 | -1.19269 | -0.77738 | H | 3.039222 | 0.372274 | -2.30507 |
| H | 2.030009 | 0.869513 | 1.400871 | H | 3.371809 | -2.04492 | 0.378622 |
| H | 0.941971 | 0.374455 | 0.118068 | H | 6.010539 | -1.06993 | 0.13742 |
| H | 2.033898 | 2.158237 | -1.38904 | H | -1.87573 | -3.85488 | -1.13918 |
| H | 2.941741 | 2.701916 | 0.010931 | H | -2.00483 | -3.80291 | 0.608243 |
| H | 1.02546 | 4.141139 | -0.14575 | H | 1.084897 | -2.29594 | 0.307184 |
| H | 0.724139 | 3.081349 | 1.221199 |  |  |  |  |

**Table S3:** (continued)

| **Conformer 12** | | | | | | | |
| --- | --- | --- | --- | --- | --- | --- | --- |
| Atom | X | Y | Z | Atom | X | Y | Z |
| C | 1.586771 | 0.904474 | -0.4408 | H | -1.04615 | 1.446524 | -1.03031 |
| C | 1.506311 | 2.422637 | -0.20803 | H | -0.60662 | 2.871638 | -1.94835 |
| C | 0.079098 | 2.909395 | 0.099911 | H | -3.02254 | 2.807879 | -1.53671 |
| C | -0.96393 | 2.538277 | -0.964 | H | -2.30011 | 4.230524 | -0.81333 |
| C | -2.36107 | 3.137128 | -0.72518 | H | -4.00809 | 3.261307 | 0.655832 |
| C | -3.00925 | 2.804248 | 0.632284 | H | -2.43979 | 3.294139 | 1.432201 |
| C | -3.14099 | 1.31063 | 0.976785 | H | -3.55951 | 1.231925 | 1.989901 |
| C | -4.02963 | 0.501343 | 0.020334 | H | -2.14354 | 0.857123 | 1.031887 |
| C | -4.34925 | -0.9182 | 0.524623 | H | -3.56805 | 0.452089 | -0.97425 |
| C | -3.16204 | -1.89572 | 0.59997 | H | -4.97851 | 1.03834 | -0.11646 |
| C | -2.66652 | -2.38207 | -0.76877 | H | -5.12487 | -1.35996 | -0.11598 |
| C | 2.992911 | 0.286582 | -0.40834 | H | -4.79632 | -0.83386 | 1.524373 |
| C | 3.65116 | 0.276441 | 1.006559 | H | -3.47186 | -2.77378 | 1.182298 |
| C | 2.729771 | -0.17326 | 2.134524 | H | -2.33267 | -1.44579 | 1.158509 |
| C | 3.925275 | 0.942281 | -1.43737 | H | -2.33628 | -1.53872 | -1.38707 |
| O | 2.820125 | -1.10197 | -0.7885 | H | -3.50241 | -2.84833 | -1.30478 |
| O | 4.742166 | -0.66997 | 0.979809 | H | 3.321722 | -0.33029 | 3.039864 |
| H | 4.042201 | 1.277115 | 1.227074 | H | 1.972938 | 0.584666 | 2.35125 |
| C | -1.52285 | -3.42047 | -0.68441 | H | 2.220101 | -1.10658 | 1.878022 |
| C | -0.21439 | -2.80239 | -0.23587 | H | 4.88149 | 0.412457 | -1.49338 |
| O | 0.482612 | -2.30296 | -1.26201 | H | 3.469623 | 0.905242 | -2.43093 |
| O | 0.155161 | -2.73674 | 0.927802 | H | 4.128342 | 1.987923 | -1.18802 |
| H | 0.966064 | 0.384348 | 0.296807 | H | 3.581386 | -1.5687 | -0.3976 |
| H | 1.159769 | 0.662969 | -1.42082 | H | 5.500953 | -0.25413 | 0.548403 |
| H | 1.889521 | 2.950137 | -1.09043 | H | -1.37266 | -3.87781 | -1.6664 |
| H | 2.155629 | 2.713631 | 0.627457 | H | -1.78785 | -4.20678 | 0.028453 |
| H | 0.098051 | 4.000856 | 0.222637 | H | 1.327597 | -1.88027 | -0.94165 |
| H | -0.22632 | 2.499285 | 1.071346 |  |  |  |  |

**Table S3:** (continued)

| **Conformer 13** | | | | | | | |
| --- | --- | --- | --- | --- | --- | --- | --- |
| Atom | X | Y | Z | Atom | X | Y | Z |
| C | 1.77922 | 0.818329 | -0.8597 | H | -0.88921 | 2.026208 | -1.36294 |
| C | 1.920003 | 2.315684 | -0.54323 | H | -1.1324 | 3.743862 | -1.59789 |
| C | 0.929099 | 3.217259 | -1.30421 | H | -0.43643 | 4.069469 | 0.872575 |
| C | -0.56749 | 3.012043 | -1.00419 | H | -0.53462 | 2.331444 | 1.051972 |
| C | -0.93751 | 3.174836 | 0.477278 | H | -2.80635 | 4.249684 | 0.330785 |
| C | -2.44336 | 3.308765 | 0.766486 | H | -2.57235 | 3.408557 | 1.852905 |
| C | -3.34354 | 2.163238 | 0.269026 | H | -3.41352 | 2.190183 | -0.82675 |
| C | -2.90148 | 0.757698 | 0.697697 | H | -4.36203 | 2.349249 | 0.636777 |
| C | -3.98384 | -0.31733 | 0.498491 | H | -2.60428 | 0.763898 | 1.755864 |
| C | -3.41088 | -1.7473 | 0.485814 | H | -2.00144 | 0.475065 | 0.136117 |
| C | -2.92885 | -2.17295 | -0.91148 | H | -4.51788 | -0.13685 | -0.44535 |
| C | 2.864753 | -0.09632 | -0.26406 | H | -4.73466 | -0.21536 | 1.291832 |
| C | 2.922714 | -0.0823 | 1.287038 | H | -4.16444 | -2.4661 | 0.830664 |
| C | 1.582933 | -0.29258 | 1.987926 | H | -2.58222 | -1.81033 | 1.203819 |
| C | 4.25163 | 0.206171 | -0.85113 | H | -2.41675 | -1.33949 | -1.40661 |
| O | 2.510629 | -1.4493 | -0.6616 | H | -3.80051 | -2.40915 | -1.53265 |
| O | 3.827104 | -1.15627 | 1.611743 | H | 1.739365 | -0.36098 | 3.070476 |
| H | 3.360634 | 0.873151 | 1.607985 | H | 0.902445 | 0.545274 | 1.815573 |
| C | -1.97837 | -3.39093 | -0.90502 | H | 1.102505 | -1.2143 | 1.647961 |
| C | -0.59719 | -3.00517 | -0.41715 | H | 4.982901 | -0.51878 | -0.48717 |
| O | 0.064565 | -2.28147 | -1.32641 | H | 4.213496 | 0.147891 | -1.94265 |
| O | -0.14347 | -3.28458 | 0.683385 | H | 4.599048 | 1.204131 | -0.56903 |
| H | 0.800983 | 0.452768 | -0.53057 | H | 2.99086 | -2.02507 | -0.03975 |
| H | 1.80666 | 0.676275 | -1.948 | H | 3.721448 | -1.36831 | 2.548625 |
| H | 2.932009 | 2.648951 | -0.79966 | H | -1.88407 | -3.78719 | -1.92114 |
| H | 1.813031 | 2.483939 | 0.535251 | H | -2.3653 | -4.18283 | -0.25831 |
| H | 1.09411 | 3.089306 | -2.38257 | H | 0.955169 | -1.99768 | -0.97322 |
| H | 1.186027 | 4.261133 | -1.07939 |  |  |  |  |

**Table S4.** Specific rotation calculation of **2a** (14*S*, 15*S*) (A) Boltzmann distribution of energy minimized conformers, and calculated optical rotation value, (B) Optimized Z-matrixes of **2a** conformers in the MeOH (Ǻ)

(A)

| Conformer | Calculated Energy (G)  (atomic units) | Relative Energy (kcal/mol) | Boltzmann Weights (%) | ORD Value | |
| --- | --- | --- | --- | --- | --- |
| 1 | -969.5697 | 0.000000 | 97.921088658 | 7.68 | **+8.05** |
| 2 | -969.566695 | 1.885666 | 0.000139954 | 95.43 |  |
| 3 | -969.565698 | 2.511293 | 0.000001610 | 62.69 |  |
| 4 | -969.568601 | 0.689633 | 0.713280647 | 80.74 |  |
| 5 | -969.566278 | 2.147338 | 0.000021622 | 12.4 |  |
| 6 | -969.566609 | 1.939632 | 0.000095216 | 0.85 |  |
| 7 | -969.567114 | 1.622740 | 0.000914036 | -33.52 |  |
| 8 | -969.566007 | 2.317393 | 0.000006424 | 46.43 |  |
| 9 | -969.566632 | 1.925199 | 0.000105547 | -2.36 |  |
| 10 | -969.567476 | 1.395581 | 0.004624590 | 84.9 |  |
| 11 | -969.568744 | 0.599899 | 1.353328883 | -4.27 |  |
| 12 | -969.566664 | 1.905119 | 0.000121812 | 46.92 |  |
| 13 | -969.567544 | 1.352910 | 0.006271002 | 52.51 |  |
| 14 | -969.566749 | 1.851781 | 0.000178245 | 77.04 |  |

(B)

| **Conformer 1** | | | | | | | |
| --- | --- | --- | --- | --- | --- | --- | --- |
| Atom | X | Y | Z | Atom | X | Y | Z |
| C | 2.685816 | -0.50311 | 1.27339 | H | -0.52289 | 4.220266 | -1.36568 |
| C | 2.929169 | -0.06096 | -0.19406 | H | -0.15852 | 2.546868 | -1.70804 |
| C | 1.977466 | 1.06791 | -0.62672 | H | -1.23524 | 3.585968 | 0.961757 |
| C | 1.940121 | 2.307793 | 0.273991 | H | -0.88332 | 1.908394 | 0.600569 |
| C | 1.246955 | 3.517334 | -0.37756 | H | -2.92977 | 3.647939 | -0.8611 |
| C | -0.17866 | 3.288693 | -0.90189 | H | -2.55981 | 1.997026 | -1.29266 |
| C | -1.20486 | 2.850575 | 0.147817 | H | -4.65305 | 2.206427 | 0.033768 |
| C | -2.61006 | 2.68661 | -0.44288 | H | -3.77289 | 2.947909 | 1.3534 |
| C | -3.68453 | 2.210262 | 0.547341 | H | -2.52026 | 0.837592 | 1.752039 |
| C | -3.45201 | 0.827701 | 1.176298 | H | -4.24909 | 0.63977 | 1.904538 |
| C | -3.40673 | -0.33712 | 0.18095 | H | -2.65408 | -0.12814 | -0.58457 |
| C | -3.08663 | -1.67219 | 0.860857 | H | -4.36678 | -0.40738 | -0.34571 |
| C | -3.02336 | -2.89292 | -0.06961 | H | -2.13461 | -1.57773 | 1.391815 |
| C | -2.0422 | -2.78301 | -1.24423 | H | -3.84201 | -1.87245 | 1.628695 |
| C | -0.60997 | -2.51786 | -0.84824 | H | -4.0147 | -3.08153 | -0.49343 |
| O | 0.077135 | -1.86961 | -1.78763 | H | -2.7697 | -3.77406 | 0.52517 |
| C | 3.52107 | -1.70103 | 1.725542 | H | -2.03677 | -3.72488 | -1.80476 |
| O | 1.301894 | -0.73709 | 1.52849 | H | -2.34628 | -2.01471 | -1.95589 |
| O | 2.592459 | -1.16207 | -1.09698 | H | 1.006903 | -1.68874 | -1.47925 |
| C | 4.390865 | 0.323672 | -0.4282 | H | 4.591978 | -1.50299 | 1.664938 |
| O | -0.11263 | -2.85969 | 0.212545 | H | 3.280385 | -1.92781 | 2.765134 |
| H | 2.946577 | 0.351831 | 1.901611 | H | 3.299456 | -2.59333 | 1.133476 |
| H | 0.973432 | 0.64809 | -0.6973 | H | 1.010743 | -1.53818 | 1.057153 |
| H | 2.269196 | 1.358397 | -1.64134 | H | 3.263457 | -1.85329 | -1.02967 |
| H | 1.441239 | 2.057452 | 1.213622 | H | 4.519221 | 0.693375 | -1.44675 |
| H | 2.956982 | 2.613317 | 0.539194 | H | 4.708003 | 1.10575 | 0.263781 |
| H | 1.866281 | 3.864605 | -1.21247 | H | 5.058567 | -0.5288 | -0.2903 |
| H | 1.231753 | 4.336627 | 0.350102 |  |  |  |  |

**Table S4:** (continued)

| **Conformer 2** | | | | | | | |
| --- | --- | --- | --- | --- | --- | --- | --- |
| Atom | X | Y | Z | Atom | X | Y | Z |
| C | 3.30727 | 0.837407 | -0.48773 | H | -0.59219 | -2.24118 | -1.14414 |
| C | 3.221502 | -0.63928 | -0.01792 | H | -0.12464 | -3.90504 | -0.88456 |
| C | 1.737673 | -1.03941 | 0.12868 | H | -2.24305 | -4.32778 | 0.364318 |
| C | 1.455435 | -2.38304 | 0.81263 | H | -2.47952 | -3.8911 | -1.31541 |
| C | -0.0435 | -2.71536 | 0.900463 | H | -4.13947 | -2.85313 | 0.203007 |
| C | -0.68875 | -3.07524 | -0.44319 | H | -2.87503 | -2.00487 | 1.060057 |
| C | -2.16377 | -3.49446 | -0.34356 | H | -3.45558 | -1.66829 | -1.92477 |
| C | -3.15182 | -2.39434 | 0.075675 | H | -2.32383 | -0.70778 | -1.00439 |
| C | -3.27502 | -1.24352 | -0.93023 | H | -4.48266 | 0.458229 | -1.45707 |
| C | -4.40499 | -0.24844 | -0.62454 | H | -5.35597 | -0.79336 | -0.60728 |
| C | -4.27792 | 0.530992 | 0.694937 | H | -4.30222 | -0.17123 | 1.534023 |
| C | -3.02996 | 1.414635 | 0.83705 | H | -5.16726 | 1.161649 | 0.80831 |
| C | -2.9495 | 2.564503 | -0.17232 | H | -3.02101 | 1.836374 | 1.848317 |
| C | -1.75131 | 3.496917 | 0.06575 | H | -2.12863 | 0.796687 | 0.76236 |
| C | -0.42381 | 2.820775 | -0.19723 | H | -3.86276 | 3.164596 | -0.11129 |
| O | 0.421531 | 2.893674 | 0.834066 | H | -2.89586 | 2.178162 | -1.19268 |
| C | 4.693879 | 1.313677 | -0.90415 | H | -1.80887 | 4.346851 | -0.62088 |
| O | 2.81319 | 1.694466 | 0.565996 | H | -1.76389 | 3.892299 | 1.082108 |
| O | 3.885054 | -0.62366 | 1.270416 | H | 1.274503 | 2.422773 | 0.623249 |
| C | 3.963613 | -1.57662 | -0.97217 | H | 5.035431 | 0.806724 | -1.80741 |
| O | -0.14208 | 2.268044 | -1.2436 | H | 4.653176 | 2.383484 | -1.1144 |
| H | 2.611352 | 0.968664 | -1.31802 | H | 5.425248 | 1.144378 | -0.11139 |
| H | 1.30842 | -1.04129 | -0.8767 | H | 3.140735 | 1.318179 | 1.398545 |
| H | 1.221941 | -0.25429 | 0.686826 | H | 3.689002 | -1.44354 | 1.738385 |
| H | 1.966062 | -3.19929 | 0.292246 | H | 3.849011 | -2.61503 | -0.65817 |
| H | 1.853276 | -2.3673 | 1.834026 | H | 3.570491 | -1.48899 | -1.98811 |
| H | -0.56782 | -1.87046 | 1.359281 | H | 5.028535 | -1.34857 | -0.99035 |
| H | -0.17268 | -3.56193 | 1.583973 |  |  |  |  |

**Table S4:** (continued)

| **Conformer 3** | | | | | | | |
| --- | --- | --- | --- | --- | --- | --- | --- |
| Atom | X | Y | Z | Atom | X | Y | Z |
| C | 3.447113 | -0.3434 | 0.995599 | H | -0.55863 | 2.41523 | 1.005545 |
| C | 2.978976 | 0.376888 | -0.29275 | H | -0.2142 | 3.909442 | 0.166932 |
| C | 1.665537 | 1.128071 | -0.04164 | H | -2.50342 | 4.080926 | -0.66066 |
| C | 1.108104 | 1.903069 | -1.24642 | H | -2.48629 | 3.920493 | 1.083769 |
| C | -0.39396 | 2.204354 | -1.13882 | H | -4.31138 | 2.625441 | 0.100065 |
| C | -0.80911 | 2.990155 | 0.109227 | H | -3.21653 | 1.755473 | -0.94509 |
| C | -2.29734 | 3.374805 | 0.15207 | H | -3.16302 | 1.615321 | 2.111126 |
| C | -3.29755 | 2.211425 | 0.045933 | H | -2.15838 | 0.667172 | 1.044412 |
| C | -3.14412 | 1.134837 | 1.125819 | H | -4.04648 | -0.64414 | 1.932647 |
| C | -4.22959 | 0.046088 | 1.102587 | H | -5.19997 | 0.511416 | 1.310515 |
| C | -4.35833 | -0.74891 | -0.20867 | H | -4.67849 | -0.07273 | -1.0071 |
| C | -3.09696 | -1.48755 | -0.67921 | H | -5.1701 | -1.47627 | -0.09257 |
| C | -2.66984 | -2.65594 | 0.214618 | H | -3.27777 | -1.87299 | -1.68868 |
| C | -1.40939 | -3.37875 | -0.29529 | H | -2.26955 | -0.77726 | -0.77697 |
| C | -0.16437 | -2.53477 | -0.1393 | H | -3.48301 | -3.38629 | 0.266294 |
| O | 0.423224 | -2.22313 | -1.29858 | H | -2.48868 | -2.31674 | 1.237564 |
| C | 3.949944 | 0.556032 | 2.116712 | H | -1.24831 | -4.28535 | 0.293789 |
| O | 4.49483 | -1.25103 | 0.577336 | H | -1.5298 | -3.67167 | -1.33895 |
| O | 2.672129 | -0.67928 | -1.25219 | H | 1.224929 | -1.65061 | -1.1462 |
| C | 4.071651 | 1.265981 | -0.88991 | H | 3.178699 | 1.253239 | 2.448484 |
| O | 0.263611 | -2.16536 | 0.937822 | H | 4.232164 | -0.05577 | 2.976288 |
| H | 2.596872 | -0.93473 | 1.346345 | H | 4.826391 | 1.125698 | 1.804418 |
| H | 1.817207 | 1.815582 | 0.792527 | H | 4.522461 | -1.99321 | 1.191617 |
| H | 0.93022 | 0.391498 | 0.295615 | H | 3.413733 | -1.30293 | -1.1897 |
| H | 1.658632 | 2.841468 | -1.36764 | H | 3.770782 | 1.613686 | -1.87781 |
| H | 1.27548 | 1.325556 | -2.15964 | H | 4.255017 | 2.140088 | -0.26239 |
| H | -0.93734 | 1.25455 | -1.17062 | H | 5.007488 | 0.713985 | -0.99093 |
| H | -0.70375 | 2.760332 | -2.03144 |  |  |  |  |

**Table S4:** (continued)

| **Conformer 4** | | | | | | | |
| --- | --- | --- | --- | --- | --- | --- | --- |
| Atom | X | Y | Z | Atom | X | Y | Z |
| C | -4.39603 | 0.315891 | -0.45873 | H | 0.713087 | 1.404153 | 0.800843 |
| C | -3.10029 | -0.205 | 0.200795 | H | 0.959741 | 1.644389 | -0.91534 |
| C | -2.12728 | 0.917734 | 0.592334 | H | 1.666658 | 3.681582 | 1.256119 |
| C | -1.75402 | 1.928015 | -0.50009 | H | 2.022972 | 3.863938 | -0.45099 |
| C | -0.60362 | 2.866266 | -0.09911 | H | 3.973906 | 3.082515 | 1.021328 |
| C | 0.756352 | 2.172567 | 0.022218 | H | 3.09225 | 1.597643 | 1.299057 |
| C | 1.91641 | 3.12037 | 0.348558 | H | 4.231523 | 2.640575 | -1.33581 |
| C | 3.253378 | 2.394464 | 0.56532 | H | 3.095668 | 1.318148 | -1.30722 |
| C | 3.869529 | 1.813414 | -0.71499 | H | 5.444712 | 0.554979 | -1.4614 |
| C | 5.027473 | 0.829023 | -0.48566 | H | 5.83303 | 1.337639 | 0.056509 |
| C | 4.663803 | -0.45962 | 0.272422 | H | 4.400308 | -0.22112 | 1.308335 |
| C | 3.521878 | -1.26433 | -0.35506 | H | 5.558324 | -1.0901 | 0.330159 |
| C | 3.21393 | -2.56736 | 0.388226 | H | 2.617122 | -0.64945 | -0.37852 |
| C | 1.968429 | -3.28587 | -0.15067 | H | 3.762612 | -1.49172 | -1.40073 |
| C | 0.690149 | -2.52486 | 0.132584 | H | 3.069249 | -2.36155 | 1.452557 |
| O | -0.09697 | -2.39507 | -0.93999 | H | 4.066996 | -3.24797 | 0.312783 |
| C | -5.30284 | 1.14051 | 0.443955 | H | 2.053286 | -3.46808 | -1.22281 |
| O | -5.0851 | -0.85872 | -0.94591 | H | 1.863109 | -4.25975 | 0.338091 |
| O | -2.42744 | -1.0025 | -0.81499 | H | -0.92117 | -1.8854 | -0.71564 |
| C | -3.38036 | -1.10114 | 1.411113 | H | -4.78605 | 2.023757 | 0.824404 |
| O | 0.39461 | -2.08019 | 1.224619 | H | -6.17213 | 1.483485 | -0.1212 |
| H | -4.1001 | 0.915056 | -1.32349 | H | -5.65869 | 0.552709 | 1.291096 |
| H | -1.22688 | 0.41747 | 0.956738 | H | -5.6702 | -0.59581 | -1.66542 |
| H | -2.54532 | 1.449531 | 1.452016 | H | -3.12284 | -1.52377 | -1.24627 |
| H | -1.47645 | 1.393048 | -1.41349 | H | -2.44035 | -1.52659 | 1.764993 |
| H | -2.62563 | 2.539455 | -0.75155 | H | -3.82911 | -0.5353 | 2.229307 |
| H | -0.8487 | 3.362397 | 0.84812 | H | -4.0549 | -1.91666 | 1.146498 |
| H | -0.52812 | 3.662291 | -0.84823 |  |  |  |  |

**Table S4:** (continued)

| **Conformer 5** | | | | | | | |
| --- | --- | --- | --- | --- | --- | --- | --- |
| Atom | X | Y | Z | Atom | X | Y | Z |
| C | 3.963726 | -0.06915 | -0.2628 | H | -2.08469 | -3.68806 | -1.72507 |
| C | 2.849335 | -0.91054 | 0.435407 | H | -1.20992 | -4.16817 | -0.28694 |
| C | 1.785876 | -1.29676 | -0.61935 | H | -2.18399 | -2.1561 | 0.918903 |
| C | 0.546651 | -2.04158 | -0.11021 | H | -3.33808 | -3.37635 | 0.436371 |
| C | -0.40414 | -2.42441 | -1.25242 | H | -3.95995 | -2.05116 | -1.56945 |
| C | -1.59697 | -3.29534 | -0.8253 | H | -2.84305 | -0.78926 | -1.1126 |
| C | -2.6616 | -2.60778 | 0.044404 | H | -5.25318 | -1.7306 | 0.510825 |
| C | -3.49681 | -1.56194 | -0.70409 | H | -5.25742 | -0.32359 | -0.52615 |
| C | -4.61749 | -0.92094 | 0.134042 | H | -5.01993 | -0.03025 | 2.045568 |
| C | -4.19131 | -0.04719 | 1.329929 | H | -3.35682 | -0.52047 | 1.85826 |
| C | -3.82492 | 1.414121 | 1.02478 | H | -3.64453 | 1.917701 | 1.981946 |
| C | -2.59974 | 1.625193 | 0.128204 | H | -4.69056 | 1.917992 | 0.57778 |
| C | -2.07516 | 3.063644 | 0.151211 | H | -1.79978 | 0.950437 | 0.447261 |
| C | -0.90429 | 3.296115 | -0.80549 | H | -2.8426 | 1.355979 | -0.90436 |
| C | 0.344058 | 2.520948 | -0.4601 | H | -1.77187 | 3.327977 | 1.168008 |
| O | 1.146249 | 2.331526 | -1.50918 | H | -2.87846 | 3.753652 | -0.12527 |
| C | 5.256049 | 0.097455 | 0.523438 | H | -1.17896 | 3.060632 | -1.83622 |
| O | 3.455094 | 1.251028 | -0.59412 | H | -0.61367 | 4.353059 | -0.80423 |
| O | 2.277854 | -0.1376 | 1.50168 | H | 1.972322 | 1.861888 | -1.21416 |
| C | 3.446575 | -2.15579 | 1.089741 | H | 5.775209 | -0.8528 | 0.644212 |
| O | 0.61958 | 2.118968 | 0.658174 | H | 5.921543 | 0.777335 | -0.0113 |
| H | 4.183391 | -0.52747 | -1.22892 | H | 5.061103 | 0.509768 | 1.517098 |
| H | 2.285246 | -1.9133 | -1.37505 | H | 3.549964 | 1.801539 | 0.195272 |
| H | 1.460683 | -0.39116 | -1.14027 | H | 1.749165 | 0.590903 | 1.129427 |
| H | 0.844814 | -2.95705 | 0.40979 | H | 2.658039 | -2.77874 | 1.510382 |
| H | 0.025471 | -1.42427 | 0.625855 | H | 4.001755 | -2.75216 | 0.362316 |
| H | 0.167454 | -2.9771 | -2.00676 | H | 4.121241 | -1.88122 | 1.900225 |
| H | -0.7592 | -1.51734 | -1.75358 |  |  |  |  |

**Table S4:** (continued)

| **Conformer 6** | | | | | | | |
| --- | --- | --- | --- | --- | --- | --- | --- |
| Atom | X | Y | Z | Atom | X | Y | Z |
| C | 3.591618 | -0.69297 | 0.913309 | H | -1.11018 | 1.908418 | -1.40999 |
| C | 3.202646 | 0.240225 | -0.25961 | H | -0.73992 | 1.362579 | 0.210448 |
| C | 2.134766 | 1.246684 | 0.186929 | H | -2.20806 | 3.955098 | -0.47098 |
| C | 1.639505 | 2.22003 | -0.89202 | H | -1.86809 | 3.396058 | 1.15579 |
| C | 0.42881 | 3.052684 | -0.43842 | H | -4.23379 | 2.928396 | 0.319343 |
| C | -0.88087 | 2.260254 | -0.39649 | H | -3.54858 | 1.858068 | -0.88328 |
| C | -2.08055 | 3.051562 | 0.136046 | H | -3.44719 | 1.542549 | 2.156662 |
| C | -3.39322 | 2.252291 | 0.126623 | H | -2.5064 | 0.531841 | 1.092454 |
| C | -3.43231 | 1.109558 | 1.149988 | H | -4.57661 | -0.57248 | 1.835225 |
| C | -4.62363 | 0.14968 | 1.01361 | H | -5.55735 | 0.705062 | 1.158969 |
| C | -4.71967 | -0.60616 | -0.32532 | H | -5.01256 | 0.093113 | -1.11446 |
| C | -3.44544 | -1.33111 | -0.78249 | H | -5.53805 | -1.33166 | -0.25364 |
| C | -2.97713 | -2.46222 | 0.138297 | H | -3.62341 | -1.74704 | -1.78034 |
| C | -1.67002 | -3.11754 | -0.33879 | H | -2.63734 | -0.60279 | -0.90577 |
| C | -0.46596 | -2.21888 | -0.15416 | H | -3.75074 | -3.23441 | 0.188271 |
| O | 0.211995 | -1.99483 | -1.28493 | H | -2.83257 | -2.09821 | 1.158244 |
| C | 4.356975 | -0.04347 | 2.058447 | H | -1.47493 | -4.01628 | 0.253601 |
| O | 4.379056 | -1.75377 | 0.323915 | H | -1.74637 | -3.41986 | -1.38394 |
| O | 2.577271 | -0.61667 | -1.26044 | H | 1.010851 | -1.42824 | -1.11054 |
| C | 4.424868 | 0.907254 | -0.89518 | H | 3.777943 | 0.754812 | 2.525628 |
| O | -0.13389 | -1.74869 | 0.916606 | H | 4.566777 | -0.79091 | 2.826733 |
| H | 2.658472 | -1.12271 | 1.289849 | H | 5.308261 | 0.366904 | 1.716418 |
| H | 2.53854 | 1.830251 | 1.018452 | H | 4.303059 | -2.53798 | 0.878927 |
| H | 1.295397 | 0.672039 | 0.586636 | H | 3.177807 | -1.37329 | -1.35602 |
| H | 2.451498 | 2.898234 | -1.16725 | H | 4.139225 | 1.405134 | -1.82128 |
| H | 1.375249 | 1.667836 | -1.79926 | H | 4.859285 | 1.653083 | -0.22697 |
| H | 0.306853 | 3.903311 | -1.11781 | H | 5.19221 | 0.166987 | -1.12694 |
| H | 0.63291 | 3.480555 | 0.550968 |  |  |  |  |

**Table S4:** (continued)

| **Conformer 7** | | | | | | | |
| --- | --- | --- | --- | --- | --- | --- | --- |
| Atom | X | Y | Z | Atom | X | Y | Z |
| C | -2.32732 | -0.22427 | 0.997013 | H | 0.631317 | -3.84826 | 0.37674 |
| C | -2.71785 | -1.10763 | -0.2206 | H | 2.003202 | -4.2173 | -0.64005 |
| C | -1.62653 | -2.14922 | -0.55086 | H | 2.58926 | -3.02335 | 1.486086 |
| C | -0.3141 | -1.61221 | -1.13399 | H | 1.665587 | -1.63766 | 0.966516 |
| C | 0.704838 | -2.71624 | -1.46762 | H | 4.155582 | -2.77792 | -0.39613 |
| C | 1.390454 | -3.38933 | -0.26529 | H | 3.230218 | -1.45741 | -1.06108 |
| C | 2.271245 | -2.46906 | 0.595498 | H | 5.259227 | -0.68873 | 0.146145 |
| C | 3.517889 | -1.93078 | -0.11846 | H | 4.69839 | -1.44639 | 1.622209 |
| C | 4.354983 | -0.94211 | 0.711579 | H | 2.747825 | 0.105884 | 1.705915 |
| C | 3.63462 | 0.355236 | 1.11404 | H | 4.285289 | 0.926895 | 1.784526 |
| C | 3.21247 | 1.24857 | -0.05998 | H | 2.79023 | 0.633917 | -0.86073 |
| C | 2.163675 | 2.292669 | 0.330335 | H | 4.094956 | 1.739222 | -0.48722 |
| C | 1.677926 | 3.146208 | -0.84737 | H | 1.31185 | 1.761145 | 0.770203 |
| C | 0.356892 | 3.879113 | -0.55832 | H | 2.551357 | 2.944281 | 1.121738 |
| C | -0.78396 | 2.90066 | -0.39745 | H | 1.533582 | 2.512459 | -1.72664 |
| O | -1.43637 | 3.014568 | 0.75759 | H | 2.437387 | 3.885746 | -1.11467 |
| C | -2.41467 | -0.88405 | 2.361994 | H | 0.4353 | 4.499778 | 0.334791 |
| O | -3.16758 | 0.962148 | 1.015042 | H | 0.104214 | 4.531429 | -1.39903 |
| O | -2.93477 | -0.2122 | -1.33742 | H | -2.13208 | 2.299544 | 0.836666 |
| C | -4.05886 | -1.81197 | -0.01764 | H | -1.76021 | -1.7553 | 2.418196 |
| O | -1.07024 | 2.067192 | -1.24274 | H | -2.09441 | -0.17729 | 3.129715 |
| H | -1.30007 | 0.117191 | 0.838231 | H | -3.4349 | -1.19507 | 2.588845 |
| H | -2.05982 | -2.84896 | -1.27316 | H | -3.59981 | 0.991172 | 0.143797 |
| H | -1.42928 | -2.73261 | 0.352826 | H | -2.18616 | 0.406818 | -1.41222 |
| H | -0.5369 | -1.06884 | -2.0563 | H | -4.37827 | -2.25139 | -0.96385 |
| H | 0.143842 | -0.88552 | -0.45782 | H | -3.9752 | -2.61247 | 0.717979 |
| H | 1.469548 | -2.29247 | -2.12492 | H | -4.83149 | -1.11615 | 0.314742 |
| H | 0.201792 | -3.48955 | -2.05915 |  |  |  |  |

**Table S4:** (continued)

| **Conformer 8** | | | | | | | |
| --- | --- | --- | --- | --- | --- | --- | --- |
| Atom | X | Y | Z | Atom | X | Y | Z |
| C | 3.800333 | 0.09447 | 0.943879 | H | 0.516749 | 3.701813 | 0.015311 |
| C | 3.080061 | -0.56646 | -0.25238 | H | 0.222389 | 3.642504 | -1.7118 |
| C | 2.839518 | 0.403974 | -1.42183 | H | -1.94559 | 2.42023 | -1.26698 |
| C | 2.173734 | 1.766542 | -1.16048 | H | -1.90312 | 4.0753 | -0.69758 |
| C | 0.674776 | 1.723914 | -0.84789 | H | -1.39244 | 1.675385 | 1.126305 |
| C | 0.03024 | 3.112105 | -0.77194 | H | -1.47769 | 3.350768 | 1.609769 |
| C | -1.48507 | 3.077497 | -0.52114 | H | -3.86922 | 3.459069 | 0.925535 |
| C | -1.87774 | 2.626463 | 0.890735 | H | -3.55695 | 2.287647 | 2.189661 |
| C | -3.39007 | 2.493564 | 1.126036 | H | -5.16752 | 1.419678 | 0.577118 |
| C | -4.10608 | 1.409314 | 0.303797 | H | -4.07036 | 1.661026 | -0.7618 |
| C | -3.55638 | -0.00743 | 0.502393 | H | -3.50371 | -0.23129 | 1.574712 |
| C | -4.40122 | -1.07954 | -0.19278 | H | -2.52848 | -0.04846 | 0.132496 |
| C | -3.89688 | -2.52246 | -0.04208 | H | -5.42098 | -1.03169 | 0.205204 |
| C | -2.60385 | -2.84416 | -0.80726 | H | -4.48259 | -0.8415 | -1.26058 |
| C | -1.32622 | -2.3928 | -0.13463 | H | -4.67273 | -3.19701 | -0.4139 |
| O | -0.42478 | -1.91761 | -0.99941 | H | -3.75746 | -2.76415 | 1.015498 |
| C | 5.217485 | 0.580421 | 0.673457 | H | -2.50683 | -3.93136 | -0.90512 |
| O | 3.790215 | -0.89482 | 1.997959 | H | -2.63844 | -2.44257 | -1.82138 |
| O | 1.782867 | -0.9901 | 0.251457 | H | 0.39924 | -1.63178 | -0.51817 |
| C | 3.82184 | -1.80773 | -0.76179 | H | 5.236236 | 1.330667 | -0.11924 |
| O | -1.11153 | -2.48275 | 1.059046 | H | 5.624425 | 1.042147 | 1.575683 |
| H | 3.182313 | 0.936412 | 1.266359 | H | 5.872379 | -0.2444 | 0.389512 |
| H | 2.248985 | -0.14008 | -2.16676 | H | 3.869593 | -0.44196 | 2.845166 |
| H | 3.811796 | 0.578988 | -1.88946 | H | 1.947429 | -1.39655 | 1.116731 |
| H | 2.700419 | 2.307491 | -0.36757 | H | 3.225209 | -2.2913 | -1.53723 |
| H | 2.319359 | 2.366206 | -2.0663 | H | 4.790212 | -1.5479 | -1.19209 |
| H | 0.513466 | 1.182233 | 0.084588 | H | 3.984515 | -2.52267 | 0.045645 |
| H | 0.168315 | 1.143179 | -1.62831 |  |  |  |  |

**Table S4:** (continued)

| **Conformer 9** | | | | | | | |
| --- | --- | --- | --- | --- | --- | --- | --- |
| Atom | X | Y | Z | Atom | X | Y | Z |
| C | 2.098047 | -0.89398 | 1.141284 | H | -0.05602 | 4.140005 | -1.64325 |
| C | 2.736304 | -0.48878 | -0.23404 | H | 0.130819 | 2.421905 | -1.87876 |
| C | 2.143302 | 0.810462 | -0.80237 | H | -0.84171 | 3.822786 | 0.656047 |
| C | 2.264751 | 2.088411 | 0.033413 | H | -0.42714 | 2.124203 | 0.620827 |
| C | 1.686242 | 3.326381 | -0.67755 | H | -2.53666 | 3.413664 | -1.17339 |
| C | 0.220297 | 3.213824 | -1.12755 | H | -2.10561 | 1.720947 | -1.17437 |
| C | -0.78643 | 2.947046 | -0.00243 | H | -4.19049 | 2.103981 | 0.064115 |
| C | -2.18403 | 2.601734 | -0.52704 | H | -3.42613 | 3.287075 | 1.097896 |
| C | -3.24435 | 2.351394 | 0.556745 | H | -2.05663 | 1.604718 | 2.197932 |
| C | -2.90304 | 1.265774 | 1.592582 | H | -3.74666 | 1.175806 | 2.286689 |
| C | -2.57046 | -0.12788 | 1.038083 | H | -2.21814 | -0.7504 | 1.867471 |
| C | -3.74348 | -0.84243 | 0.358918 | H | -1.72753 | -0.04848 | 0.344901 |
| C | -3.46563 | -2.29389 | -0.06136 | H | -4.59332 | -0.84882 | 1.050915 |
| C | -2.4799 | -2.46126 | -1.22813 | H | -4.07267 | -0.27659 | -0.51963 |
| C | -1.01791 | -2.35058 | -0.86601 | H | -4.41077 | -2.74952 | -0.36781 |
| O | -0.29716 | -1.72203 | -1.78992 | H | -3.10703 | -2.87053 | 0.79602 |
| C | 2.827118 | -0.37993 | 2.374927 | H | -2.59177 | -3.4666 | -1.65023 |
| O | 2.04269 | -2.32532 | 1.250253 | H | -2.70044 | -1.76015 | -2.03455 |
| O | 2.323468 | -1.50018 | -1.20566 | H | 0.668114 | -1.67511 | -1.52887 |
| C | 4.263281 | -0.47964 | -0.21087 | H | 2.98144 | 0.698148 | 2.343816 |
| O | -0.53274 | -2.82329 | 0.151219 | H | 2.240108 | -0.61586 | 3.264002 |
| H | 1.07197 | -0.51293 | 1.145648 | H | 3.800602 | -0.86311 | 2.474047 |
| H | 1.086242 | 0.6131 | -0.99546 | H | 1.178607 | -2.60839 | 0.895737 |
| H | 2.607457 | 0.976972 | -1.78062 | H | 2.572109 | -2.35349 | -0.81607 |
| H | 1.758071 | 1.959303 | 0.992551 | H | 4.640739 | -0.32155 | -1.22252 |
| H | 3.314557 | 2.288752 | 0.267268 | H | 4.653286 | 0.31362 | 0.427505 |
| H | 2.301819 | 3.547337 | -1.55668 | H | 4.655513 | -1.4308 | 0.157263 |
| H | 1.792462 | 4.186522 | -0.00732 |  |  |  |  |

**Table S4:** (continued)

| **Conformer 10** | | | | | | | |
| --- | --- | --- | --- | --- | --- | --- | --- |
| Atom | X | Y | Z | Atom | X | Y | Z |
| C | -4.10833 | 0.451634 | -0.80682 | H | 1.021986 | 2.410993 | -1.18263 |
| C | -3.19857 | -0.2441 | 0.228541 | H | 0.258328 | 3.863155 | -0.58114 |
| C | -2.18152 | 0.707519 | 0.873667 | H | 1.903394 | 3.881043 | 1.353123 |
| C | -1.29906 | 1.528477 | -0.07424 | H | 2.595154 | 4.188633 | -0.22572 |
| C | -0.08761 | 2.128968 | 0.645581 | H | 3.950442 | 2.658105 | 1.269865 |
| C | 0.811092 | 2.971489 | -0.2653 | H | 2.684852 | 1.46019 | 1.154098 |
| C | 2.131127 | 3.409698 | 0.390115 | H | 4.325934 | 2.599679 | -1.16053 |
| C | 3.156708 | 2.285324 | 0.612347 | H | 3.027744 | 1.453806 | -1.38557 |
| C | 3.801619 | 1.766442 | -0.67847 | H | 5.339272 | 0.453161 | -1.41601 |
| C | 4.801414 | 0.617486 | -0.47513 | H | 5.556547 | 0.93146 | 0.254888 |
| C | 4.205494 | -0.72576 | -0.02182 | H | 3.720193 | -0.60658 | 0.951726 |
| C | 3.22594 | -1.34481 | -1.02743 | H | 5.032999 | -1.42665 | 0.13888 |
| C | 2.846786 | -2.80482 | -0.74236 | H | 2.315648 | -0.73996 | -1.08736 |
| C | 2.091681 | -3.04846 | 0.572717 | H | 3.678111 | -1.30583 | -2.02507 |
| C | 0.743581 | -2.37487 | 0.701677 | H | 3.757362 | -3.41207 | -0.716 |
| O | 0.017294 | -2.4162 | -0.41916 | H | 2.245173 | -3.18583 | -1.57069 |
| C | -5.05605 | 1.502035 | -0.24466 | H | 1.90396 | -4.12312 | 0.678904 |
| O | -4.84407 | -0.6109 | -1.45527 | H | 2.681348 | -2.749 | 1.438837 |
| O | -2.42707 | -1.23081 | -0.51152 | H | -0.8582 | -1.96698 | -0.28943 |
| C | -3.99866 | -0.97302 | 1.312115 | H | -4.50802 | 2.30442 | 0.253115 |
| O | 0.337113 | -1.87128 | 1.731486 | H | -5.63255 | 1.94964 | -1.05716 |
| H | -3.4542 | 0.91293 | -1.55135 | H | -5.75672 | 1.063856 | 0.467346 |
| H | -1.53773 | 0.083566 | 1.500549 | H | -5.13599 | -0.29966 | -2.31955 |
| H | -2.71595 | 1.375323 | 1.554866 | H | -3.04578 | -1.66401 | -1.11908 |
| H | -0.94514 | 0.889698 | -0.88892 | H | -3.31304 | -1.53248 | 1.950389 |
| H | -1.87939 | 2.332582 | -0.5393 | H | -4.55007 | -0.27086 | 1.939628 |
| H | 0.490613 | 1.310237 | 1.084923 | H | -4.71011 | -1.67199 | 0.870327 |
| H | -0.42616 | 2.746709 | 1.486511 |  |  |  |  |

**Table S4:** (continued)

| **Conformer 11** | | | | | | | |
| --- | --- | --- | --- | --- | --- | --- | --- |
| Atom | X | Y | Z | Atom | X | Y | Z |
| C | -3.88283 | -0.36234 | -0.77724 | H | -1.05415 | 4.380069 | -0.7209 |
| C | -2.88713 | -0.58729 | 0.382721 | H | -0.02029 | 4.749419 | 0.643273 |
| C | -2.40639 | 0.723406 | 1.02397 | H | 1.257648 | 4.137776 | -1.40198 |
| C | -1.74056 | 1.74428 | 0.095689 | H | 0.490054 | 2.57107 | -1.44756 |
| C | -1.13969 | 2.92407 | 0.869151 | H | 2.462686 | 3.595008 | 0.643781 |
| C | -0.36398 | 3.9298 | 0.001776 | H | 1.508609 | 2.151529 | 0.906133 |
| C | 0.837893 | 3.352486 | -0.76376 | H | 3.435447 | 2.518861 | -1.43355 |
| C | 1.948218 | 2.77784 | 0.123527 | H | 2.407477 | 1.129568 | -1.1587 |
| C | 2.959726 | 1.92338 | -0.64512 | H | 4.750196 | 2.086045 | 0.546478 |
| C | 4.040888 | 1.305607 | 0.251107 | H | 3.578251 | 0.958916 | 1.181358 |
| C | 4.814173 | 0.146125 | -0.39788 | H | 5.693075 | -0.08625 | 0.214142 |
| C | 4.00025 | -1.14296 | -0.59923 | H | 5.200006 | 0.476155 | -1.36892 |
| C | 3.641153 | -1.86515 | 0.704472 | H | 3.086343 | -0.91612 | -1.15832 |
| C | 2.728853 | -3.08598 | 0.498555 | H | 4.573053 | -1.82893 | -1.23255 |
| C | 1.340619 | -2.74711 | 0.003401 | H | 3.155764 | -1.18024 | 1.402157 |
| O | 0.706416 | -1.87036 | 0.787435 | H | 4.559794 | -2.20637 | 1.19158 |
| C | -5.22321 | 0.242684 | -0.38415 | H | 3.16813 | -3.78719 | -0.21168 |
| O | -4.06495 | -1.6593 | -1.38974 | H | 2.608212 | -3.61075 | 1.451702 |
| O | -1.71848 | -1.21647 | -0.21434 | H | -0.19848 | -1.672 | 0.421605 |
| C | -3.44755 | -1.52423 | 1.456848 | H | -5.09585 | 1.22427 | 0.07623 |
| O | 0.831931 | -3.2259 | -0.99334 | H | -5.84178 | 0.372973 | -1.27464 |
| H | -3.38907 | 0.287472 | -1.50411 | H | -5.76136 | -0.40267 | 0.311367 |
| H | -1.69296 | 0.444765 | 1.807007 | H | -4.3428 | -1.53121 | -2.30384 |
| H | -3.2528 | 1.182678 | 1.542243 | H | -2.05481 | -1.88571 | -0.83086 |
| H | -0.96131 | 1.237365 | -0.47659 | H | -2.66543 | -1.74829 | 2.1841 |
| H | -2.46041 | 2.130543 | -0.63363 | H | -4.28259 | -1.06502 | 1.988537 |
| H | -0.48692 | 2.536889 | 1.658399 | H | -3.79195 | -2.46164 | 1.01835 |
| H | -1.94385 | 3.459552 | 1.386294 |  |  |  |  |

**Table S4:** (continued)

| **Conformer 12** | | | | | | | |
| --- | --- | --- | --- | --- | --- | --- | --- |
| Atom | X | Y | Z | Atom | X | Y | Z |
| C | 2.22975 | -1.10402 | 1.170774 | H | 0.976513 | 4.501217 | 0.050927 |
| C | 2.644893 | -0.15285 | 0.002831 | H | -0.42208 | 4.653701 | -0.98696 |
| C | 1.755861 | 1.096294 | 0.02365 | H | -1.07707 | 4.079401 | 1.277771 |
| C | 2.050422 | 2.156884 | -1.05091 | H | -0.10689 | 2.629832 | 1.281741 |
| C | 0.83944 | 2.99737 | -1.49097 | H | -2.51436 | 3.136038 | -0.53617 |
| C | 0.188869 | 3.921967 | -0.44552 | H | -1.54992 | 1.675518 | -0.55026 |
| C | -0.70192 | 3.276893 | 0.631822 | H | -3.11834 | 2.718888 | 1.854259 |
| C | -1.90012 | 2.48371 | 0.097576 | H | -2.15226 | 1.263043 | 1.857744 |
| C | -2.77089 | 1.899277 | 1.214924 | H | -4.62186 | 0.871101 | 1.60115 |
| C | -4.00002 | 1.113774 | 0.731671 | H | -4.60798 | 1.770629 | 0.09888 |
| C | -3.72142 | -0.18667 | -0.04039 | H | -3.14864 | 0.036176 | -0.94556 |
| C | -3.00927 | -1.26854 | 0.782018 | H | -4.68206 | -0.58747 | -0.38429 |
| C | -2.97344 | -2.65551 | 0.123492 | H | -1.98867 | -0.95069 | 1.020886 |
| C | -2.17173 | -2.74539 | -1.18844 | H | -3.52234 | -1.37086 | 1.744878 |
| C | -0.69911 | -2.47989 | -0.98809 | H | -3.99645 | -2.97853 | -0.09173 |
| O | -0.23631 | -1.46731 | -1.71274 | H | -2.56016 | -3.37926 | 0.830469 |
| C | 2.848209 | -0.76506 | 2.519127 | H | -2.25734 | -3.76066 | -1.58587 |
| O | 2.57159 | -2.45942 | 0.845241 | H | -2.56234 | -2.06246 | -1.94161 |
| O | 2.326764 | -0.8443 | -1.24363 | H | 0.730235 | -1.29085 | -1.51474 |
| C | 4.140003 | 0.158284 | -0.0046 | H | 2.681027 | 0.279473 | 2.787958 |
| O | 0.001269 | -3.13275 | -0.22746 | H | 2.399201 | -1.38966 | 3.292924 |
| H | 1.140457 | -1.04185 | 1.256867 | H | 3.923307 | -0.95191 | 2.513313 |
| H | 1.82389 | 1.541378 | 1.018382 | H | 1.777012 | -2.85417 | 0.442558 |
| H | 0.72524 | 0.748335 | -0.08507 | H | 2.81457 | -1.68171 | -1.21547 |
| H | 2.846827 | 2.82405 | -0.70532 | H | 4.408821 | 0.688223 | -0.91841 |
| H | 2.43598 | 1.656472 | -1.94341 | H | 4.419495 | 0.781126 | 0.846253 |
| H | 0.08281 | 2.330163 | -1.91696 | H | 4.726657 | -0.76252 | 0.040881 |
| H | 1.173056 | 3.628945 | -2.32113 |  |  |  |  |

**Table S4:** (continued)

| **Conformer 13** | | | | | | | |
| --- | --- | --- | --- | --- | --- | --- | --- |
| Atom | X | Y | Z | Atom | X | Y | Z |
| C | 2.750589 | -0.39278 | 0.86484 | H | -1.6823 | 4.110737 | -1.01594 |
| C | 3.181422 | 0.60633 | -0.24495 | H | -1.56633 | 2.401238 | -1.37149 |
| C | 2.394501 | 1.932311 | -0.17245 | H | -1.90554 | 3.704293 | 1.374002 |
| C | 0.87024 | 1.844823 | -0.307 | H | -1.62616 | 1.992307 | 1.184539 |
| C | 0.209225 | 3.219508 | -0.47255 | H | -4.04172 | 2.644804 | 1.52163 |
| C | -1.31941 | 3.156649 | -0.61767 | H | -4.00394 | 3.511585 | 0.00068 |
| C | -2.06608 | 2.863096 | 0.690217 | H | -3.56805 | 1.356454 | -1.21648 |
| C | -3.58015 | 2.648649 | 0.52744 | H | -5.08494 | 1.382475 | -0.34849 |
| C | -3.99811 | 1.364656 | -0.20895 | H | -2.52971 | 0.070652 | 0.685854 |
| C | -3.60849 | 0.07038 | 0.512406 | H | -4.07621 | 0.052994 | 1.504902 |
| C | -3.99838 | -1.19842 | -0.25372 | H | -3.5757 | -1.14953 | -1.26359 |
| C | -3.57465 | -2.50927 | 0.430308 | H | -5.08533 | -1.21572 | -0.38903 |
| C | -2.05919 | -2.7028 | 0.592091 | H | -4.0313 | -2.54955 | 1.425175 |
| C | -1.3259 | -2.95025 | -0.74764 | H | -3.98758 | -3.3562 | -0.12868 |
| C | 0.103582 | -2.47977 | -0.71445 | H | -1.86832 | -3.54359 | 1.261759 |
| O | 0.838733 | -3.0625 | 0.230863 | H | -1.63395 | -1.82562 | 1.085123 |
| C | 3.245819 | -0.08715 | 2.267271 | H | -1.34052 | -4.01739 | -0.98587 |
| O | 3.187778 | -1.73258 | 0.505683 | H | -1.80908 | -2.42125 | -1.56706 |
| O | 2.953907 | -0.04755 | -1.51539 | H | 1.742563 | -2.63987 | 0.284321 |
| C | 4.681968 | 0.895719 | -0.21964 | H | 2.900654 | 0.894808 | 2.596024 |
| O | 0.543232 | -1.61846 | -1.46136 | H | 2.853192 | -0.82964 | 2.964418 |
| H | 1.657786 | -0.41526 | 0.878647 | H | 4.334753 | -0.11199 | 2.319077 |
| H | 2.784887 | 2.564273 | -0.97698 | H | 3.472925 | -1.6771 | -0.42262 |
| H | 2.649243 | 2.439931 | 0.763318 | H | 2.058831 | -0.43467 | -1.53265 |
| H | 0.608227 | 1.233073 | -1.17606 | H | 4.953049 | 1.458429 | -1.11436 |
| H | 0.444417 | 1.341261 | 0.564841 | H | 4.956214 | 1.489493 | 0.65246 |
| H | 0.632375 | 3.702899 | -1.35988 | H | 5.265879 | -0.02666 | -0.20756 |
| H | 0.470529 | 3.860908 | 0.378075 |  |  |  |  |

**Table S4:** (continued)

| **Conformer 14** | | | | | | | |
| --- | --- | --- | --- | --- | --- | --- | --- |
| Atom | X | Y | Z | Atom | X | Y | Z |
| C | 3.247676 | 0.93031 | -0.16799 | H | -0.15996 | -2.45489 | 0.9436 |
| C | 3.666524 | -0.51012 | 0.219402 | H | -0.79826 | -1.29307 | -0.20067 |
| C | 2.459602 | -1.47299 | 0.205633 | H | -1.46991 | -4.27443 | -0.28594 |
| C | 1.728941 | -1.65603 | -1.13009 | H | -2.22135 | -3.01448 | -1.23676 |
| C | 0.590844 | -2.68658 | -1.07444 | H | -3.66813 | -3.72736 | 0.635348 |
| C | -0.52811 | -2.34781 | -0.08252 | H | -2.39329 | -3.40744 | 1.793159 |
| C | -1.77398 | -3.22157 | -0.25831 | H | -2.54248 | -0.97299 | 1.344177 |
| C | -2.82678 | -3.05774 | 0.848972 | H | -4.04143 | -1.65352 | 1.928161 |
| C | -3.36669 | -1.63563 | 1.064235 | H | -3.46968 | -0.98971 | -1.00361 |
| C | -4.12725 | -1.04988 | -0.13081 | H | -4.92859 | -1.74463 | -0.40794 |
| C | -4.75334 | 0.326834 | 0.145129 | H | -5.36217 | 0.25447 | 1.053333 |
| C | -3.76541 | 1.493413 | 0.306262 | H | -5.44848 | 0.571997 | -0.66604 |
| C | -3.08502 | 1.916011 | -0.99991 | H | -4.30821 | 2.356218 | 0.70769 |
| C | -2.15015 | 3.128225 | -0.84281 | H | -3.00872 | 1.240547 | 1.056586 |
| C | -0.91051 | 2.86021 | -0.02157 | H | -3.85441 | 2.182 | -1.73144 |
| O | -0.15683 | 1.87963 | -0.529 | H | -2.52172 | 1.084903 | -1.4296 |
| C | 4.371303 | 1.954426 | -0.16544 | H | -1.81241 | 3.451361 | -1.83237 |
| O | 2.186036 | 1.354001 | 0.733705 | H | -2.67692 | 3.963802 | -0.38105 |
| O | 4.209485 | -0.49803 | 1.555871 | H | 0.664645 | 1.755195 | 0.017321 |
| C | 4.794962 | -1.01181 | -0.6782 | H | 5.114999 | 1.722342 | -0.92748 |
| O | -0.60294 | 3.471597 | 0.984426 | H | 3.969515 | 2.945049 | -0.38901 |
| H | 2.797572 | 0.898282 | -1.16238 | H | 4.872721 | 1.98434 | 0.804208 |
| H | 1.751366 | -1.12483 | 0.961352 | H | 2.511996 | 2.067467 | 1.29581 |
| H | 2.831282 | -2.44401 | 0.549126 | H | 3.495839 | -0.24589 | 2.156935 |
| H | 1.309527 | -0.70104 | -1.4626 | H | 4.986096 | -2.06772 | -0.47837 |
| H | 2.440192 | -1.96576 | -1.90163 | H | 4.542106 | -0.90418 | -1.73404 |
| H | 1.001693 | -3.67457 | -0.83458 | H | 5.714765 | -0.45859 | -0.48811 |
| H | 0.159995 | -2.7721 | -2.07848 |  |  |  |  |

**Table S5.** NMR calculation of **3a** (14*R*^*^, 15*R*^*^), (A) Boltzmann distribution of energy minimized conformers, (B) Optimized Z-matrixes of **3a** conformers in the MeOH (Ǻ)

(A)

| Conformer | Calculated Energy (G)  (atomic units) | Relative Energy (kcal/mol) | Boltzmann Weights (%) |
| --- | --- | --- | --- |
| 1 | -967.904563 | 0.000000 | 0.000000639 |
| 2 | -967.905095 | -0.333835 | 0.000006922 |
| 3 | -967.905258 | -0.436119 | 0.000014364 |
| 4 | -967.906617 | -1.288905 | 0.006318264 |
| 5 | -967.904461 | 0.064006 | 0.000000405 |
| 6 | -967.904698 | -0.084714 | 0.000001170 |
| 7 | -967.902393 | 1.361696 | 0.000000000 |
| 8 | -967.901988 | 1.615837 | 0.000000000 |
| 9 | -967.908776 | -2.643698 | 99.993604265 |
| 10 | -967.89921 | 3.359058 | 0.000000000 |
| 11 | -967.905476 | -0.572916 | 0.000038132 |
| 12 | -967.9004 | 2.612322 | 0.000000000 |
| 13 | -967.901458 | 1.948417 | 0.000000000 |
| 14 | -967.901729 | 1.778362 | 0.000000000 |
| 15 | -967.905259 | -0.436747 | 0.000014428 |
| 16 | -967.90474 | -0.111069 | 0.000001412 |

(B)

| **Conformer 1** | | | | | | | |
| --- | --- | --- | --- | --- | --- | --- | --- |
| Atom | X | Y | Z | Atom | X | Y | Z |
| C | -4.2156 | -0.33687 | 1.09259 | H | -1.97011 | 1.193873 | 0.980903 |
| C | -4.09489 | 0.94874 | 0.755076 | H | -2.40504 | -3.28961 | 1.142919 |
| C | -3.14082 | -1.29929 | 1.515464 | H | -4.10048 | -3.08178 | 0.77121 |
| C | -2.84434 | 1.780902 | 0.691263 | H | -2.92446 | -3.38085 | -1.31034 |
| C | -3.12048 | -2.59948 | 0.685619 | H | -3.53997 | -1.74387 | -1.24413 |
| C | -2.79647 | -2.41672 | -0.80478 | H | -1.36075 | -1.59344 | -2.17849 |
| C | -1.39528 | -1.87281 | -1.11963 | H | -1.22942 | -0.94453 | -0.56599 |
| C | -0.24941 | -2.85577 | -0.84973 | H | -0.31074 | -3.24292 | 0.173342 |
| C | 1.146994 | -2.25189 | -1.07377 | H | -0.37516 | -3.72167 | -1.50872 |
| C | 1.610409 | -1.37352 | 0.097099 | H | 1.865693 | -3.06235 | -1.21955 |
| C | 2.874238 | -0.54059 | -0.15159 | H | 1.143243 | -1.67019 | -2.00066 |
| C | 3.220168 | 0.310892 | 1.094759 | H | 0.810644 | -0.68474 | 0.384395 |
| O | 2.546306 | 0.412302 | -1.20687 | H | 1.786582 | -2.01743 | 0.962391 |
| C | 4.060859 | -1.38527 | -0.62098 | H | 3.238293 | 1.092011 | -1.15817 |
| O | 4.232123 | 1.248313 | 0.658638 | H | 4.269096 | -2.19682 | 0.078604 |
| C | 3.706737 | -0.45928 | 2.314696 | H | 3.845275 | -1.82249 | -1.5954 |
| C | -2.62587 | 2.398955 | -0.69992 | H | 4.958686 | -0.77203 | -0.71189 |
| C | -1.41869 | 3.350008 | -0.76986 | H | 4.165131 | 2.045534 | 1.19622 |
| C | -0.08777 | 2.675573 | -0.52822 | H | 3.906181 | 0.239061 | 3.130316 |
| O | 0.180794 | 1.725839 | -1.42838 | H | 2.956552 | -1.16999 | 2.665398 |
| O | 0.670725 | 2.960989 | 0.379398 | H | 4.628854 | -1.0009 | 2.098951 |
| H | 2.318139 | 0.875289 | 1.348324 | H | -2.50967 | 1.605412 | -1.44172 |
| H | -5.21837 | -0.76181 | 1.071424 | H | -3.51611 | 2.966897 | -0.98491 |
| H | -5.00562 | 1.480793 | 0.484503 | H | -1.37961 | 3.803928 | -1.76438 |
| H | -2.15878 | -0.82374 | 1.495494 | H | -1.51896 | 4.152083 | -0.03817 |
| H | -3.31925 | -1.57788 | 2.561514 | H | 1.054846 | 1.286706 | -1.23591 |
| H | -2.92307 | 2.596405 | 1.421684 |  |  |  |  |

**Table S5:** (continued)

| **Conformer 2** | | | | | | | |
| --- | --- | --- | --- | --- | --- | --- | --- |
| Atom | X | Y | Z | Atom | X | Y | Z |
| C | -4.64898 | 0.505518 | -0.04704 | H | -3.24696 | -2.03264 | 1.536314 |
| C | -4.50474 | -0.73821 | 0.415464 | H | -4.18329 | 3.079604 | 0.712205 |
| C | -3.56371 | 1.425312 | -0.53315 | H | -2.86834 | 2.141332 | 1.378575 |
| C | -3.2199 | -1.5069 | 0.575093 | H | -2.74631 | 4.146485 | -0.9315 |
| C | -3.2504 | 2.570655 | 0.447209 | H | -2.08323 | 4.365632 | 0.674893 |
| C | -2.2649 | 3.615023 | -0.10278 | H | -1.09431 | 2.368798 | -1.42647 |
| C | -0.91908 | 3.065343 | -0.59957 | H | -0.34214 | 3.892571 | -1.02746 |
| C | -0.06949 | 2.367822 | 0.467343 | H | 0.250327 | 3.10146 | 1.217216 |
| C | 1.153088 | 1.655637 | -0.11995 | H | -0.67321 | 1.628973 | 1.00427 |
| C | 2.018912 | 0.986076 | 0.953059 | H | 0.804707 | 0.906007 | -0.83682 |
| C | 3.021884 | -0.07996 | 0.472302 | H | 1.748153 | 2.374022 | -0.69292 |
| C | 3.906661 | 0.402971 | -0.70062 | H | 2.563389 | 1.751159 | 1.514409 |
| O | 2.292828 | -1.20497 | -0.10915 | H | 1.365699 | 0.491262 | 1.680414 |
| C | 3.843029 | -0.60048 | 1.651995 | H | 1.955748 | -1.78549 | 0.595141 |
| O | 4.717286 | -0.67392 | -1.19195 | H | 4.398243 | 0.207555 | 2.129868 |
| C | 4.838541 | 1.555851 | -0.37059 | H | 3.180571 | -1.03377 | 2.406045 |
| C | -2.9904 | -2.53899 | -0.54304 | H | 4.550636 | -1.3641 | 1.329063 |
| C | -1.79777 | -3.47213 | -0.27845 | H | 4.129967 | -1.43727 | -1.28261 |
| C | -0.46916 | -2.77885 | -0.10649 | H | 5.393852 | 1.840442 | -1.26589 |
| O | -0.1754 | -1.92797 | -1.09609 | H | 4.283416 | 2.429665 | -0.02768 |
| O | 0.286734 | -2.9771 | 0.830187 | H | 5.558346 | 1.275575 | 0.400289 |
| H | 3.227898 | 0.712697 | -1.50296 | H | -3.88091 | -3.16418 | -0.65212 |
| H | -5.65637 | 0.916252 | -0.07853 | H | -2.85336 | -2.02047 | -1.49457 |
| H | -5.40423 | -1.27517 | 0.709404 | H | -1.96499 | -4.06956 | 0.617954 |
| H | -2.65446 | 0.858907 | -0.74036 | H | -1.68838 | -4.1642 | -1.11982 |
| H | -3.87885 | 1.861065 | -1.48934 | H | 0.702634 | -1.51388 | -0.90552 |
| H | -2.36735 | -0.82514 | 0.619651 |  |  |  |  |

**Table S5:** (continued)

| **Conformer 3** | | | | | | | |
| --- | --- | --- | --- | --- | --- | --- | --- |
| Atom | X | Y | Z | Atom | X | Y | Z |
| C | -4.92627 | -0.23368 | -0.44143 | H | -3.77103 | 1.768128 | 1.036424 |
| C | -4.55997 | 0.995539 | -0.81044 | H | -3.9545 | -2.9104 | 1.533421 |
| C | -4.57277 | -0.96654 | 0.824618 | H | -4.88161 | -2.9505 | 0.047983 |
| C | -3.69504 | 1.957064 | -0.03738 | H | -2.67208 | -3.56898 | -0.56753 |
| C | -4.0849 | -2.40816 | 0.568705 | H | -2.89526 | -1.9412 | -1.16935 |
| C | -2.79164 | -2.52753 | -0.24939 | H | -1.65656 | -1.09533 | 0.916986 |
| C | -1.51756 | -2.09103 | 0.482779 | H | -1.33471 | -2.76616 | 1.327337 |
| C | -0.29128 | -2.06339 | -0.43379 | H | -0.13656 | -3.05915 | -0.86616 |
| C | 0.998142 | -1.60606 | 0.25707 | H | -0.49805 | -1.39549 | -1.27837 |
| C | 2.15598 | -1.46699 | -0.73835 | H | 0.813131 | -0.6485 | 0.753801 |
| C | 3.520336 | -1.01332 | -0.18144 | H | 1.257714 | -2.31895 | 1.045597 |
| C | 3.482179 | 0.440718 | 0.346923 | H | 2.317184 | -2.43919 | -1.2208 |
| O | 4.456954 | -0.9477 | -1.28933 | H | 1.862928 | -0.77384 | -1.53099 |
| C | 4.04612 | -1.98785 | 0.874136 | H | 4.324904 | -1.71857 | -1.8546 |
| O | 3.124013 | 1.332489 | -0.73363 | H | 3.476643 | -1.91897 | 1.803344 |
| C | 4.777706 | 0.926749 | 0.987778 | H | 3.967631 | -3.01397 | 0.50707 |
| C | -2.21378 | 1.886614 | -0.44696 | H | 5.093106 | -1.78566 | 1.097209 |
| C | -1.35293 | 2.915886 | 0.306422 | H | 3.598704 | 1.023161 | -1.52057 |
| C | 0.118905 | 2.580859 | 0.316784 | H | 4.673664 | 1.982461 | 1.242494 |
| O | 0.609435 | 2.294819 | -0.89231 | H | 4.996908 | 0.379839 | 1.905737 |
| O | 0.800214 | 2.555093 | 1.325077 | H | 5.622755 | 0.815427 | 0.306022 |
| H | 2.671408 | 0.530507 | 1.069553 | H | -1.84558 | 0.879537 | -0.23876 |
| H | -5.55593 | -0.79492 | -1.13062 | H | -2.11367 | 2.035394 | -1.52447 |
| H | -4.9 | 1.350482 | -1.78088 | H | -1.47547 | 3.907037 | -0.14231 |
| H | -3.83061 | -0.41215 | 1.402237 | H | -1.66449 | 2.991241 | 1.348671 |
| H | -5.46688 | -1.02886 | 1.45781 | H | 1.555164 | 1.990665 | -0.81196 |
| H | -4.0631 | 2.975992 | -0.19895 |  |  |  |  |

**Table S5:** (continued)

| **Conformer 4** | | | | | | | |
| --- | --- | --- | --- | --- | --- | --- | --- |
| Atom | X | Y | Z | Atom | X | Y | Z |
| C | -4.60741 | 1.02023 | -0.36482 | H | -5.24834 | -1.72218 | 1.253329 |
| C | -4.98304 | -0.25683 | -0.25943 | H | -2.0476 | 1.29846 | -1.15628 |
| C | -3.50387 | 1.715868 | 0.380331 | H | -2.70691 | 2.920978 | -1.23778 |
| C | -4.42665 | -1.31783 | 0.651047 | H | -1.40713 | 3.533274 | 0.832219 |
| C | -2.35084 | 2.152544 | -0.54203 | H | -0.82484 | 1.884759 | 0.935286 |
| C | -1.13022 | 2.661652 | 0.226961 | H | 0.207502 | 2.220486 | -1.40461 |
| C | 0.049604 | 3.026886 | -0.68036 | H | -0.21635 | 3.90951 | -1.27214 |
| C | 1.35806 | 3.31171 | 0.073588 | H | 2.07927 | 3.761474 | -0.61828 |
| C | 2.012878 | 2.092081 | 0.745557 | H | 1.162427 | 4.06962 | 0.840344 |
| C | 2.626921 | 1.103013 | -0.25453 | H | 2.79411 | 2.453191 | 1.419453 |
| C | 3.136423 | -0.21428 | 0.345855 | H | 1.283638 | 1.575795 | 1.374413 |
| C | 3.701771 | -1.13719 | -0.76171 | H | 1.896033 | 0.851332 | -1.02833 |
| O | 1.970721 | -0.90446 | 0.887697 | H | 3.459579 | 1.594575 | -0.76423 |
| C | 4.139344 | -0.00165 | 1.482402 | H | 2.26405 | -1.81523 | 1.050459 |
| O | 3.891859 | -2.42508 | -0.13489 | H | 4.972824 | 0.625322 | 1.160715 |
| C | 4.992897 | -0.67362 | -1.42289 | H | 3.650832 | 0.487192 | 2.324524 |
| C | -3.7792 | -2.4971 | -0.10354 | H | 4.541009 | -0.9559 | 1.826333 |
| C | -2.48952 | -2.14124 | -0.84266 | H | 3.865359 | -3.10697 | -0.81548 |
| C | -1.29562 | -1.89285 | 0.053688 | H | 5.28956 | -1.39476 | -2.18753 |
| O | -0.31813 | -1.23529 | -0.58007 | H | 4.867271 | 0.29314 | -1.91312 |
| O | -1.20118 | -2.2641 | 1.206897 | H | 5.803524 | -0.59655 | -0.69692 |
| H | 2.918497 | -1.23453 | -1.52074 | H | -3.57511 | -3.30286 | 0.604968 |
| H | -5.13361 | 1.637248 | -1.09141 | H | -4.49401 | -2.89178 | -0.83157 |
| H | -5.79 | -0.595 | -0.90785 | H | -2.19798 | -2.96197 | -1.50781 |
| H | -3.9079 | 2.602822 | 0.883594 | H | -2.62458 | -1.27011 | -1.48715 |
| H | -3.10385 | 1.071559 | 1.166262 | H | 0.474458 | -1.12106 | 0.011224 |
| H | -3.70716 | -0.89887 | 1.35636 |  |  |  |  |

**Table S5:** (continued)

| **Conformer 5** | | | | | | | |
| --- | --- | --- | --- | --- | --- | --- | --- |
| Atom | X | Y | Z | Atom | X | Y | Z |
| C | 4.374962 | -0.57516 | 0.272127 | H | 2.15389 | 0.762722 | -0.63629 |
| C | 4.283368 | 0.534095 | -0.46477 | H | 4.055241 | -3.24602 | 0.994127 |
| C | 3.291179 | -1.2283 | 1.082397 | H | 2.410037 | -3.1249 | 1.566708 |
| C | 3.055603 | 1.377403 | -0.69337 | H | 3.392587 | -2.90841 | -1.31959 |
| C | 3.103079 | -2.7343 | 0.815677 | H | 2.448356 | -4.1985 | -0.6084 |
| C | 2.603998 | -3.11389 | -0.58803 | H | 1.532102 | -1.34816 | -1.22018 |
| C | 1.321321 | -2.40989 | -1.05713 | H | 1.046428 | -2.80962 | -2.03973 |
| C | 0.122292 | -2.52851 | -0.11205 | H | -0.1501 | -3.58279 | 0.016496 |
| C | -1.09577 | -1.73256 | -0.59617 | H | 0.40027 | -2.16354 | 0.881588 |
| C | -2.18533 | -1.63415 | 0.475864 | H | -0.76166 | -0.72773 | -0.87246 |
| C | -3.45156 | -0.82961 | 0.120272 | H | -1.49213 | -2.18898 | -1.50845 |
| C | -3.13244 | 0.652577 | -0.18376 | H | -2.51244 | -2.64608 | 0.745111 |
| O | -4.28954 | -0.76835 | 1.303493 | H | -1.75093 | -1.19728 | 1.378403 |
| C | -4.2281 | -1.48544 | -1.02335 | H | -4.29988 | -1.63878 | 1.720197 |
| O | -2.45566 | 1.240195 | 0.948453 | H | -3.69726 | -1.3925 | -1.97321 |
| C | -4.34052 | 1.506976 | -0.55312 | H | -4.36556 | -2.54949 | -0.81715 |
| C | 2.92486 | 2.548436 | 0.296915 | H | -5.21226 | -1.03137 | -1.13356 |
| C | 1.782904 | 3.517481 | -0.05394 | H | -2.93072 | 0.961684 | 1.745247 |
| C | 0.412061 | 2.883341 | -0.12175 | H | -4.02088 | 2.543429 | -0.67037 |
| O | 0.052395 | 2.280151 | 1.015854 | H | -4.78283 | 1.183539 | -1.49628 |
| O | -0.30807 | 2.921034 | -1.10145 | H | -5.10674 | 1.464305 | 0.222783 |
| H | -2.40806 | 0.691702 | -0.99693 | H | 2.782561 | 2.156352 | 1.306545 |
| H | 5.348489 | -1.05974 | 0.331327 | H | 3.85568 | 3.122263 | 0.311234 |
| H | 5.189994 | 0.892966 | -0.94693 | H | 1.739461 | 4.308539 | 0.700932 |
| H | 3.552107 | -1.11647 | 2.142482 | H | 1.966369 | 3.989047 | -1.01963 |
| H | 2.343809 | -0.70141 | 0.953784 | H | -0.86186 | 1.899679 | 0.927651 |
| H | 3.091506 | 1.78033 | -1.71114 |  |  |  |  |

**Table S5:** (continued)

| **Conformer 6** | | | | | | | |
| --- | --- | --- | --- | --- | --- | --- | --- |
| Atom | X | Y | Z | Atom | X | Y | Z |
| C | -4.10081 | 1.65156 | -0.40708 | H | -2.94377 | -0.55732 | -1.47082 |
| C | -4.61559 | 0.427128 | -0.54282 | H | -2.13482 | 3.212364 | 0.8852 |
| C | -2.7611 | 2.133177 | -0.89007 | H | -1.69752 | 1.513436 | 0.882192 |
| C | -3.98376 | -0.76281 | -1.21394 | H | -0.04107 | 1.943288 | -0.94297 |
| C | -1.76008 | 2.403307 | 0.247674 | H | -0.40543 | 3.655396 | -0.88267 |
| C | -0.36126 | 2.746146 | -0.27072 | H | 0.395716 | 3.802864 | 1.445826 |
| C | 0.675843 | 2.933368 | 0.841089 | H | 0.634865 | 2.073927 | 1.518753 |
| C | 2.113565 | 3.131735 | 0.335051 | H | 2.123929 | 3.970932 | -0.36935 |
| C | 2.752406 | 1.911291 | -0.35121 | H | 2.749014 | 3.434052 | 1.175052 |
| C | 3.066224 | 0.763406 | 0.617917 | H | 2.097264 | 1.561262 | -1.15387 |
| C | 3.570513 | -0.55035 | -0.0136 | H | 3.675932 | 2.236247 | -0.8376 |
| C | 2.428651 | -1.30455 | -0.74242 | H | 3.840706 | 1.107352 | 1.315197 |
| O | 3.942828 | -1.45895 | 1.056216 | H | 2.187529 | 0.526831 | 1.219946 |
| C | 4.779125 | -0.31761 | -0.9206 | H | 4.406228 | -0.96079 | 1.740217 |
| O | 1.422395 | -1.65992 | 0.228272 | H | 4.49736 | 0.194003 | -1.84321 |
| C | 2.863249 | -2.54694 | -1.51243 | H | 5.518741 | 0.300358 | -0.40568 |
| C | -4.05992 | -2.05838 | -0.3851 | H | 5.250183 | -1.26461 | -1.1818 |
| C | -3.40797 | -1.9898 | 1.000086 | H | 1.895792 | -1.99042 | 1.007605 |
| C | -1.9217 | -1.71277 | 1.064728 | H | 1.978859 | -3.05038 | -1.9061 |
| O | -1.23304 | -2.1534 | 0.008591 | H | 3.505543 | -2.28835 | -2.35534 |
| O | -1.39173 | -1.16908 | 2.015314 | H | 3.400343 | -3.24407 | -0.86651 |
| H | 1.937722 | -0.61168 | -1.42667 | H | -5.11076 | -2.32627 | -0.23696 |
| H | -4.70058 | 2.392935 | 0.117824 | H | -3.61192 | -2.87053 | -0.96163 |
| H | -5.60835 | 0.250282 | -0.13123 | H | -3.88979 | -1.23899 | 1.627594 |
| H | -2.32077 | 1.411014 | -1.58122 | H | -3.55054 | -2.95002 | 1.509483 |
| H | -2.90094 | 3.058251 | -1.46252 | H | -0.26567 | -1.9547 | 0.126844 |
| H | -4.5018 | -0.95339 | -2.16215 |  |  |  |  |

**Table S5:** (continued)

| **Conformer 7** | | | | | | | |
| --- | --- | --- | --- | --- | --- | --- | --- |
| Atom | X | Y | Z | Atom | X | Y | Z |
| C | 4.537076 | 0.492696 | -0.15456 | H | 2.826728 | -0.91242 | 1.447612 |
| C | 4.582901 | -0.79241 | 0.203544 | H | 3.443429 | 2.370312 | -1.73688 |
| C | 3.539897 | 1.541807 | 0.251542 | H | 2.139887 | 1.266301 | -1.3665 |
| C | 3.657045 | -1.54205 | 1.122257 | H | 2.478504 | 4.128219 | -0.33043 |
| C | 2.74005 | 2.080258 | -0.94864 | H | 1.354792 | 3.599007 | -1.56551 |
| C | 1.844148 | 3.287873 | -0.63542 | H | 1.240715 | 2.91527 | 1.41477 |
| C | 0.767135 | 3.072642 | 0.439032 | H | 0.191462 | 4.000724 | 0.534432 |
| C | -0.19371 | 1.913054 | 0.158895 | H | -0.5959 | 2.004962 | -0.85429 |
| C | -1.33863 | 1.841399 | 1.174946 | H | 0.358409 | 0.969793 | 0.179721 |
| C | -2.18667 | 0.55874 | 1.164834 | H | -0.90992 | 1.921213 | 2.180618 |
| C | -3.04073 | 0.254977 | -0.07588 | H | -1.98303 | 2.719233 | 1.061662 |
| C | -3.95208 | -0.97052 | 0.199573 | H | -1.53739 | -0.30491 | 1.335895 |
| O | -2.1402 | -0.15827 | -1.1444 | H | -2.86395 | 0.612317 | 2.020561 |
| C | -3.8411 | 1.463876 | -0.56517 | H | -2.70082 | -0.64411 | -1.77171 |
| O | -4.45979 | -1.37641 | -1.09253 | H | -4.44736 | 1.889237 | 0.236733 |
| C | -5.10741 | -0.74765 | 1.166198 | H | -3.16409 | 2.237243 | -0.92676 |
| C | 3.112889 | -2.84533 | 0.50538 | H | -4.50481 | 1.181117 | -1.38388 |
| C | 2.180898 | -2.63559 | -0.68888 | H | -4.68267 | -2.31337 | -1.05695 |
| C | 0.831261 | -2.05555 | -0.32624 | H | -5.66151 | -1.68052 | 1.291182 |
| O | 0.263577 | -1.41059 | -1.35044 | H | -4.7564 | -0.43989 | 2.152269 |
| O | 0.297022 | -2.17245 | 0.758823 | H | -5.79736 | 0.007721 | 0.787732 |
| H | -3.30224 | -1.76529 | 0.580209 | H | 3.95424 | -3.46383 | 0.179186 |
| H | 5.30935 | 0.843996 | -0.83731 | H | 2.585656 | -3.4122 | 1.275546 |
| H | 5.392913 | -1.39347 | -0.20785 | H | 2.640499 | -2.00203 | -1.44944 |
| H | 2.860611 | 1.157962 | 1.014498 | H | 1.975129 | -3.59582 | -1.17557 |
| H | 4.080483 | 2.378923 | 0.712376 | H | -0.61786 | -1.03005 | -1.09196 |
| H | 4.209711 | -1.81337 | 2.03 |  |  |  |  |

**Table S5:** (continued)

| **Conformer 8** | | | | | | | |
| --- | --- | --- | --- | --- | --- | --- | --- |
| Atom | X | Y | Z | Atom | X | Y | Z |
| C | -3.8294 | 1.495337 | 0.1906 | H | -3.19475 | -0.58797 | -1.45593 |
| C | -4.60432 | 0.49829 | -0.24347 | H | -2.41362 | 3.206862 | -1.44232 |
| C | -2.3421 | 1.636325 | 0.032102 | H | -2.2302 | 3.792098 | 0.200693 |
| C | -4.18259 | -0.72528 | -1.01191 | H | -0.07467 | 2.324229 | -1.40126 |
| C | -1.90078 | 3.011081 | -0.49481 | H | -0.17387 | 4.064355 | -1.23596 |
| C | -0.38584 | 3.122223 | -0.7179 | H | 0.156102 | 3.908076 | 1.20658 |
| C | 0.457576 | 3.074056 | 0.562697 | H | 0.235645 | 2.164293 | 1.127978 |
| C | 1.971306 | 3.166726 | 0.313169 | H | 2.161943 | 4.01533 | -0.35334 |
| C | 2.626986 | 1.910141 | -0.28776 | H | 2.476406 | 3.404035 | 1.25605 |
| C | 2.780153 | 0.762177 | 0.720395 | H | 2.054345 | 1.581035 | -1.16001 |
| C | 3.448012 | -0.53499 | 0.205889 | H | 3.614686 | 2.190226 | -0.66341 |
| C | 2.539655 | -1.28215 | -0.80066 | H | 3.394919 | 1.110729 | 1.55626 |
| O | 3.689292 | -1.3985 | 1.337277 | H | 1.805649 | 0.502042 | 1.140888 |
| C | 4.824294 | -0.24775 | -0.38886 | H | 2.832556 | -1.74563 | 1.620683 |
| O | 1.377277 | -1.70898 | -0.05165 | H | 4.74792 | 0.270562 | -1.34558 |
| C | 3.17648 | -2.48882 | -1.47803 | H | 5.397895 | 0.381395 | 0.294737 |
| C | -4.1864 | -2.02095 | -0.1727 | H | 5.37642 | -1.17451 | -0.54233 |
| C | -3.17923 | -2.02775 | 0.980205 | H | 0.745067 | -2.15872 | -0.63599 |
| C | -1.73612 | -2.00757 | 0.538517 | H | 2.428998 | -3.01065 | -2.08051 |
| O | -0.93059 | -1.40633 | 1.423676 | H | 3.981373 | -2.18522 | -2.14854 |
| O | -1.32001 | -2.49478 | -0.49755 | H | 3.575601 | -3.18705 | -0.74085 |
| H | 2.218294 | -0.56823 | -1.56476 | H | -5.18306 | -2.17672 | 0.249776 |
| H | -4.31097 | 2.299432 | 0.744956 | H | -3.98718 | -2.86894 | -0.83164 |
| H | -5.66549 | 0.548891 | -0.00653 | H | -3.34617 | -1.19235 | 1.661031 |
| H | -1.87763 | 1.465239 | 1.009644 | H | -3.30217 | -2.94055 | 1.574078 |
| H | -1.94803 | 0.861691 | -0.63001 | H | -0.01318 | -1.40106 | 1.062986 |
| H | -4.87672 | -0.87078 | -1.8463 |  |  |  |  |

**Table S5:** (continued)

| **Conformer 9** | | | | | | | |
| --- | --- | --- | --- | --- | --- | --- | --- |
| Atom | X | Y | Z | Atom | X | Y | Z |
| C | 4.352595 | 1.231711 | -0.05967 | H | 3.44786 | -0.8664 | 1.432404 |
| C | 4.792217 | -0.02833 | -0.02882 | H | 2.510948 | 3.111702 | -0.85054 |
| C | 3.24669 | 1.850549 | 0.747683 | H | 1.683857 | 1.573021 | -0.70711 |
| C | 4.307467 | -1.16422 | 0.830037 | H | 0.609572 | 2.365693 | 1.428715 |
| C | 2.103645 | 2.398217 | -0.12419 | H | 1.455304 | 3.887952 | 1.283983 |
| C | 1.005176 | 3.078936 | 0.697541 | H | -0.82791 | 4.195873 | 0.544498 |
| C | -0.14594 | 3.668208 | -0.13219 | H | 0.261053 | 4.430395 | -0.80651 |
| C | -0.95707 | 2.664117 | -0.9651 | H | -1.69993 | 3.221091 | -1.54741 |
| C | -1.67501 | 1.58763 | -0.14359 | H | -0.30585 | 2.178548 | -1.69968 |
| C | -2.49517 | 0.643616 | -1.02918 | H | -0.94669 | 1.002882 | 0.422602 |
| C | -3.07154 | -0.61288 | -0.36007 | H | -2.32053 | 2.07807 | 0.592809 |
| C | -3.93413 | -0.29636 | 0.881507 | H | -3.31737 | 1.192891 | -1.49606 |
| O | -1.93631 | -1.38 | 0.127153 | H | -1.86404 | 0.295004 | -1.85368 |
| C | -3.82303 | -1.46016 | -1.39109 | H | -2.2876 | -2.02808 | 0.756539 |
| O | -4.21688 | -1.57537 | 1.493011 | H | -4.64506 | -0.90142 | -1.84154 |
| C | -5.2211 | 0.469449 | 0.608752 | H | -3.13598 | -1.75299 | -2.18674 |
| C | 3.961965 | -2.43175 | 0.025058 | H | -4.22993 | -2.36327 | -0.93453 |
| C | 2.769329 | -2.27716 | -0.91774 | H | -4.41477 | -1.43304 | 2.425584 |
| C | 1.427953 | -2.17099 | -0.22651 | H | -5.74211 | 0.65763 | 1.549959 |
| O | 0.477955 | -1.70055 | -1.04159 | H | -5.01683 | 1.436359 | 0.144924 |
| O | 1.209074 | -2.49435 | 0.924316 | H | -5.88911 | -0.09634 | -0.042 |
| H | -3.30853 | 0.276583 | 1.570678 | H | 4.832046 | -2.72342 | -0.57078 |
| H | 4.841987 | 1.911668 | -0.75543 | H | 3.767909 | -3.25246 | 0.718938 |
| H | 5.61154 | -0.28586 | -0.69892 | H | 2.886276 | -1.41048 | -1.57218 |
| H | 2.840649 | 1.137035 | 1.468371 | H | 2.698476 | -3.14544 | -1.58242 |
| H | 3.661642 | 2.679511 | 1.335579 | H | -0.3938 | -1.64391 | -0.56586 |
| H | 5.101909 | -1.43018 | 1.538046 |  |  |  |  |

**Table S5:** (continued)

| **Conformer 10** | | | | | | | |
| --- | --- | --- | --- | --- | --- | --- | --- |
| Atom | X | Y | Z | Atom | X | Y | Z |
| C | 2.594689 | 0.212935 | 1.66524 | H | 4.027646 | -2.50405 | 0.61414 |
| C | 2.610806 | -1.03364 | 1.188566 | H | 1.804724 | 2.741432 | 1.398422 |
| C | 3.474439 | 1.380094 | 1.312051 | H | 3.278501 | 3.478114 | 0.806462 |
| C | 3.539776 | -1.62864 | 0.167285 | H | 3.093568 | 2.428202 | -1.35146 |
| C | 2.66937 | 2.570098 | 0.748847 | H | 1.838754 | 1.339545 | -0.81651 |
| C | 2.215401 | 2.359499 | -0.69994 | H | 1.523237 | 4.377156 | -1.05081 |
| C | 1.155587 | 3.35473 | -1.19608 | H | 1.039672 | 3.22526 | -2.27815 |
| C | -0.23157 | 3.227903 | -0.54384 | H | -0.17523 | 3.483389 | 0.519877 |
| C | -0.86212 | 1.839077 | -0.69584 | H | -0.89318 | 3.975165 | -0.99635 |
| C | -2.31782 | 1.78802 | -0.22143 | H | -0.81535 | 1.542244 | -1.74859 |
| C | -2.97558 | 0.391028 | -0.20202 | H | -0.26139 | 1.111135 | -0.14655 |
| C | -2.40768 | -0.48834 | 0.950924 | H | -2.39308 | 2.217411 | 0.782946 |
| O | -2.72099 | -0.31637 | -1.43857 | H | -2.9224 | 2.429453 | -0.87054 |
| C | -4.49543 | 0.527141 | -0.13284 | H | -1.77988 | -0.56892 | -1.47801 |
| O | -2.68141 | -1.88725 | 0.664759 | H | -4.79574 | 1.162281 | 0.701037 |
| C | -2.92445 | -0.17955 | 2.345604 | H | -4.85534 | 0.984248 | -1.0559 |
| C | 2.865394 | -2.08118 | -1.14448 | H | -4.98133 | -0.44409 | -0.01994 |
| C | 1.731109 | -3.10743 | -0.97303 | H | -3.00152 | -1.90711 | -0.25416 |
| C | 0.376601 | -2.49583 | -0.69792 | H | -2.42946 | -0.82883 | 3.070149 |
| O | -0.29333 | -3.09436 | 0.285062 | H | -2.70948 | 0.853671 | 2.623565 |
| O | -0.07639 | -1.56212 | -1.34036 | H | -3.99972 | -0.34601 | 2.417265 |
| H | -1.32001 | -0.37407 | 0.947276 | H | 2.478649 | -1.21455 | -1.68534 |
| H | 1.837602 | 0.442091 | 2.414053 | H | 3.635138 | -2.52868 | -1.77762 |
| H | 1.873 | -1.721 | 1.596912 | H | 1.608559 | -3.66049 | -1.91002 |
| H | 3.987672 | 1.709913 | 2.223119 | H | 1.967286 | -3.84037 | -0.20065 |
| H | 4.254576 | 1.094499 | 0.601943 | H | -1.18743 | -2.66409 | 0.414651 |
| H | 4.337766 | -0.92771 | -0.08502 |  |  |  |  |

**Table S5:** (continued)

| **Conformer 11** | | | | | | | |
| --- | --- | --- | --- | --- | --- | --- | --- |
| Atom | X | Y | Z | Atom | X | Y | Z |
| C | -4.70233 | 0.748631 | -0.19997 | H | -4.15028 | -2.01111 | 1.214958 |
| C | -4.67756 | -0.58243 | -0.29323 | H | -2.08859 | 1.488805 | -0.74597 |
| C | -3.76644 | 1.614301 | 0.599845 | H | -3.1106 | 2.884022 | -1.03223 |
| C | -3.68764 | -1.51261 | 0.353338 | H | -2.32259 | 3.919158 | 1.101976 |
| C | -2.65843 | 2.274692 | -0.241 | H | -1.31082 | 2.530162 | 1.418563 |
| C | -1.72679 | 3.141799 | 0.610334 | H | -1.01641 | 4.480143 | -0.92411 |
| C | -0.5865 | 3.823113 | -0.15937 | H | -0.04777 | 4.478581 | 0.534553 |
| C | 0.430675 | 2.889484 | -0.83361 | H | -0.0529 | 2.32007 | -1.63464 |
| C | 1.13687 | 1.914962 | 0.117511 | H | 1.185242 | 3.513652 | -1.32584 |
| C | 2.325415 | 1.21957 | -0.55645 | H | 1.483883 | 2.462524 | 0.999625 |
| C | 2.951062 | 0.043465 | 0.210201 | H | 0.427078 | 1.164961 | 0.47669 |
| C | 4.17242 | -0.52165 | -0.57018 | H | 2.015648 | 0.84746 | -1.54122 |
| O | 1.993515 | -1.05515 | 0.28086 | H | 3.102836 | 1.963612 | -0.74965 |
| C | 3.29337 | 0.379228 | 1.657328 | H | 1.532228 | -1.16831 | -0.56877 |
| O | 4.570467 | -1.78202 | -0.01857 | H | 3.933139 | 1.260876 | 1.710453 |
| C | 5.409226 | 0.361596 | -0.59557 | H | 2.384409 | 0.583465 | 2.222756 |
| C | -3.18335 | -2.59576 | -0.61441 | H | 3.813776 | -0.45435 | 2.130317 |
| C | -2.00855 | -3.41039 | -0.04565 | H | 3.752916 | -2.27102 | 0.154182 |
| C | -0.72731 | -2.61222 | -0.04132 | H | 6.187415 | -0.125 | -1.18637 |
| O | -0.20385 | -2.42349 | 1.175209 | H | 5.201411 | 1.330393 | -1.05033 |
| O | -0.21124 | -2.17002 | -1.05342 | H | 5.798935 | 0.522577 | 0.41089 |
| H | 3.832704 | -0.6837 | -1.60318 | H | -4.0007 | -3.2816 | -0.85116 |
| H | -5.46951 | 1.278639 | -0.76054 | H | -2.87188 | -2.1406 | -1.55794 |
| H | -5.43573 | -1.06382 | -0.90842 | H | -2.22905 | -3.76589 | 0.961308 |
| H | -4.34943 | 2.402059 | 1.09056 | H | -1.82891 | -4.28185 | -0.68046 |
| H | -3.30063 | 1.036416 | 1.402048 | H | 0.619339 | -1.88345 | 1.084845 |
| H | -2.84119 | -0.94618 | 0.747534 |  |  |  |  |

**Table S5:** (continued)

| **Conformer 12** | | | | | | | |
| --- | --- | --- | --- | --- | --- | --- | --- |
| Atom | X | Y | Z | Atom | X | Y | Z |
| C | 4.371927 | 0.748782 | -0.47418 | H | 2.850884 | -0.46116 | 1.429865 |
| C | 4.535331 | -0.44941 | 0.08992 | H | 2.537544 | 1.74858 | -2.13306 |
| C | 3.348624 | 1.79933 | -0.13258 | H | 1.574525 | 0.890663 | -0.95655 |
| C | 3.732528 | -1.06405 | 1.204452 | H | 1.813197 | 3.918255 | -1.32709 |
| C | 2.155614 | 1.8052 | -1.10796 | H | 0.433597 | 2.925228 | -1.73201 |
| C | 1.245248 | 3.037609 | -1.00561 | H | 1.461461 | 3.613938 | 1.066183 |
| C | 0.653014 | 3.340269 | 0.380245 | H | 0.025714 | 4.234649 | 0.289748 |
| C | -0.17655 | 2.225858 | 1.033468 | H | 0.459798 | 1.361267 | 1.250471 |
| C | -1.39041 | 1.755263 | 0.222394 | H | -0.52153 | 2.592278 | 2.007279 |
| C | -2.31221 | 0.869696 | 1.072792 | H | -1.94244 | 2.632349 | -0.13302 |
| C | -3.52505 | 0.185016 | 0.381081 | H | -1.05402 | 1.220279 | -0.66593 |
| C | -3.13619 | -1.18378 | -0.22728 | H | -1.71707 | 0.085441 | 1.550154 |
| O | -4.0014 | 0.952234 | -0.74845 | H | -2.69835 | 1.48907 | 1.889098 |
| C | -4.65395 | 0.021001 | 1.403636 | H | -4.09317 | 1.875697 | -0.48604 |
| O | -2.14048 | -0.99803 | -1.25599 | H | -4.34167 | -0.62334 | 2.229832 |
| C | -4.29189 | -1.99967 | -0.79764 | H | -4.91486 | 0.995696 | 1.821765 |
| C | 3.310928 | -2.51689 | 0.911235 | H | -5.54751 | -0.4039 | 0.948187 |
| C | 2.404564 | -2.68441 | -0.30932 | H | -2.43127 | -0.25997 | -1.81071 |
| C | 0.997901 | -2.15758 | -0.13053 | H | -3.88948 | -2.89019 | -1.28298 |
| O | 0.420409 | -1.85528 | -1.29849 | H | -4.9781 | -2.32559 | -0.0153 |
| O | 0.428369 | -2.04563 | 0.936812 | H | -4.85375 | -1.42555 | -1.53668 |
| H | -2.63321 | -1.76089 | 0.550055 | H | 4.209384 | -3.1199 | 0.749424 |
| H | 5.041887 | 1.009275 | -1.29143 | H | 2.81144 | -2.92846 | 1.790826 |
| H | 5.338637 | -1.07721 | -0.29353 | H | 2.838892 | -2.2215 | -1.19703 |
| H | 2.990004 | 1.670665 | 0.890491 | H | 2.29477 | -3.74904 | -0.54594 |
| H | 3.836104 | 2.780787 | -0.16704 | H | -0.51227 | -1.5401 | -1.16332 |
| H | 4.339057 | -1.07716 | 2.118482 |  |  |  |  |

**Table S5:** (continued)

| **Conformer 13** | | | | | | | |
| --- | --- | --- | --- | --- | --- | --- | --- |
| Atom | X | Y | Z | Atom | X | Y | Z |
| C | 4.534836 | 0.712868 | 0.58427 | H | 4.530068 | -1.34543 | -1.79074 |
| C | 4.570758 | -0.56279 | 0.193664 | H | 1.788446 | 1.135295 | 0.481939 |
| C | 3.74898 | 1.82997 | -0.05077 | H | 2.488882 | 2.212889 | 1.672968 |
| C | 3.830447 | -1.18459 | -0.96072 | H | 2.108021 | 4.167913 | 0.284565 |
| C | 2.369952 | 2.053681 | 0.595175 | H | 1.685248 | 3.192489 | -1.10712 |
| C | 1.61082 | 3.238608 | -0.01361 | H | 0.030391 | 3.192642 | 1.464112 |
| C | 0.127047 | 3.302004 | 0.376706 | H | -0.26583 | 4.295996 | 0.135374 |
| C | -0.738 | 2.249169 | -0.32637 | H | -0.6669 | 2.400273 | -1.41007 |
| C | -2.20877 | 2.304655 | 0.093326 | H | -0.33418 | 1.254553 | -0.13181 |
| C | -3.18274 | 1.436925 | -0.72155 | H | -2.29251 | 2.076386 | 1.160734 |
| C | -3.13159 | -0.10016 | -0.61562 | H | -2.55527 | 3.339359 | -0.0097 |
| C | -3.04438 | -0.61679 | 0.843808 | H | -4.1991 | 1.735489 | -0.45126 |
| O | -1.91575 | -0.55723 | -1.3036 | H | -3.07193 | 1.6799 | -1.78316 |
| C | -4.3239 | -0.69409 | -1.36647 | H | -2.13276 | -1.3906 | -1.74072 |
| O | -2.91668 | -2.04057 | 0.866 | H | -5.26832 | -0.36355 | -0.93406 |
| C | -4.23973 | -0.24723 | 1.713056 | H | -4.30214 | -0.37417 | -2.41039 |
| C | 3.221276 | -2.55587 | -0.61353 | H | -4.29965 | -1.78513 | -1.32883 |
| C | 2.20483 | -2.57637 | 0.541539 | H | -1.97373 | -2.26635 | 0.763413 |
| C | 0.783592 | -2.17659 | 0.232711 | H | -4.0578 | -0.57859 | 2.73657 |
| O | 0.656979 | -1.1973 | -0.65682 | H | -4.41259 | 0.829431 | 1.730685 |
| O | -0.17644 | -2.6968 | 0.782513 | H | -5.14804 | -0.73695 | 1.356907 |
| H | -2.14179 | -0.17985 | 1.283597 | H | 2.768325 | -2.98681 | -1.51013 |
| H | 5.122254 | 0.987087 | 1.457952 | H | 4.035345 | -3.22957 | -0.33016 |
| H | 5.200718 | -1.2463 | 0.761363 | H | 2.1465 | -3.57282 | 0.979209 |
| H | 4.330561 | 2.755776 | 0.020372 | H | 2.541713 | -1.90837 | 1.343444 |
| H | 3.606216 | 1.638833 | -1.11779 | H | -0.30156 | -0.96053 | -0.78331 |
| H | 3.055836 | -0.51465 | -1.33253 |  |  |  |  |

**Table S5:** (continued)

| **Conformer 14** | | | | | | | |
| --- | --- | --- | --- | --- | --- | --- | --- |
| Atom | X | Y | Z | Atom | X | Y | Z |
| C | 4.64683 | 0.753412 | -0.20129 | H | 3.972102 | -2.19004 | -1.25115 |
| C | 4.592602 | -0.56547 | -0.00338 | H | 3.354909 | 3.144678 | 0.485622 |
| C | 3.700589 | 1.602978 | -1.00651 | H | 2.218113 | 3.145778 | -0.84857 |
| C | 3.539973 | -1.51876 | -0.49784 | H | 1.288363 | 0.933659 | 0.063133 |
| C | 2.761167 | 2.489031 | -0.16235 | H | 2.327866 | 1.132123 | 1.450033 |
| C | 1.771323 | 1.689464 | 0.690979 | H | 0.18162 | 1.904845 | 2.12302 |
| C | 0.697376 | 2.534177 | 1.390897 | H | 1.186083 | 3.324591 | 1.972355 |
| C | -0.34185 | 3.188705 | 0.46444 | H | -1.03455 | 3.770053 | 1.083929 |
| C | -1.1588 | 2.234317 | -0.42336 | H | 0.156068 | 3.915327 | -0.18522 |
| C | -2.0103 | 1.234916 | 0.369067 | H | -1.80647 | 2.846978 | -1.05589 |
| C | -2.9203 | 0.32338 | -0.46634 | H | -0.49598 | 1.693741 | -1.10355 |
| C | -3.60862 | -0.72754 | 0.438442 | H | -1.36357 | 0.594156 | 0.974719 |
| O | -2.05966 | -0.43182 | -1.36962 | H | -2.64151 | 1.790501 | 1.068322 |
| C | -3.93047 | 1.104801 | -1.31071 | H | -2.62098 | -1.14563 | -1.71279 |
| O | -4.23533 | -1.66963 | -0.46335 | H | -4.52167 | 1.78247 | -0.69222 |
| C | -4.62935 | -0.18776 | 1.431069 | H | -3.41179 | 1.696249 | -2.06432 |
| C | 2.96844 | -2.38432 | 0.638573 | H | -4.61329 | 0.424557 | -1.82183 |
| C | 1.751424 | -3.24198 | 0.224889 | H | -4.30423 | -2.52132 | -0.01764 |
| C | 0.473845 | -2.43582 | 0.190076 | H | -5.04258 | -1.01393 | 2.013829 |
| O | 0.239407 | -1.85231 | -0.9899 | H | -4.17325 | 0.513543 | 2.131921 |
| O | -0.26399 | -2.30269 | 1.148002 | H | -5.45396 | 0.31242 | 0.920895 |
| H | -2.80779 | -1.24439 | 0.974983 | H | 3.749142 | -3.05738 | 1.002818 |
| H | 5.466579 | 1.295813 | 0.266728 | H | 2.689303 | -1.75145 | 1.485936 |
| H | 5.378262 | -1.01724 | 0.600295 | H | 1.929178 | -3.69875 | -0.75079 |
| H | 4.295566 | 2.255233 | -1.65554 | H | 1.602648 | -4.03567 | 0.957053 |
| H | 3.093525 | 0.982577 | -1.67025 | H | -0.59745 | -1.31463 | -0.95483 |
| H | 2.733036 | -0.97852 | -0.99207 |  |  |  |  |

**Table S5:** (continued)

| **Conformer 15** | | | | | | | |
| --- | --- | --- | --- | --- | --- | --- | --- |
| Atom | X | Y | Z | Atom | X | Y | Z |
| C | 4.516312 | -0.40432 | 0.154292 | H | 3.436746 | 2.310471 | 1.102596 |
| C | 4.23491 | 0.841331 | -0.23196 | H | 4.494398 | -3.12005 | 0.3867 |
| C | 3.801106 | -1.24176 | 1.178021 | H | 3.229322 | -3.28102 | 1.587165 |
| C | 3.103975 | 1.717251 | 0.240465 | H | 2.774088 | -2.21965 | -1.24797 |
| C | 3.543619 | -2.68977 | 0.720184 | H | 2.543183 | -3.8857 | -0.75823 |
| C | 2.502474 | -2.85254 | -0.39522 | H | 0.819124 | -3.14077 | 0.919768 |
| C | 1.064475 | -2.54169 | 0.033792 | H | 0.990385 | -1.49519 | 0.342367 |
| C | 0.035712 | -2.83553 | -1.06299 | H | 0.270017 | -2.24175 | -1.95464 |
| C | -1.42664 | -2.58349 | -0.66041 | H | 0.136682 | -3.8846 | -1.36152 |
| C | -1.76916 | -1.09526 | -0.51792 | H | -2.07455 | -3.04201 | -1.41156 |
| C | -3.19853 | -0.7598 | -0.04117 | H | -1.61706 | -3.11896 | 0.278698 |
| C | -3.32652 | 0.774198 | 0.152086 | H | -1.0692 | -0.62402 | 0.173587 |
| O | -3.42897 | -1.29065 | 1.28833 | H | -1.6227 | -0.61027 | -1.48832 |
| C | -4.26035 | -1.29437 | -1.00333 | H | -3.14637 | -2.21222 | 1.313873 |
| O | -2.45789 | 1.187223 | 1.231741 | H | -4.17765 | -0.814 | -1.98162 |
| C | -4.73582 | 1.285292 | 0.427848 | H | -4.1425 | -2.36915 | -1.14693 |
| C | 2.623387 | 2.685652 | -0.84879 | H | -5.26177 | -1.1186 | -0.61283 |
| C | 1.477002 | 3.614885 | -0.4026 | H | -2.51329 | 0.497475 | 1.911657 |
| C | 0.162135 | 2.880558 | -0.28288 | H | -4.68839 | 2.352441 | 0.650112 |
| O | -0.16675 | 2.568368 | 0.973107 | H | -5.38531 | 1.152083 | -0.43818 |
| O | -0.52769 | 2.581308 | -1.23969 | H | -5.18383 | 0.773566 | 1.281913 |
| H | -2.92689 | 1.262302 | -0.73806 | H | 3.462452 | 3.314849 | -1.15885 |
| H | 5.389629 | -0.87885 | -0.29108 | H | 2.309765 | 2.128013 | -1.73618 |
| H | 4.902453 | 1.301784 | -0.95831 | H | 1.725911 | 4.089942 | 0.54763 |
| H | 4.428399 | -1.28427 | 2.077823 | H | 1.337003 | 4.394801 | -1.15277 |
| H | 2.86825 | -0.76694 | 1.489512 | H | -1.01794 | 2.048333 | 0.988915 |
| H | 2.271488 | 1.10803 | 0.600259 |  |  |  |  |

**Table S5:** (continued)

| **Conformer 16** | | | | | | | |
| --- | --- | --- | --- | --- | --- | --- | --- |
| Atom | X | Y | Z | Atom | X | Y | Z |
| C | 4.670855 | -0.774269 | 0.151694 | H | 3.810058 | 1.949674 | 1.335405 |
| C | 4.496998 | 0.523206 | -0.107378 | H | 4.204275 | -3.5089 | 0.051235 |
| C | 3.877238 | -1.643113 | 1.086806 | H | 2.918144 | -3.56243 | 1.2401 |
| C | 3.431197 | 1.434659 | 0.442777 | H | 2.764487 | -2.22493 | -1.50917 |
| C | 3.353159 | -2.943848 | 0.44735 | H | 2.045702 | -3.75431 | -1.04848 |
| C | 2.314196 | -2.76239 | -0.66706 | H | 0.67299 | -2.47931 | 0.698856 |
| C | 1.03852 | -2.033211 | -0.232412 | H | 1.27344 | -0.98981 | 0.000593 |
| C | -0.065743 | -2.063757 | -1.295791 | H | 0.377044 | -1.82963 | -2.27043 |
| C | -1.220798 | -1.0798 | -1.053222 | H | -0.4599 | -3.08377 | -1.3796 |
| C | -1.983519 | -1.330514 | 0.252606 | H | -0.82508 | -0.06046 | -1.05956 |
| C | -3.066265 | -0.311431 | 0.638612 | H | -1.90644 | -1.14723 | -1.90313 |
| C | -4.076771 | -0.034671 | -0.496364 | H | -2.44534 | -2.32186 | 0.220771 |
| O | -2.391986 | 0.952919 | 0.898813 | H | -1.28114 | -1.35419 | 1.090177 |
| C | -3.755041 | -0.749447 | 1.93511 | H | -3.08204 | 1.633564 | 0.858231 |
| O | -4.885328 | 1.072317 | -0.035692 | H | -4.23596 | -1.7225 | 1.822677 |
| C | -4.951891 | -1.214114 | -0.896879 | H | -3.01032 | -0.82807 | 2.728824 |
| C | 2.996464 | 2.500661 | -0.57324 | H | -4.51097 | -0.02479 | 2.240173 |
| C | 1.82717 | 3.385105 | -0.091184 | H | -5.25255 | 1.524492 | -0.80373 |
| C | 0.495005 | 2.682559 | -0.213 | H | -5.60877 | -0.92183 | -1.71897 |
| O | 0.065373 | 2.138211 | 0.930802 | H | -4.34888 | -2.0576 | -1.23872 |
| O | -0.124072 | 2.598267 | -1.256166 | H | -5.57541 | -1.54529 | -0.06542 |
| H | -3.504293 | 0.30527 | -1.362706 | H | 3.847207 | 3.152553 | -0.78952 |
| H | 5.500906 | -1.275743 | -0.343958 | H | 2.721821 | 2.028318 | -1.521 |
| H | 5.203623 | 0.997292 | -0.786577 | H | 1.99196 | 3.699468 | 0.94071 |
| H | 4.531193 | -1.926773 | 1.921209 | H | 1.768858 | 4.27557 | -0.71849 |
| H | 3.052686 | -1.084016 | 1.532778 | H | -0.80007 | 1.672429 | 0.790108 |
| H | 2.566236 | 0.857343 | 0.774995 |  |  |  |  |

**Table S6.** NMR calculation of **3b** (14*S*^*^, 15*R*^*^), (A) Boltzmann distribution of energy minimized conformers, (B) Optimized Z-matrixes of **3b** conformers in the MeOH (Ǻ)

(A)

| Conformer | Calculated Energy (G)  (atomic units) | Relative Energy (kcal/mol) | Boltzmann Weights (%) |
| --- | --- | --- | --- |
| 1 | -967.906072 | 0.000000 | 0.053204645 |
| 2 | -967.905415 | 0.412274 | 0.002805745 |
| 3 | -967.905047 | 0.643197 | 0.000539843 |
| 4 | -967.901435 | 2.909762 | 0.000000000 |
| 5 | -967.905104 | 0.607429 | 0.000696845 |
| 6 | -967.901901 | 2.617342 | 0.000000000 |
| 7 | -967.907755 | -1.056098 | 99.879795998 |
| 8 | -967.90535 | 0.453062 | 0.002097102 |
| 9 | -967.903558 | 1.577559 | 0.000000686 |
| 10 | -967.903916 | 1.352910 | 0.000003407 |
| 11 | -967.906102 | -0.018825 | 0.060855693 |
| 12 | -967.902898 | 1.991715 | 0.000000036 |
| 13 | -967.900876 | 3.260539 | 0.000000000 |

(B)

| **Conformer 1** | | | | | | | |
| --- | --- | --- | --- | --- | --- | --- | --- |
| Atom | X | Y | Z | Atom | X | Y | Z |
| C | 4.232415 | -1.54851 | 0.473352 | H | 4.501074 | 1.598991 | 0.96165 |
| C | 4.517056 | -0.32333 | 0.026678 | H | 2.544151 | -3.76368 | 0.449236 |
| C | 3.177694 | -1.95457 | 1.46569 | H | 1.453096 | -3.21721 | 1.708592 |
| C | 3.82494 | 0.965983 | 0.372544 | H | 0.844079 | -1.23458 | 0.239084 |
| C | 2.08229 | -2.86855 | 0.882162 | H | 1.808108 | -1.93288 | -1.04444 |
| C | 1.20167 | -2.18631 | -0.16788 | H | 0.366161 | -4.03421 | -0.8943 |
| C | 0.007178 | -3.03996 | -0.60436 | H | -0.65186 | -3.20164 | 0.255817 |
| C | -0.79164 | -2.46093 | -1.78284 | H | -0.13979 | -2.43897 | -2.66355 |
| C | -1.37028 | -1.05006 | -1.58544 | H | -1.60789 | -3.15042 | -2.02784 |
| C | -2.43535 | -0.96456 | -0.48673 | H | -0.55957 | -0.3505 | -1.37139 |
| C | -2.97183 | 0.450074 | -0.19173 | H | -1.8037 | -0.73158 | -2.53768 |
| C | -4.02349 | 0.445813 | 0.944359 | H | -3.27904 | -1.60196 | -0.76796 |
| O | -1.88877 | 1.294844 | 0.294971 | H | -2.04168 | -1.36929 | 0.448658 |
| C | -3.52481 | 1.148372 | -1.42973 | H | -1.76016 | 1.057026 | 1.227092 |
| O | -3.32153 | 0.016869 | 2.131746 | H | -4.26541 | 0.526404 | -1.93369 |
| C | -5.26208 | -0.40738 | 0.708903 | H | -3.99137 | 2.097743 | -1.15985 |
| C | 3.400689 | 1.750377 | -0.88061 | H | -2.72259 | 1.35563 | -2.13793 |
| C | 2.596007 | 3.025031 | -0.56358 | H | -3.79979 | 0.336079 | 2.905531 |
| C | 1.240249 | 2.724274 | 0.031975 | H | -5.95426 | -0.28484 | 1.544763 |
| O | 0.42376 | 2.120629 | -0.83657 | H | -5.78547 | -0.10442 | -0.19982 |
| O | 0.914547 | 2.982356 | 1.175492 | H | -5.0083 | -1.46518 | 0.631818 |
| H | -4.32484 | 1.489537 | 1.082558 | H | 4.291613 | 2.040188 | -1.44457 |
| H | 4.833337 | -2.37079 | 0.08764 | H | 2.80983 | 1.107466 | -1.53738 |
| H | 5.336332 | -0.22655 | -0.68406 | H | 3.138608 | 3.659231 | 0.137944 |
| H | 3.669977 | -2.49262 | 2.284612 | H | 2.44029 | 3.589631 | -1.48649 |
| H | 2.706486 | -1.07784 | 1.916152 | H | -0.43093 | 1.862576 | -0.39622 |
| H | 2.955211 | 0.776455 | 1.00436 |  |  |  |  |

**Table S6:** (continued)

| **Conformer 2** | | | | | | | |
| --- | --- | --- | --- | --- | --- | --- | --- |
| Atom | X | Y | Z | Atom | X | Y | Z |
| C | 4.401314 | -0.51008 | 0.080202 | H | 3.542199 | 2.313757 | 1.01227 |
| C | 4.149377 | 0.743459 | -0.30178 | H | 4.381491 | -3.23433 | 0.454491 |
| C | 3.721086 | -1.30885 | 1.157636 | H | 3.102899 | -3.31431 | 1.64911 |
| C | 3.098619 | 1.68161 | 0.231355 | H | 2.725582 | -2.41105 | -1.25227 |
| C | 3.43727 | -2.76962 | 0.759418 | H | 2.392101 | -4.01921 | -0.64264 |
| C | 2.4049 | -2.96056 | -0.35968 | H | 0.676808 | -3.05228 | 0.929023 |
| C | 0.9835 | -2.52984 | 0.014165 | H | 0.981468 | -1.46382 | 0.256003 |
| C | -0.04184 | -2.81037 | -1.08948 | H | 0.2867 | -2.33224 | -2.02027 |
| C | -1.47229 | -2.34835 | -0.76908 | H | -0.0578 | -3.88718 | -1.2897 |
| C | -1.59981 | -0.82184 | -0.70516 | H | -2.14424 | -2.74105 | -1.53687 |
| C | -3.00157 | -0.24912 | -0.46003 | H | -1.78901 | -2.79991 | 0.177181 |
| C | -3.61188 | -0.6705 | 0.898248 | H | -0.93742 | -0.42547 | 0.065855 |
| O | -2.81651 | 1.19345 | -0.43177 | H | -1.25054 | -0.4027 | -1.65458 |
| C | -3.96248 | -0.58456 | -1.60467 | H | -3.60306 | 1.571594 | -0.01014 |
| O | -4.80438 | 0.132391 | 1.03295 | H | -4.16105 | -1.65681 | -1.65303 |
| C | -2.70563 | -0.47971 | 2.106721 | H | -4.91459 | -0.07142 | -1.4696 |
| C | 2.529054 | 2.598603 | -0.8605 | H | -3.52861 | -0.27018 | -2.55567 |
| C | 1.510551 | 3.630917 | -0.34108 | H | -5.10182 | 0.092427 | 1.949118 |
| C | 0.207966 | 3.01603 | 0.114192 | H | -3.25993 | -0.70596 | 3.021602 |
| O | -0.50116 | 2.517767 | -0.90343 | H | -1.85023 | -1.15529 | 2.073418 |
| O | -0.16835 | 2.969596 | 1.269703 | H | -2.33997 | 0.546355 | 2.168231 |
| H | -3.90472 | -1.72298 | 0.82414 | H | 3.352152 | 3.148807 | -1.32533 |
| H | 5.22296 | -1.02428 | -0.4166 | H | 2.069953 | 1.999426 | -1.65116 |
| H | 4.786934 | 1.165044 | -1.07717 | H | 1.928616 | 4.188908 | 0.497227 |
| H | 4.38032 | -1.3277 | 2.035239 | H | 1.281215 | 4.338023 | -1.14223 |
| H | 2.803912 | -0.81683 | 1.488311 | H | -1.33182 | 2.077304 | -0.58444 |
| H | 2.293917 | 1.124415 | 0.717907 |  |  |  |  |

**Table S6:** (continued)

| **Conformer 3** | | | | | | | |
| --- | --- | --- | --- | --- | --- | --- | --- |
| Atom | X | Y | Z | Atom | X | Y | Z |
| C | -4.01804 | 1.462231 | 0.231738 | H | -5.08576 | -1.23605 | 1.421321 |
| C | -4.50084 | 0.238369 | 0.004745 | H | -2.379 | 3.732097 | 0.408876 |
| C | -3.07645 | 1.896687 | 1.321771 | H | -1.37864 | 3.150384 | 1.724905 |
| C | -4.22062 | -1.01998 | 0.781564 | H | -0.67418 | 1.206524 | 0.216537 |
| C | -1.94542 | 2.824644 | 0.84569 | H | -1.51594 | 1.993142 | -1.10161 |
| C | -0.98568 | 2.185758 | -0.16206 | H | -0.0746 | 4.074465 | -0.6553 |
| C | 0.249534 | 3.050092 | -0.43809 | H | 0.848368 | 3.117111 | 0.47697 |
| C | 1.123117 | 2.572942 | -1.6089 | H | 0.545559 | 2.675523 | -2.53467 |
| C | 1.636538 | 1.126165 | -1.5313 | H | 1.977591 | 3.252407 | -1.70895 |
| C | 2.584618 | 0.861616 | -0.35649 | H | 0.785614 | 0.444444 | -1.48226 |
| C | 3.036347 | -0.60283 | -0.18702 | H | 2.150738 | 0.904526 | -2.4708 |
| C | 3.974427 | -0.78299 | 1.030871 | H | 3.474921 | 1.486025 | -0.47771 |
| O | 1.880471 | -1.44523 | 0.094186 | H | 2.116084 | 1.172106 | 0.580387 |
| C | 3.677005 | -1.17871 | -1.44567 | H | 1.669845 | -1.31108 | 1.032183 |
| O | 3.182344 | -0.45356 | 2.19275 | H | 4.483934 | -0.53878 | -1.80445 |
| C | 5.265907 | 0.022731 | 1.008292 | H | 4.081076 | -2.17406 | -1.251 |
| C | -3.9833 | -2.25931 | -0.10031 | H | 2.937041 | -1.26442 | -2.24139 |
| C | -2.78365 | -2.16385 | -1.04193 | H | 3.577871 | -0.87378 | 2.96495 |
| C | -1.43342 | -2.12732 | -0.36318 | H | 5.868548 | -0.22891 | 1.88379 |
| O | -0.45245 | -1.82443 | -1.22063 | H | 5.860366 | -0.20622 | 0.121935 |
| O | -1.23174 | -2.35298 | 0.813969 | H | 5.067396 | 1.094826 | 1.030687 |
| H | 4.216027 | -1.85039 | 1.072042 | H | -3.86856 | -3.13374 | 0.544649 |
| H | -4.35775 | 2.262768 | -0.4243 | H | -4.87341 | -2.43771 | -0.71159 |
| H | -5.20293 | 0.12327 | -0.82016 | H | -2.76583 | -3.02416 | -1.71978 |
| H | -3.65532 | 2.434075 | 2.084266 | H | -2.85321 | -1.28391 | -1.68655 |
| H | -2.64376 | 1.02971 | 1.827134 | H | 0.411759 | -1.73562 | -0.73454 |
| H | -3.37337 | -0.88243 | 1.45397 |  |  |  |  |

**Table S6:** (continued)

| **Conformer 4** | | | | | | | |
| --- | --- | --- | --- | --- | --- | --- | --- |
| Atom | X | Y | Z | Atom | X | Y | Z |
| C | -4.49438 | 0.486551 | -0.24681 | H | -4.24318 | -1.88787 | 1.883236 |
| C | -4.55173 | -0.80637 | 0.080002 | H | -2.04511 | 1.279823 | -1.34773 |
| C | -3.51159 | 1.525835 | 0.215962 | H | -3.3401 | 2.38047 | -1.75597 |
| C | -3.65572 | -1.57616 | 1.011053 | H | -1.27705 | 3.626558 | -1.48723 |
| C | -2.66707 | 2.084481 | -0.9438 | H | -2.44633 | 4.122466 | -0.28102 |
| C | -1.79364 | 3.293894 | -0.57957 | H | -0.19294 | 4.006275 | 0.658536 |
| C | -0.74934 | 3.071176 | 0.52503 | H | -1.2509 | 2.884806 | 1.481436 |
| C | 0.241434 | 1.935013 | 0.251283 | H | -0.28782 | 0.978378 | 0.258502 |
| C | 1.374936 | 1.884332 | 1.281448 | H | 0.654925 | 2.043762 | -0.75598 |
| C | 2.270295 | 0.635185 | 1.247235 | H | 1.984722 | 2.789419 | 1.196941 |
| C | 3.129632 | 0.384869 | -0.00184 | H | 0.931524 | 1.922039 | 2.283159 |
| C | 4.209337 | -0.69154 | 0.287283 | H | 2.956766 | 0.696618 | 2.098289 |
| O | 2.268211 | -0.13385 | -1.05224 | H | 1.650165 | -0.24882 | 1.411015 |
| C | 3.808949 | 1.660622 | -0.51046 | H | 2.86376 | -0.52638 | -1.71022 |
| O | 4.821543 | -0.9654 | -0.99104 | H | 4.419784 | 2.1096 | 0.276613 |
| C | 3.70021 | -1.98146 | 0.91503 | H | 4.456151 | 1.438153 | -1.35895 |
| C | -3.06569 | -2.84873 | 0.372256 | H | 3.065396 | 2.39085 | -0.82757 |
| C | -2.09088 | -2.58521 | -0.77536 | H | 5.272658 | -1.81597 | -0.94283 |
| C | -0.76644 | -1.99531 | -0.344 | H | 4.521172 | -2.69925 | 0.995359 |
| O | -0.12328 | -1.40332 | -1.35709 | H | 3.320429 | -1.81113 | 1.922556 |
| O | -0.3109 | -2.05925 | 0.779887 | H | 2.907268 | -2.42786 | 0.313166 |
| H | 4.958268 | -0.23714 | 0.945943 | H | -2.56128 | -3.43377 | 1.144235 |
| H | -5.24285 | 0.85375 | -0.94747 | H | -3.88307 | -3.46823 | -0.00866 |
| H | -5.34628 | -1.39803 | -0.37307 | H | -1.8517 | -3.52312 | -1.28928 |
| H | -4.06808 | 2.355628 | 0.671007 | H | -2.5299 | -1.9319 | -1.53212 |
| H | -2.86157 | 1.127063 | 0.99656 | H | 0.739947 | -1.01633 | -1.05684 |
| H | -2.84928 | -0.94843 | 1.393817 |  |  |  |  |

**Table S6:** (continued)

| **Conformer 5** | | | | | | | |
| --- | --- | --- | --- | --- | --- | --- | --- |
| Atom | X | Y | Z | Atom | X | Y | Z |
| C | 4.620137 | -0.32329 | 0.150464 | H | 2.363976 | 1.372251 | 1.807426 |
| C | 4.141831 | 0.69275 | 0.871931 | H | 3.439255 | -3.10885 | -1.52791 |
| C | 3.941806 | -1.08838 | -0.95263 | H | 4.576386 | -3.00955 | -0.199 |
| C | 2.778032 | 1.320577 | 0.792967 | H | 2.531279 | -3.82856 | 0.729839 |
| C | 3.659618 | -2.56611 | -0.60215 | H | 2.67519 | -2.17256 | 1.283035 |
| C | 2.509582 | -2.78694 | 0.390598 | H | 1.108848 | -1.4573 | -0.56708 |
| C | 1.12372 | -2.48212 | -0.18807 | H | 0.946077 | -3.12892 | -1.05625 |
| C | -0.01493 | -2.66964 | 0.819581 | H | -0.02415 | -3.71233 | 1.155248 |
| C | -1.41036 | -2.31314 | 0.281254 | H | 0.188279 | -2.06443 | 1.711312 |
| C | -1.5605 | -0.81884 | -0.03125 | H | -1.61162 | -2.90799 | -0.61506 |
| C | -2.94073 | -0.34057 | -0.49928 | H | -2.15425 | -2.61593 | 1.024392 |
| C | -4.05025 | -0.50788 | 0.566839 | H | -1.27365 | -0.24015 | 0.850445 |
| O | -2.77911 | 1.084438 | -0.75121 | H | -0.86037 | -0.53885 | -0.8219 |
| C | -3.35684 | -1.01026 | -1.81238 | H | -3.67053 | 1.464222 | -0.78425 |
| O | -5.19065 | 0.194172 | 0.027394 | H | -3.52859 | -2.07919 | -1.67304 |
| C | -3.7076 | 0.021399 | 1.952807 | H | -4.2767 | -0.56761 | -2.19457 |
| C | 2.793062 | 2.741604 | 0.201866 | H | -2.57124 | -0.88055 | -2.5589 |
| C | 1.440856 | 3.4585 | 0.324742 | H | -5.84037 | 0.3141 | 0.72947 |
| C | 0.328733 | 2.759353 | -0.42707 | H | -4.58657 | -0.04388 | 2.599762 |
| O | -0.77754 | 2.601945 | 0.306997 | H | -2.91911 | -0.56959 | 2.420235 |
| O | 0.420756 | 2.380823 | -1.57821 | H | -3.38679 | 1.063559 | 1.91062 |
| H | -4.29502 | -1.57289 | 0.63444 | H | 3.544269 | 3.346174 | 0.717817 |
| H | 5.634748 | -0.65252 | 0.369799 | H | 3.090819 | 2.696257 | -0.84841 |
| H | 4.804755 | 1.133907 | 1.614184 | H | 1.154566 | 3.567075 | 1.371502 |
| H | 3.020266 | -0.59327 | -1.26415 | H | 1.521819 | 4.465142 | -0.09741 |
| H | 4.603537 | -1.07191 | -1.82626 | H | -1.46767 | 2.086488 | -0.18869 |
| H | 2.097999 | 0.694622 | 0.212193 |  |  |  |  |

**Table S6:** (continued)

| **Conformer 6** | | | | | | | |
| --- | --- | --- | --- | --- | --- | --- | --- |
| Atom | X | Y | Z | Atom | X | Y | Z |
| C | 4.229695 | 1.023931 | 0.044872 | H | 2.804124 | -0.79121 | 1.501317 |
| C | 4.398076 | -0.29684 | 0.139678 | H | 1.719497 | 3.341398 | 0.636091 |
| C | 3.291286 | 1.894769 | 0.832852 | H | 2.832367 | 3.343244 | -0.71756 |
| C | 3.680338 | -1.25743 | 1.050344 | H | 1.908165 | 1.251815 | -1.58756 |
| C | 2.288436 | 2.68528 | -0.02936 | H | 0.930114 | 1.021518 | -0.16128 |
| C | 1.334981 | 1.790553 | -0.82706 | H | 0.589964 | 3.327037 | -2.14813 |
| C | 0.180231 | 2.530013 | -1.51676 | H | -0.31477 | 1.835684 | -2.20288 |
| C | -0.87094 | 3.148593 | -0.578 | H | -0.41853 | 3.971938 | -0.01606 |
| C | -1.52839 | 2.188459 | 0.42793 | H | -1.65244 | 3.605636 | -1.19607 |
| C | -2.27193 | 1.01854 | -0.2254 | H | -0.77633 | 1.798251 | 1.117866 |
| C | -2.91878 | 0.01602 | 0.755572 | H | -2.22395 | 2.771516 | 1.037997 |
| C | -3.5998 | -1.15691 | 0.009825 | H | -3.05217 | 1.416437 | -0.88185 |
| O | -1.93644 | -0.53545 | 1.649745 | H | -1.58579 | 0.467588 | -0.87234 |
| C | -3.94463 | 0.690352 | 1.669636 | H | -1.25711 | -1.02805 | 1.157139 |
| O | -2.58718 | -1.83445 | -0.78392 | H | -4.66548 | 1.279195 | 1.101985 |
| C | -4.78248 | -0.79707 | -0.8804 | H | -4.48723 | -0.05544 | 2.254889 |
| C | 3.271617 | -2.56935 | 0.356465 | H | -3.43342 | 1.354603 | 2.365801 |
| C | 2.277248 | -2.42215 | -0.79546 | H | -2.93339 | -2.69781 | -1.04079 |
| C | 0.858675 | -2.10001 | -0.3965 | H | -5.18618 | -1.70255 | -1.33955 |
| O | 0.048445 | -1.99999 | -1.45477 | H | -5.58533 | -0.33752 | -0.30266 |
| O | 0.46106 | -1.95131 | 0.745168 | H | -4.48947 | -0.1146 | -1.67929 |
| H | -3.92704 | -1.85454 | 0.786562 | H | 4.169842 | -3.05041 | -0.04239 |
| H | 4.84985 | 1.556048 | -0.67537 | H | 2.859662 | -3.25295 | 1.10262 |
| H | 5.152628 | -0.75121 | -0.50139 | H | 2.597638 | -1.64991 | -1.50175 |
| H | 2.739707 | 1.303524 | 1.567514 | H | 2.230159 | -3.34668 | -1.37903 |
| H | 3.889986 | 2.613601 | 1.405912 | H | -0.88188 | -1.85311 | -1.14857 |
| H | 4.347845 | -1.52397 | 1.879535 |  |  |  |  |

**Table S6:** (continued)

| **Conformer 7** | | | | | | | |
| --- | --- | --- | --- | --- | --- | --- | --- |
| Atom | X | Y | Z | Atom | X | Y | Z |
| C | -4.45692 | 0.71099 | 0.394252 | H | -3.4263 | -1.12364 | -1.3441 |
| C | -4.6965 | -0.59989 | 0.316918 | H | -2.82189 | 2.786122 | 1.142164 |
| C | -3.55483 | 1.543712 | -0.47201 | H | -1.80553 | 1.405971 | 0.775582 |
| C | -4.13791 | -1.59426 | -0.66377 | H | -1.1225 | 2.504861 | -1.38756 |
| C | -2.40621 | 2.196572 | 0.316217 | H | -2.15089 | 3.867025 | -1.0128 |
| C | -1.52314 | 3.093849 | -0.5555 | H | 0.134616 | 4.464844 | -0.49941 |
| C | -0.37478 | 3.786169 | 0.194341 | H | -0.80033 | 4.420289 | 0.980549 |
| C | 0.670746 | 2.857117 | 0.829919 | H | 1.391979 | 3.475347 | 1.376644 |
| C | 1.430807 | 1.977454 | -0.16865 | H | 0.194507 | 2.219372 | 1.582155 |
| C | 2.50038 | 1.122286 | 0.520808 | H | 0.731706 | 1.323569 | -0.69613 |
| C | 3.194487 | 0.07747 | -0.36165 | H | 1.889594 | 2.618445 | -0.92751 |
| C | 4.321473 | -0.6543 | 0.411544 | H | 3.272364 | 1.77575 | 0.941181 |
| O | 2.19324 | -0.9104 | -0.73061 | H | 2.039963 | 0.604922 | 1.366054 |
| C | 3.767878 | 0.690685 | -1.6439 | H | 2.680933 | -1.68296 | -1.05615 |
| O | 4.741441 | -1.72289 | -0.46266 | H | 4.467956 | 1.49484 | -1.40454 |
| C | 3.934093 | -1.2085 | 1.775672 | H | 4.299163 | -0.06298 | -2.22514 |
| C | -3.47979 | -2.81131 | 0.01473 | H | 2.969011 | 1.100791 | -2.26055 |
| C | -2.21368 | -2.4848 | 0.80556 | H | 5.238361 | -2.36834 | 0.052876 |
| C | -1.00996 | -2.1413 | -0.04409 | H | 4.763453 | -1.79322 | 2.182756 |
| O | -0.05083 | -1.53681 | 0.666075 | H | 3.726847 | -0.40772 | 2.485949 |
| O | -0.8937 | -2.40057 | -1.22529 | H | 3.057407 | -1.85501 | 1.707709 |
| H | 5.151054 | 0.052177 | 0.52993 | H | -4.20044 | -3.27104 | 0.697649 |
| H | -4.96491 | 1.263143 | 1.183524 | H | -3.24583 | -3.56009 | -0.74505 |
| H | -5.38554 | -1.02355 | 1.046503 | H | -2.38106 | -1.6671 | 1.51009 |
| H | -3.13935 | 0.950736 | -1.28997 | H | -1.91562 | -3.34629 | 1.413739 |
| H | -4.15051 | 2.338568 | -0.93907 | H | 0.742459 | -1.34297 | 0.097945 |
| H | -4.95889 | -1.96825 | -1.28748 |  |  |  |  |

**Table S6:** (continued)

| **Conformer 8** | | | | | | | |
| --- | --- | --- | --- | --- | --- | --- | --- |
| Atom | X | Y | Z | Atom | X | Y | Z |
| C | 4.356465 | 0.615472 | -0.32682 | H | 2.964586 | -0.96802 | 1.403255 |
| C | 4.453102 | -0.70135 | -0.12913 | H | 2.840096 | 2.689424 | -1.33594 |
| C | 3.502127 | 1.601802 | 0.41886 | H | 1.758703 | 1.408985 | -0.82197 |
| C | 3.738262 | -1.54155 | 0.894607 | H | 1.171644 | 2.788985 | 1.226634 |
| C | 2.393546 | 2.218596 | -0.45205 | H | 2.196839 | 4.070116 | 0.62802 |
| C | 1.548744 | 3.24722 | 0.306018 | H | -0.12141 | 4.592222 | 0.121922 |
| C | 0.377387 | 3.836928 | -0.49627 | H | 0.777444 | 4.372671 | -1.36471 |
| C | -0.66965 | 2.825496 | -0.98906 | H | -1.41765 | 3.361788 | -1.58387 |
| C | -1.37936 | 2.046332 | 0.124075 | H | -0.19816 | 2.114943 | -1.67618 |
| C | -2.32818 | 0.981628 | -0.43854 | H | -0.63933 | 1.562134 | 0.763683 |
| C | -2.96499 | 0.035518 | 0.587312 | H | -1.92584 | 2.749169 | 0.761469 |
| C | -3.7584 | -1.1167 | -0.09061 | H | -3.13633 | 1.46988 | -0.98987 |
| O | -1.85263 | -0.59328 | 1.302554 | H | -1.78735 | 0.371327 | -1.16611 |
| C | -3.82951 | 0.766766 | 1.613796 | H | -2.20364 | -1.1369 | 2.020456 |
| O | -2.95958 | -1.84352 | -1.0211 | H | -4.59262 | 1.375417 | 1.127718 |
| C | -5.01087 | -0.68021 | -0.83852 | H | -4.33332 | 0.056692 | 2.276044 |
| C | 3.138885 | -2.83149 | 0.304072 | H | -3.21121 | 1.421958 | 2.227397 |
| C | 2.049831 | -2.64789 | -0.77371 | H | -2.14289 | -2.16274 | -0.59816 |
| C | 0.671389 | -2.30789 | -0.26529 | H | -5.50291 | -1.56066 | -1.25492 |
| O | 0.625732 | -1.21312 | 0.489497 | H | -5.71885 | -0.17955 | -0.17826 |
| O | -0.32662 | -2.96016 | -0.53331 | H | -4.76507 | -0.00874 | -1.66317 |
| H | -4.06288 | -1.79061 | 0.724486 | H | 3.946199 | -3.40804 | -0.1571 |
| H | 4.95887 | 1.044089 | -1.12615 | H | 2.748123 | -3.45183 | 1.115568 |
| H | 5.1357 | -1.25313 | -0.77442 | H | 2.348958 | -1.84964 | -1.46136 |
| H | 3.048455 | 1.137239 | 1.297251 | H | 1.947918 | -3.56149 | -1.35775 |
| H | 4.143519 | 2.409503 | 0.793629 | H | -0.31383 | -1.0289 | 0.764705 |
| H | 4.458287 | -1.84773 | 1.663962 |  |  |  |  |

**Table S6:** (continued)

| **Conformer 9** | | | | | | | |
| --- | --- | --- | --- | --- | --- | --- | --- |
| Atom | X | Y | Z | Atom | X | Y | Z |
| C | -4.0802 | -1.78949 | -0.46688 | H | -4.07808 | 1.154873 | -1.45388 |
| C | -4.37662 | -0.52645 | -0.15546 | H | -2.31778 | -3.86437 | 0.011359 |
| C | -2.95587 | -2.28568 | -1.33477 | H | -1.20935 | -3.54504 | -1.31002 |
| C | -3.62434 | 0.716089 | -0.55552 | H | -0.63079 | -1.33197 | -0.25286 |
| C | -1.8598 | -3.05885 | -0.57457 | H | -1.64085 | -1.73524 | 1.118712 |
| C | -1.00882 | -2.17272 | 0.3379 | H | -0.22248 | -3.77816 | 1.537967 |
| C | 0.163602 | -2.91083 | 0.990357 | H | 0.814365 | -3.31799 | 0.208231 |
| C | 0.983756 | -2.05154 | 1.965888 | H | 0.321095 | -1.71592 | 2.77153 |
| C | 1.669924 | -0.81527 | 1.361526 | H | 1.743485 | -2.68285 | 2.441329 |
| C | 2.795164 | -1.16407 | 0.380117 | H | 0.928642 | -0.17985 | 0.876592 |
| C | 3.639764 | -0.00549 | -0.19061 | H | 2.083617 | -0.21997 | 2.180503 |
| C | 2.880081 | 1.009681 | -1.08298 | H | 3.493914 | -1.84264 | 0.885945 |
| O | 4.14586 | 0.835199 | 0.87725 | H | 2.400051 | -1.73428 | -0.46435 |
| C | 4.827203 | -0.57963 | -0.97149 | H | 4.513545 | 0.275363 | 1.571952 |
| O | 1.997957 | 1.830185 | -0.29145 | H | 4.497348 | -1.22698 | -1.78599 |
| C | 2.066082 | 0.411089 | -2.21512 | H | 5.439467 | 0.22178 | -1.38879 |
| C | -3.63655 | 1.77912 | 0.553661 | H | 5.453464 | -1.17753 | -0.30578 |
| C | -2.75418 | 3.007656 | 0.251361 | H | 2.450776 | 2.003559 | 0.547389 |
| C | -1.28725 | 2.711555 | 0.461906 | H | 1.619228 | 1.211711 | -2.80704 |
| O | -0.6086 | 2.509314 | -0.67147 | H | 2.697711 | -0.1798 | -2.87885 |
| O | -0.76983 | 2.642351 | 1.560616 | H | 1.26357 | -0.22509 | -1.83961 |
| H | 3.645372 | 1.670567 | -1.5062 | H | -4.66279 | 2.124973 | 0.704398 |
| H | -4.72613 | -2.56833 | -0.06503 | H | -3.31735 | 1.33666 | 1.501666 |
| H | -5.25573 | -0.3552 | 0.463616 | H | -2.92411 | 3.356799 | -0.76824 |
| H | -3.38441 | -2.95296 | -2.09148 | H | -3.01395 | 3.813544 | 0.939714 |
| H | -2.49423 | -1.45983 | -1.88212 | H | 0.33385 | 2.268933 | -0.46695 |
| H | -2.59622 | 0.467777 | -0.8252 |  |  |  |  |

**Table S6:** (continued)

| **Conformer 10** | | | | | | | |
| --- | --- | --- | --- | --- | --- | --- | --- |
| Atom | X | Y | Z | Atom | X | Y | Z |
| C | 3.403279 | 0.64106 | 1.092828 | H | 5.333298 | -1.82938 | 0.474708 |
| C | 3.680668 | -0.665 | 1.12049 | H | 2.744541 | 2.861228 | -1.4951 |
| C | 3.691912 | 1.627909 | -0.00507 | H | 1.811178 | 1.446108 | -1.04531 |
| C | 4.368662 | -1.49509 | 0.072764 | H | 1.262341 | 2.52941 | 1.161299 |
| C | 2.431105 | 2.251199 | -0.63996 | H | 2.261458 | 3.897424 | 0.732271 |
| C | 1.607051 | 3.125174 | 0.311367 | H | -0.05529 | 4.490892 | 0.384307 |
| C | 0.406067 | 3.819928 | -0.3494 | H | 0.772602 | 4.461677 | -1.1589 |
| C | -0.67843 | 2.890978 | -0.9163 | H | -1.43927 | 3.509345 | -1.4063 |
| C | -1.36069 | 2.000512 | 0.127801 | H | -0.2537 | 2.260354 | -1.70459 |
| C | -2.46505 | 1.13445 | -0.48978 | H | -0.62036 | 1.352861 | 0.603897 |
| C | -3.06665 | 0.06513 | 0.430417 | H | -1.7737 | 2.634152 | 0.918528 |
| C | -4.23204 | -0.6867 | -0.26238 | H | -3.2796 | 1.777332 | -0.84015 |
| O | -2.01759 | -0.90167 | 0.710362 | H | -2.06163 | 0.636443 | -1.37484 |
| C | -3.55414 | 0.6529 | 1.759272 | H | -2.46028 | -1.69285 | 1.054828 |
| O | -4.55526 | -1.77569 | 0.627876 | H | -4.28863 | 1.442456 | 1.582582 |
| C | -3.93898 | -1.21528 | -1.65978 | H | -4.02177 | -0.11818 | 2.371519 |
| C | 3.590396 | -2.75407 | -0.35532 | H | -2.71982 | 1.076117 | 2.317358 |
| C | 2.279218 | -2.46983 | -1.09623 | H | -5.06923 | -2.43113 | 0.142651 |
| C | 1.082845 | -2.20072 | -0.20996 | H | -4.78278 | -1.81681 | -2.00873 |
| O | 0.195012 | -1.3834 | -0.78536 | H | -3.80826 | -0.40129 | -2.37324 |
| O | 0.903933 | -2.70754 | 0.880013 | H | -3.04355 | -1.83931 | -1.66906 |
| H | -5.08593 | -0.00101 | -0.30623 | H | 4.234892 | -3.34056 | -1.01468 |
| H | 2.909265 | 1.053858 | 1.969215 | H | 3.381574 | -3.37975 | 0.516216 |
| H | 3.390868 | -1.21535 | 2.013511 | H | 2.391442 | -1.64687 | -1.80442 |
| H | 4.307347 | 2.438901 | 0.405649 | H | 1.998668 | -3.34751 | -1.69009 |
| H | 4.286704 | 1.161157 | -0.79242 | H | -0.59924 | -1.26051 | -0.19895 |
| H | 4.596751 | -0.89821 | -0.81332 |  |  |  |  |

**Table S6:** (continued)

| **Conformer 11** | | | | | | | |
| --- | --- | --- | --- | --- | --- | --- | --- |
| Atom | X | Y | Z | Atom | X | Y | Z |
| C | -4.58271 | -0.78577 | -0.48202 | H | -2.78364 | 0.913163 | 0.697272 |
| C | -4.2923 | 0.483349 | -0.77521 | H | -3.43282 | -3.49909 | 1.346422 |
| C | -4.17274 | -1.57485 | 0.729714 | H | -4.33263 | -3.53576 | -0.15677 |
| C | -3.45247 | 1.449111 | 0.019385 | H | -1.97814 | -3.99251 | -0.57903 |
| C | -3.58609 | -2.96224 | 0.403814 | H | -2.4337 | -2.49597 | -1.3679 |
| C | -2.27027 | -2.95397 | -0.38578 | H | -1.36162 | -1.16816 | 0.416105 |
| C | -1.11858 | -2.2308 | 0.31666 | H | -1.01506 | -2.61532 | 1.338897 |
| C | 0.226849 | -2.35616 | -0.40414 | H | 0.568224 | -3.39758 | -0.36589 |
| C | 1.29214 | -1.4317 | 0.192265 | H | 0.095455 | -2.11564 | -1.46616 |
| C | 2.669386 | -1.61011 | -0.45298 | H | 0.955005 | -0.40041 | 0.081612 |
| C | 3.824886 | -0.72873 | 0.065431 | H | 1.365783 | -1.61556 | 1.269022 |
| C | 3.674982 | 0.794423 | -0.18299 | H | 2.981872 | -2.65349 | -0.32209 |
| O | 3.916381 | -0.80154 | 1.510859 | H | 2.591104 | -1.46506 | -1.53462 |
| C | 5.147254 | -1.2144 | -0.53849 | H | 3.872261 | -1.72624 | 1.78342 |
| O | 2.646346 | 1.361162 | 0.656718 | H | 5.122132 | -1.19559 | -1.62951 |
| C | 3.382566 | 1.202041 | -1.61533 | H | 5.979214 | -0.59563 | -0.19747 |
| C | -2.64922 | 2.397348 | -0.88257 | H | 5.337852 | -2.24432 | -0.22901 |
| C | -1.7991 | 3.42344 | -0.10898 | H | 2.7038 | 0.909113 | 1.511978 |
| C | -0.5879 | 2.823742 | 0.566788 | H | 3.345021 | 2.290455 | -1.68463 |
| O | 0.33339 | 2.416129 | -0.31217 | H | 4.167839 | 0.853108 | -2.28627 |
| O | -0.44488 | 2.718878 | 1.769994 | H | 2.42753 | 0.804286 | -1.96126 |
| H | 4.624874 | 1.244411 | 0.127258 | H | -2.00525 | 1.822597 | -1.55331 |
| H | -5.21836 | -1.32497 | -1.18309 | H | -3.34455 | 2.951399 | -1.51949 |
| H | -4.7193 | 0.894157 | -1.68868 | H | -1.44051 | 4.185211 | -0.80579 |
| H | -3.47678 | -1.00544 | 1.348602 | H | -2.40007 | 3.917053 | 0.65528 |
| H | -5.06382 | -1.73174 | 1.350982 | H | 1.123693 | 2.030199 | 0.148848 |
| H | -4.10547 | 2.057875 | 0.658812 |  |  |  |  |

**Table S6:** (continued)

| **Conformer 12** | | | | | | | |
| --- | --- | --- | --- | --- | --- | --- | --- |
| Atom | X | Y | Z | Atom | X | Y | Z |
| C | -4.06591 | 1.503945 | 0.43527 | H | -5.00797 | -1.34498 | 1.499598 |
| C | -4.61787 | 0.306701 | 0.225721 | H | -2.33204 | 3.735395 | 0.411475 |
| C | -2.88803 | 1.842019 | 1.307263 | H | -1.07274 | 2.982623 | 1.367719 |
| C | -4.21611 | -1.01877 | 0.814031 | H | -0.97083 | 1.154116 | -0.48175 |
| C | -1.85989 | 2.771882 | 0.63733 | H | -2.00977 | 2.18538 | -1.4279 |
| C | -1.24497 | 2.200769 | -0.6454 | H | 0.161181 | 2.710192 | -2.20331 |
| C | -0.02312 | 2.980942 | -1.15756 | H | -0.26146 | 4.050756 | -1.16009 |
| C | 1.273815 | 2.755718 | -0.3633 | H | 2.018943 | 3.490618 | -0.68819 |
| C | 1.860274 | 1.348191 | -0.52692 | H | 1.099838 | 2.955841 | 0.700156 |
| C | 3.128244 | 1.145937 | 0.307219 | H | 1.114882 | 0.604457 | -0.24626 |
| C | 3.889912 | -0.18559 | 0.157729 | H | 2.080387 | 1.175513 | -1.58543 |
| C | 3.078512 | -1.46953 | 0.479123 | H | 3.841587 | 1.943844 | 0.064504 |
| O | 4.281216 | -0.39465 | -1.22369 | H | 2.89176 | 1.282806 | 1.366861 |
| C | 5.152735 | -0.15038 | 1.025397 | H | 4.650397 | 0.425235 | -1.5739 |
| O | 2.146923 | -1.76866 | -0.58067 | H | 4.911272 | -0.02972 | 2.082998 |
| C | 2.314574 | -1.44924 | 1.789949 | H | 5.728521 | -1.06921 | 0.901974 |
| C | -4.02743 | -2.12976 | -0.23607 | H | 5.783333 | 0.69134 | 0.730652 |
| C | -2.83652 | -1.92168 | -1.17071 | H | 2.597981 | -1.53017 | -1.40563 |
| C | -1.48383 | -2.11533 | -0.5217 | H | 1.830397 | -2.41435 | 1.945651 |
| O | -0.49483 | -1.61477 | -1.27215 | H | 2.988937 | -1.27528 | 2.629083 |
| O | -1.28938 | -2.67427 | 0.538476 | H | 1.545293 | -0.67604 | 1.795471 |
| H | 3.806277 | -2.2888 | 0.5063 | H | -3.91844 | -3.08873 | 0.275132 |
| H | -4.52164 | 2.35667 | -0.06658 | H | -4.93255 | -2.20073 | -0.8467 |
| H | -5.48316 | 0.265288 | -0.43469 | H | -2.87769 | -2.63519 | -2.00131 |
| H | -3.25508 | 2.340103 | 2.213982 | H | -2.85589 | -0.93053 | -1.62958 |
| H | -2.38397 | 0.932687 | 1.643539 | H | 0.386683 | -1.74916 | -0.84198 |
| H | -3.31224 | -0.92615 | 1.418216 |  |  |  |  |

**Table S6:** (continued)

| **Conformer 13** | | | | | | | |
| --- | --- | --- | --- | --- | --- | --- | --- |
| Atom | X | Y | Z | Atom | X | Y | Z |
| C | -4.09389 | 1.012034 | 0.715772 | H | -4.16375 | -2.09053 | 1.774621 |
| C | -4.51534 | -0.25266 | 0.784255 | H | -2.9345 | 2.27889 | -1.24629 |
| C | -2.67628 | 1.512384 | 0.756345 | H | -2.84006 | 3.527578 | -0.02211 |
| C | -3.68993 | -1.49603 | 0.986209 | H | -0.55891 | 1.812722 | -1.10765 |
| C | -2.40538 | 2.570679 | -0.3331 | H | -0.83852 | 3.502476 | -1.47536 |
| C | -0.92274 | 2.751697 | -0.68053 | H | -0.41451 | 4.090417 | 0.930218 |
| C | -0.02381 | 3.166601 | 0.487635 | H | -0.07216 | 2.413299 | 1.279722 |
| C | 1.441011 | 3.406047 | 0.088166 | H | 1.473532 | 4.223426 | -0.64109 |
| C | 2.193743 | 2.2039 | -0.50902 | H | 1.991634 | 3.762156 | 0.966585 |
| C | 2.360276 | 1.036471 | 0.470755 | H | 1.687474 | 1.856971 | -1.41383 |
| C | 3.091198 | -0.20108 | -0.06776 | H | 3.177258 | 2.557417 | -0.8279 |
| C | 3.358677 | -1.22502 | 1.066205 | H | 2.909521 | 1.388732 | 1.35125 |
| O | 2.22458 | -0.83952 | -1.0471 | H | 1.379679 | 0.716445 | 0.82709 |
| C | 4.415355 | 0.15314 | -0.75398 | H | 2.585336 | -1.73178 | -1.17171 |
| O | 3.860749 | -2.40252 | 0.398229 | H | 5.060654 | 0.717407 | -0.07623 |
| C | 2.158132 | -1.57913 | 1.932642 | H | 4.944144 | -0.75148 | -1.05444 |
| C | -3.57321 | -2.38488 | -0.2701 | H | 4.235977 | 0.756941 | -1.64263 |
| C | -2.63799 | -1.81951 | -1.34103 | H | 3.755306 | -3.1598 | 0.985254 |
| C | -1.17496 | -1.86444 | -0.95101 | H | 2.431674 | -2.37918 | 2.626494 |
| O | -0.45811 | -0.89492 | -1.5288 | H | 1.841551 | -0.72789 | 2.536307 |
| O | -0.68542 | -2.69618 | -0.21261 | H | 1.316123 | -1.91771 | 1.326672 |
| H | 4.154706 | -0.8134 | 1.697327 | H | -3.21629 | -3.37424 | 0.02344 |
| H | -4.85094 | 1.784964 | 0.591991 | H | -4.56503 | -2.52345 | -0.70961 |
| H | -5.58592 | -0.42781 | 0.694316 | H | -2.72346 | -2.4082 | -2.26149 |
| H | -2.46757 | 1.948207 | 1.740495 | H | -2.90397 | -0.79536 | -1.60753 |
| H | -1.9768 | 0.682301 | 0.63747 | H | 0.495321 | -0.94521 | -1.25146 |
| H | -2.69103 | -1.25056 | 1.353501 |  |  |  |  |

**Table S7.** Specific rotation calculation of **3a** (14*R*, 15*R*), (A) Boltzmann distribution of energy minimized conformers, and calculated optical rotation value, (B) Optimized Z-matrixes of **3a** conformers in the MeOH (Ǻ)

(A)

| Conformer | Calculated Energy (G) (atomic units) | Relative Energy (kcal/mol) | Boltzmann Weights (%) | **ORD Value** | |
| --- | --- | --- | --- | --- | --- |
| 1 | -968.337346 | 0.000000 | 0.000012896 | 53.04 | **-18.74** |
| 2 | -968.338559 | -0.761169 | 0.002949950 | -104.29 |  |
| 3 | -968.337782 | -0.273594 | 0.000090888 | 56.32 |  |
| 4 | -968.339073 | -1.083709 | 0.029483100 | 93.89 |  |
| 5 | -968.336445 | 0.565386 | 0.000000228 | 17.56 |  |
| 6 | -968.336823 | 0.328187 | 0.000001239 | -56.68 |  |
| 7 | -968.335958 | 0.870983 | 0.000000026 | -141.59 |  |
| 8 | -968.335281 | 1.295807 | 0.000000001 | -38.46 |  |
| 9 | -968.340888 | -2.222639 | 99.965131187 | -18.77 |  |
| 10 | -968.336389 | 0.600527 | 0.000000177 | -33.33 |  |
| 11 | -968.338506 | -0.727911 | 0.002326627 | -21.86 |  |
| 12 | -968.332235 | 3.207201 | 0.000000000 | -127.12 |  |
| 13 | -968.33565 | 1.064256 | 0.000000006 | 61.9 |  |
| 14 | -968.334698 | 1.661645 | 0.000000000 | 11.88 |  |
| 15 | -968.337062 | 0.178213 | 0.000003615 | -53.12 |  |
| 16 | -968.336142 | 0.755521 | 0.000000059 | -111.1 |  |

(B)

| **Conformer 1** | | | | | | | |
| --- | --- | --- | --- | --- | --- | --- | --- |
| Atom | X | Y | Z | Atom | X | Y | Z |
| C | -4.2156 | -0.33687 | 1.09259 | H | -1.97011 | 1.193873 | 0.980903 |
| C | -4.09489 | 0.94874 | 0.755076 | H | -2.40504 | -3.28961 | 1.142919 |
| C | -3.14082 | -1.29929 | 1.515464 | H | -4.10048 | -3.08178 | 0.77121 |
| C | -2.84434 | 1.780902 | 0.691263 | H | -2.92446 | -3.38085 | -1.31034 |
| C | -3.12048 | -2.59948 | 0.685619 | H | -3.53997 | -1.74387 | -1.24413 |
| C | -2.79647 | -2.41672 | -0.80478 | H | -1.36075 | -1.59344 | -2.17849 |
| C | -1.39528 | -1.87281 | -1.11963 | H | -1.22942 | -0.94453 | -0.56599 |
| C | -0.24941 | -2.85577 | -0.84973 | H | -0.31074 | -3.24292 | 0.173342 |
| C | 1.146994 | -2.25189 | -1.07377 | H | -0.37516 | -3.72167 | -1.50872 |
| C | 1.610409 | -1.37352 | 0.097099 | H | 1.865693 | -3.06235 | -1.21955 |
| C | 2.874238 | -0.54059 | -0.15159 | H | 1.143243 | -1.67019 | -2.00066 |
| C | 3.220168 | 0.310892 | 1.094759 | H | 0.810644 | -0.68474 | 0.384395 |
| O | 2.546306 | 0.412302 | -1.20687 | H | 1.786582 | -2.01743 | 0.962391 |
| C | 4.060859 | -1.38527 | -0.62098 | H | 3.238293 | 1.092011 | -1.15817 |
| O | 4.232123 | 1.248313 | 0.658638 | H | 4.269096 | -2.19682 | 0.078604 |
| C | 3.706737 | -0.45928 | 2.314696 | H | 3.845275 | -1.82249 | -1.5954 |
| C | -2.62587 | 2.398955 | -0.69992 | H | 4.958686 | -0.77203 | -0.71189 |
| C | -1.41869 | 3.350008 | -0.76986 | H | 4.165131 | 2.045534 | 1.19622 |
| C | -0.08777 | 2.675573 | -0.52822 | H | 3.906181 | 0.239061 | 3.130316 |
| O | 0.180794 | 1.725839 | -1.42838 | H | 2.956552 | -1.16999 | 2.665398 |
| O | 0.670725 | 2.960989 | 0.379398 | H | 4.628854 | -1.0009 | 2.098951 |
| H | 2.318139 | 0.875289 | 1.348324 | H | -2.50967 | 1.605412 | -1.44172 |
| H | -5.21837 | -0.76181 | 1.071424 | H | -3.51611 | 2.966897 | -0.98491 |
| H | -5.00562 | 1.480793 | 0.484503 | H | -1.37961 | 3.803928 | -1.76438 |
| H | -2.15878 | -0.82374 | 1.495494 | H | -1.51896 | 4.152083 | -0.03817 |
| H | -3.31925 | -1.57788 | 2.561514 | H | 1.054846 | 1.286706 | -1.23591 |
| H | -2.92307 | 2.596405 | 1.421684 |  |  |  |  |

**Table S7:** (continued)

| **Conformer 2** | | | | | | | |
| --- | --- | --- | --- | --- | --- | --- | --- |
| Atom | X | Y | Z | Atom | X | Y | Z |
| C | -4.64898 | 0.505518 | -0.04704 | H | -3.24696 | -2.03264 | 1.536314 |
| C | -4.50474 | -0.73821 | 0.415464 | H | -4.18329 | 3.079604 | 0.712205 |
| C | -3.56371 | 1.425312 | -0.53315 | H | -2.86834 | 2.141332 | 1.378575 |
| C | -3.2199 | -1.5069 | 0.575093 | H | -2.74631 | 4.146485 | -0.9315 |
| C | -3.2504 | 2.570655 | 0.447209 | H | -2.08323 | 4.365632 | 0.674893 |
| C | -2.2649 | 3.615023 | -0.10278 | H | -1.09431 | 2.368798 | -1.42647 |
| C | -0.91908 | 3.065343 | -0.59957 | H | -0.34214 | 3.892571 | -1.02746 |
| C | -0.06949 | 2.367822 | 0.467343 | H | 0.250327 | 3.10146 | 1.217216 |
| C | 1.153088 | 1.655637 | -0.11995 | H | -0.67321 | 1.628973 | 1.00427 |
| C | 2.018912 | 0.986076 | 0.953059 | H | 0.804707 | 0.906007 | -0.83682 |
| C | 3.021884 | -0.07996 | 0.472302 | H | 1.748153 | 2.374022 | -0.69292 |
| C | 3.906661 | 0.402971 | -0.70062 | H | 2.563389 | 1.751159 | 1.514409 |
| O | 2.292828 | -1.20497 | -0.10915 | H | 1.365699 | 0.491262 | 1.680414 |
| C | 3.843029 | -0.60048 | 1.651995 | H | 1.955748 | -1.78549 | 0.595141 |
| O | 4.717286 | -0.67392 | -1.19195 | H | 4.398243 | 0.207555 | 2.129868 |
| C | 4.838541 | 1.555851 | -0.37059 | H | 3.180571 | -1.03377 | 2.406045 |
| C | -2.9904 | -2.53899 | -0.54304 | H | 4.550636 | -1.3641 | 1.329063 |
| C | -1.79777 | -3.47213 | -0.27845 | H | 4.129967 | -1.43727 | -1.28261 |
| C | -0.46916 | -2.77885 | -0.10649 | H | 5.393852 | 1.840442 | -1.26589 |
| O | -0.1754 | -1.92797 | -1.09609 | H | 4.283416 | 2.429665 | -0.02768 |
| O | 0.286734 | -2.9771 | 0.830187 | H | 5.558346 | 1.275575 | 0.400289 |
| H | 3.227898 | 0.712697 | -1.50296 | H | -3.88091 | -3.16418 | -0.65212 |
| H | -5.65637 | 0.916252 | -0.07853 | H | -2.85336 | -2.02047 | -1.49457 |
| H | -5.40423 | -1.27517 | 0.709404 | H | -1.96499 | -4.06956 | 0.617954 |
| H | -2.65446 | 0.858907 | -0.74036 | H | -1.68838 | -4.1642 | -1.11982 |
| H | -3.87885 | 1.861065 | -1.48934 | H | 0.702634 | -1.51388 | -0.90552 |
| H | -2.36735 | -0.82514 | 0.619651 |  |  |  |  |

**Table S7:** (continued)

| **Conformer 3** | | | | | | | |
| --- | --- | --- | --- | --- | --- | --- | --- |
| Atom | X | Y | Z | Atom | X | Y | Z |
| C | -4.926272 | -0.233675 | -0.441434 | H | -3.771033 | 1.768128 | 1.036424 |
| C | -4.559973 | 0.995539 | -0.810443 | H | -3.954502 | -2.910398 | 1.533421 |
| C | -4.572767 | -0.966542 | 0.824618 | H | -4.881611 | -2.950502 | 0.047983 |
| C | -3.695042 | 1.957064 | -0.037384 | H | -2.672083 | -3.568978 | -0.567534 |
| C | -4.084900 | -2.408162 | 0.568705 | H | -2.895261 | -1.941201 | -1.169353 |
| C | -2.791643 | -2.527530 | -0.249386 | H | -1.656562 | -1.09533 | 0.916986 |
| C | -1.517562 | -2.091028 | 0.482779 | H | -1.33471 | -2.76616 | 1.327337 |
| C | -0.291281 | -2.063386 | -0.433794 | H | -0.13656 | -3.059149 | -0.866158 |
| C | 0.998142 | -1.606061 | 0.257070 | H | -0.498051 | -1.395492 | -1.278369 |
| C | 2.155980 | -1.466985 | -0.738345 | H | 0.813131 | -0.6485 | 0.753801 |
| C | 3.520336 | -1.013321 | -0.181439 | H | 1.257714 | -2.318951 | 1.045597 |
| C | 3.482179 | 0.440718 | 0.346923 | H | 2.317184 | -2.439194 | -1.220802 |
| O | 4.456954 | -0.947702 | -1.289333 | H | 1.862928 | -0.773839 | -1.530994 |
| C | 4.046120 | -1.987852 | 0.874136 | H | 4.324904 | -1.718573 | -1.854599 |
| O | 3.124013 | 1.332489 | -0.733633 | H | 3.476643 | -1.918972 | 1.803344 |
| C | 4.777706 | 0.926749 | 0.987778 | H | 3.967631 | -3.013968 | 0.50707 |
| C | -2.213780 | 1.886614 | -0.446957 | H | 5.093106 | -1.78566 | 1.097209 |
| C | -1.352928 | 2.915886 | 0.306422 | H | 3.598704 | 1.023161 | -1.520573 |
| C | 0.118905 | 2.580859 | 0.316784 | H | 4.673664 | 1.982461 | 1.242494 |
| O | 0.609435 | 2.294819 | -0.892306 | H | 4.996908 | 0.379839 | 1.905737 |
| O | 0.800214 | 2.555093 | 1.325077 | H | 5.622755 | 0.815427 | 0.306022 |
| H | 2.671408 | 0.530507 | 1.069553 | H | -1.845575 | 0.879537 | -0.23876 |
| H | -5.555928 | -0.794917 | -1.130615 | H | -2.113665 | 2.035394 | -1.524473 |
| H | -4.900002 | 1.350482 | -1.780875 | H | -1.475474 | 3.907037 | -0.142313 |
| H | -3.830610 | -0.412146 | 1.402237 | H | -1.664489 | 2.991241 | 1.348671 |
| H | -5.466875 | -1.028860 | 1.457810 | H | 1.555164 | 1.990665 | -0.811961 |
| H | -4.063103 | 2.975992 | -0.198951 |  |  |  |  |

**Table S7:** (continued)

| **Conformer 4** | | | | | | | |
| --- | --- | --- | --- | --- | --- | --- | --- |
| Atom | X | Y | Z | Atom | X | Y | Z |
| C | -4.607411 | 1.02023 | -0.36482 | H | -5.24834 | -1.72218 | 1.253329 |
| C | -4.983037 | -0.256826 | -0.259434 | H | -2.0476 | 1.29846 | -1.15628 |
| C | -3.50387 | 1.715868 | 0.380331 | H | -2.70691 | 2.920978 | -1.23778 |
| C | -4.426648 | -1.317831 | 0.651047 | H | -1.40713 | 3.533274 | 0.832219 |
| C | -2.350836 | 2.152544 | -0.542034 | H | -0.82484 | 1.884759 | 0.935286 |
| C | -1.130217 | 2.661652 | 0.226961 | H | 0.207502 | 2.220486 | -1.40461 |
| C | 0.049604 | 3.026886 | -0.680361 | H | -0.21635 | 3.90951 | -1.27214 |
| C | 1.35806 | 3.31171 | 0.073588 | H | 2.07927 | 3.761474 | -0.61828 |
| C | 2.012878 | 2.092081 | 0.745557 | H | 1.162427 | 4.06962 | 0.840344 |
| C | 2.626921 | 1.103013 | -0.254531 | H | 2.79411 | 2.453191 | 1.419453 |
| C | 3.136423 | -0.214278 | 0.345855 | H | 1.283638 | 1.575795 | 1.374413 |
| C | 3.701771 | -1.137193 | -0.761712 | H | 1.896033 | 0.851332 | -1.02833 |
| O | 1.970721 | -0.904455 | 0.887697 | H | 3.459579 | 1.594575 | -0.76423 |
| C | 4.139344 | -0.001652 | 1.482402 | H | 2.26405 | -1.81523 | 1.050459 |
| O | 3.891859 | -2.425083 | -0.134894 | H | 4.972824 | 0.625322 | 1.160715 |
| C | 4.992897 | -0.673617 | -1.422885 | H | 3.650832 | 0.487192 | 2.324524 |
| C | -3.779201 | -2.497101 | -0.103542 | H | 4.541009 | -0.9559 | 1.826333 |
| C | -2.489519 | -2.141242 | -0.842658 | H | 3.865359 | -3.10697 | -0.81548 |
| C | -1.295615 | -1.892848 | 0.053688 | H | 5.28956 | -1.39476 | -2.18753 |
| O | -0.318126 | -1.235285 | -0.580073 | H | 4.867271 | 0.29314 | -1.91312 |
| O | -1.201183 | -2.264095 | 1.206897 | H | 5.803524 | -0.59655 | -0.69692 |
| H | 2.918497 | -1.234533 | -1.520737 | H | -3.57511 | -3.30286 | 0.604968 |
| H | -5.133607 | 1.637248 | -1.091407 | H | -4.49401 | -2.89178 | -0.83157 |
| H | -5.789999 | -0.595001 | -0.907847 | H | -2.19798 | -2.96197 | -1.50781 |
| H | -3.907902 | 2.602822 | 0.883594 | H | -2.62458 | -1.27011 | -1.48715 |
| H | -3.103853 | 1.071559 | 1.166262 | H | 0.474458 | -1.12106 | 0.011224 |
| H | -3.707159 | -0.898869 | 1.35636 |  |  |  |  |

**Table S7:** (continued)

| **Conformer 5** | | | | | | | |
| --- | --- | --- | --- | --- | --- | --- | --- |
| Atom | X | Y | Z | Atom | X | Y | Z |
| C | 4.374962 | -0.57516 | 0.272127 | H | 2.15389 | 0.762722 | -0.63629 |
| C | 4.283368 | 0.534095 | -0.46477 | H | 4.055241 | -3.24602 | 0.994127 |
| C | 3.291179 | -1.2283 | 1.082397 | H | 2.410037 | -3.1249 | 1.566708 |
| C | 3.055603 | 1.377403 | -0.69337 | H | 3.392587 | -2.90841 | -1.31959 |
| C | 3.103079 | -2.7343 | 0.815677 | H | 2.448356 | -4.1985 | -0.6084 |
| C | 2.603998 | -3.11389 | -0.58803 | H | 1.532102 | -1.34816 | -1.22018 |
| C | 1.321321 | -2.40989 | -1.05713 | H | 1.046428 | -2.80962 | -2.03973 |
| C | 0.122292 | -2.52851 | -0.11205 | H | -0.1501 | -3.58279 | 0.016496 |
| C | -1.09577 | -1.73256 | -0.59617 | H | 0.40027 | -2.16354 | 0.881588 |
| C | -2.18533 | -1.63415 | 0.475864 | H | -0.76166 | -0.72773 | -0.87246 |
| C | -3.45156 | -0.82961 | 0.120272 | H | -1.49213 | -2.18898 | -1.50845 |
| C | -3.13244 | 0.652577 | -0.18376 | H | -2.51244 | -2.64608 | 0.745111 |
| O | -4.28954 | -0.76835 | 1.303493 | H | -1.75093 | -1.19728 | 1.378403 |
| C | -4.2281 | -1.48544 | -1.02335 | H | -4.29988 | -1.63878 | 1.720197 |
| O | -2.45566 | 1.240195 | 0.948453 | H | -3.69726 | -1.3925 | -1.97321 |
| C | -4.34052 | 1.506976 | -0.55312 | H | -4.36556 | -2.54949 | -0.81715 |
| C | 2.92486 | 2.548436 | 0.296915 | H | -5.21226 | -1.03137 | -1.13356 |
| C | 1.782904 | 3.517481 | -0.05394 | H | -2.93072 | 0.961684 | 1.745247 |
| C | 0.412061 | 2.883341 | -0.12175 | H | -4.02088 | 2.543429 | -0.67037 |
| O | 0.052395 | 2.280151 | 1.015854 | H | -4.78283 | 1.183539 | -1.49628 |
| O | -0.30807 | 2.921034 | -1.10145 | H | -5.10674 | 1.464305 | 0.222783 |
| H | -2.40806 | 0.691702 | -0.99693 | H | 2.782561 | 2.156352 | 1.306545 |
| H | 5.348489 | -1.05974 | 0.331327 | H | 3.85568 | 3.122263 | 0.311234 |
| H | 5.189994 | 0.892966 | -0.94693 | H | 1.739461 | 4.308539 | 0.700932 |
| H | 3.552107 | -1.11647 | 2.142482 | H | 1.966369 | 3.989047 | -1.01963 |
| H | 2.343809 | -0.70141 | 0.953784 | H | -0.86186 | 1.899679 | 0.927651 |
| H | 3.091506 | 1.78033 | -1.71114 |  |  |  |  |

**Table S7:** (continued)

| **Conformer 6** | | | | | | | |
| --- | --- | --- | --- | --- | --- | --- | --- |
| Atom | X | Y | Z | Atom | X | Y | Z |
| C | -4.100809 | 1.65156 | -0.407077 | H | -2.943771 | -0.557322 | -1.470818 |
| C | -4.615593 | 0.427128 | -0.542821 | H | -2.134823 | 3.212364 | 0.8852 |
| C | -2.761097 | 2.133177 | -0.890073 | H | -1.69752 | 1.513436 | 0.882192 |
| C | -3.98376 | -0.762805 | -1.213942 | H | -0.041068 | 1.943288 | -0.942973 |
| C | -1.760077 | 2.403307 | 0.247674 | H | -0.405427 | 3.655396 | -0.882669 |
| C | -0.361257 | 2.746146 | -0.270717 | H | 0.395716 | 3.802864 | 1.445826 |
| C | 0.675843 | 2.933368 | 0.841089 | H | 0.634865 | 2.073927 | 1.518753 |
| C | 2.113565 | 3.131735 | 0.335051 | H | 2.123929 | 3.970932 | -0.369345 |
| C | 2.752406 | 1.911291 | -0.351211 | H | 2.749014 | 3.434052 | 1.175052 |
| C | 3.066224 | 0.763406 | 0.617917 | H | 2.097264 | 1.561262 | -1.153871 |
| C | 3.570513 | -0.550354 | -0.013599 | H | 3.675932 | 2.236247 | -0.837602 |
| C | 2.428651 | -1.304549 | -0.742415 | H | 3.840706 | 1.107352 | 1.315197 |
| O | 3.942828 | -1.458945 | 1.056216 | H | 2.187529 | 0.526831 | 1.219946 |
| C | 4.779125 | -0.317608 | -0.920595 | H | 4.406228 | -0.960789 | 1.740217 |
| O | 1.422395 | -1.659919 | 0.228272 | H | 4.49736 | 0.194003 | -1.843211 |
| C | 2.863249 | -2.546944 | -1.512425 | H | 5.518741 | 0.300358 | -0.405684 |
| C | -4.059919 | -2.058381 | -0.385099 | H | 5.250183 | -1.264608 | -1.181798 |
| C | -3.407971 | -1.989797 | 1.000086 | H | 1.895792 | -1.990421 | 1.007605 |
| C | -1.921695 | -1.712773 | 1.064728 | H | 1.978859 | -3.050376 | -1.906104 |
| O | -1.233041 | -2.153401 | 0.008591 | H | 3.505543 | -2.288349 | -2.355336 |
| O | -1.391729 | -1.169075 | 2.015314 | H | 3.400343 | -3.244069 | -0.866512 |
| H | 1.937722 | -0.611682 | -1.426666 | H | -5.11076 | -2.32627 | -0.23696 |
| H | -4.700584 | 2.392935 | 0.117824 | H | -3.611921 | -2.87053 | -0.961631 |
| H | -5.608353 | 0.250282 | -0.131234 | H | -3.88979 | -1.23899 | 1.627594 |
| H | -2.320774 | 1.411014 | -1.581219 | H | -3.550536 | -2.950018 | 1.509483 |
| H | -2.900941 | 3.058251 | -1.462518 | H | -0.26567 | -1.954701 | 0.126844 |
| H | -4.501798 | -0.953385 | -2.162152 |  |  |  |  |

**Table S7:** (continued)

| **Conformer 7** | | | | | | | |
| --- | --- | --- | --- | --- | --- | --- | --- |
| Atom | X | Y | Z | Atom | X | Y | Z |
| C | 4.537076 | 0.492696 | -0.15456 | H | 2.826728 | -0.91242 | 1.447612 |
| C | 4.582901 | -0.79241 | 0.203544 | H | 3.443429 | 2.370312 | -1.73688 |
| C | 3.539897 | 1.541807 | 0.251542 | H | 2.139887 | 1.266301 | -1.3665 |
| C | 3.657045 | -1.54205 | 1.122257 | H | 2.478504 | 4.128219 | -0.33043 |
| C | 2.74005 | 2.080258 | -0.94864 | H | 1.354792 | 3.599007 | -1.56551 |
| C | 1.844148 | 3.287873 | -0.63542 | H | 1.240715 | 2.91527 | 1.41477 |
| C | 0.767135 | 3.072642 | 0.439032 | H | 0.191462 | 4.000724 | 0.534432 |
| C | -0.19371 | 1.913054 | 0.158895 | H | -0.5959 | 2.004962 | -0.85429 |
| C | -1.33863 | 1.841399 | 1.174946 | H | 0.358409 | 0.969793 | 0.179721 |
| C | -2.18667 | 0.55874 | 1.164834 | H | -0.90992 | 1.921213 | 2.180618 |
| C | -3.04073 | 0.254977 | -0.07588 | H | -1.98303 | 2.719233 | 1.061662 |
| C | -3.95208 | -0.97052 | 0.199573 | H | -1.53739 | -0.30491 | 1.335895 |
| O | -2.1402 | -0.15827 | -1.1444 | H | -2.86395 | 0.612317 | 2.020561 |
| C | -3.8411 | 1.463876 | -0.56517 | H | -2.70082 | -0.64411 | -1.77171 |
| O | -4.45979 | -1.37641 | -1.09253 | H | -4.44736 | 1.889237 | 0.236733 |
| C | -5.10741 | -0.74765 | 1.166198 | H | -3.16409 | 2.237243 | -0.92676 |
| C | 3.112889 | -2.84533 | 0.50538 | H | -4.50481 | 1.181117 | -1.38388 |
| C | 2.180898 | -2.63559 | -0.68888 | H | -4.68267 | -2.31337 | -1.05695 |
| C | 0.831261 | -2.05555 | -0.32624 | H | -5.66151 | -1.68052 | 1.291182 |
| O | 0.263577 | -1.41059 | -1.35044 | H | -4.7564 | -0.43989 | 2.152269 |
| O | 0.297022 | -2.17245 | 0.758823 | H | -5.79736 | 0.007721 | 0.787732 |
| H | -3.30224 | -1.76529 | 0.580209 | H | 3.95424 | -3.46383 | 0.179186 |
| H | 5.30935 | 0.843996 | -0.83731 | H | 2.585656 | -3.4122 | 1.275546 |
| H | 5.392913 | -1.39347 | -0.20785 | H | 2.640499 | -2.00203 | -1.44944 |
| H | 2.860611 | 1.157962 | 1.014498 | H | 1.975129 | -3.59582 | -1.17557 |
| H | 4.080483 | 2.378923 | 0.712376 | H | -0.61786 | -1.03005 | -1.09196 |
| H | 4.209711 | -1.81337 | 2.03 |  |  |  |  |

**Table S7:** (continued)

| **Conformer 8** | | | | | | | |
| --- | --- | --- | --- | --- | --- | --- | --- |
| Atom | X | Y | Z | Atom | X | Y | Z |
| C | -3.8294 | 1.495337 | 0.1906 | H | -3.19475 | -0.58797 | -1.45593 |
| C | -4.60432 | 0.49829 | -0.24347 | H | -2.41362 | 3.206862 | -1.44232 |
| C | -2.3421 | 1.636325 | 0.032102 | H | -2.2302 | 3.792098 | 0.200693 |
| C | -4.18259 | -0.72528 | -1.01191 | H | -0.07467 | 2.324229 | -1.40126 |
| C | -1.90078 | 3.011081 | -0.49481 | H | -0.17387 | 4.064355 | -1.23596 |
| C | -0.38584 | 3.122223 | -0.7179 | H | 0.156102 | 3.908076 | 1.20658 |
| C | 0.457576 | 3.074056 | 0.562697 | H | 0.235645 | 2.164293 | 1.127978 |
| C | 1.971306 | 3.166726 | 0.313169 | H | 2.161943 | 4.01533 | -0.35334 |
| C | 2.626986 | 1.910141 | -0.28776 | H | 2.476406 | 3.404035 | 1.25605 |
| C | 2.780153 | 0.762177 | 0.720395 | H | 2.054345 | 1.581035 | -1.16001 |
| C | 3.448012 | -0.53499 | 0.205889 | H | 3.614686 | 2.190226 | -0.66341 |
| C | 2.539655 | -1.28215 | -0.80066 | H | 3.394919 | 1.110729 | 1.55626 |
| O | 3.689292 | -1.3985 | 1.337277 | H | 1.805649 | 0.502042 | 1.140888 |
| C | 4.824294 | -0.24775 | -0.38886 | H | 2.832556 | -1.74563 | 1.620683 |
| O | 1.377277 | -1.70898 | -0.05165 | H | 4.74792 | 0.270562 | -1.34558 |
| C | 3.17648 | -2.48882 | -1.47803 | H | 5.397895 | 0.381395 | 0.294737 |
| C | -4.1864 | -2.02095 | -0.1727 | H | 5.37642 | -1.17451 | -0.54233 |
| C | -3.17923 | -2.02775 | 0.980205 | H | 0.745067 | -2.15872 | -0.63599 |
| C | -1.73612 | -2.00757 | 0.538517 | H | 2.428998 | -3.01065 | -2.08051 |
| O | -0.93059 | -1.40633 | 1.423676 | H | 3.981373 | -2.18522 | -2.14854 |
| O | -1.32001 | -2.49478 | -0.49755 | H | 3.575601 | -3.18705 | -0.74085 |
| H | 2.218294 | -0.56823 | -1.56476 | H | -5.18306 | -2.17672 | 0.249776 |
| H | -4.31097 | 2.299432 | 0.744956 | H | -3.98718 | -2.86894 | -0.83164 |
| H | -5.66549 | 0.548891 | -0.00653 | H | -3.34617 | -1.19235 | 1.661031 |
| H | -1.87763 | 1.465239 | 1.009644 | H | -3.30217 | -2.94055 | 1.574078 |
| H | -1.94803 | 0.861691 | -0.63001 | H | -0.01318 | -1.40106 | 1.062986 |
| H | -4.87672 | -0.87078 | -1.8463 |  |  |  |  |

**Table S7:** (continued)

| **Conformer 9** | | | | | | | |
| --- | --- | --- | --- | --- | --- | --- | --- |
| Atom | X | Y | Z | Atom | X | Y | Z |
| C | 4.352595 | 1.231711 | -0.05967 | H | 3.44786 | -0.8664 | 1.432404 |
| C | 4.792217 | -0.02833 | -0.02882 | H | 2.510948 | 3.111702 | -0.85054 |
| C | 3.24669 | 1.850549 | 0.747683 | H | 1.683857 | 1.573021 | -0.70711 |
| C | 4.307467 | -1.16422 | 0.830037 | H | 0.609572 | 2.365693 | 1.428715 |
| C | 2.103645 | 2.398217 | -0.12419 | H | 1.455304 | 3.887952 | 1.283983 |
| C | 1.005176 | 3.078936 | 0.697541 | H | -0.82791 | 4.195873 | 0.544498 |
| C | -0.14594 | 3.668208 | -0.13219 | H | 0.261053 | 4.430395 | -0.80651 |
| C | -0.95707 | 2.664117 | -0.9651 | H | -1.69993 | 3.221091 | -1.54741 |
| C | -1.67501 | 1.58763 | -0.14359 | H | -0.30585 | 2.178548 | -1.69968 |
| C | -2.49517 | 0.643616 | -1.02918 | H | -0.94669 | 1.002882 | 0.422602 |
| C | -3.07154 | -0.61288 | -0.36007 | H | -2.32053 | 2.07807 | 0.592809 |
| C | -3.93413 | -0.29636 | 0.881507 | H | -3.31737 | 1.192891 | -1.49606 |
| O | -1.93631 | -1.38 | 0.127153 | H | -1.86404 | 0.295004 | -1.85368 |
| C | -3.82303 | -1.46016 | -1.39109 | H | -2.2876 | -2.02808 | 0.756539 |
| O | -4.21688 | -1.57537 | 1.493011 | H | -4.64506 | -0.90142 | -1.84154 |
| C | -5.2211 | 0.469449 | 0.608752 | H | -3.13598 | -1.75299 | -2.18674 |
| C | 3.961965 | -2.43175 | 0.025058 | H | -4.22993 | -2.36327 | -0.93453 |
| C | 2.769329 | -2.27716 | -0.91774 | H | -4.41477 | -1.43304 | 2.425584 |
| C | 1.427953 | -2.17099 | -0.22651 | H | -5.74211 | 0.65763 | 1.549959 |
| O | 0.477955 | -1.70055 | -1.04159 | H | -5.01683 | 1.436359 | 0.144924 |
| O | 1.209074 | -2.49435 | 0.924316 | H | -5.88911 | -0.09634 | -0.042 |
| H | -3.30853 | 0.276583 | 1.570678 | H | 4.832046 | -2.72342 | -0.57078 |
| H | 4.841987 | 1.911668 | -0.75543 | H | 3.767909 | -3.25246 | 0.718938 |
| H | 5.61154 | -0.28586 | -0.69892 | H | 2.886276 | -1.41048 | -1.57218 |
| H | 2.840649 | 1.137035 | 1.468371 | H | 2.698476 | -3.14544 | -1.58242 |
| H | 3.661642 | 2.679511 | 1.335579 | H | -0.3938 | -1.64391 | -0.56586 |
| H | 5.101909 | -1.43018 | 1.538046 |  |  |  |  |

**Table S7:** (continued)

| **Conformer 10** | | | | | | | |
| --- | --- | --- | --- | --- | --- | --- | --- |
| Atom | X | Y | Z | Atom | X | Y | Z |
| C | 2.594689 | 0.212935 | 1.66524 | H | 4.027646 | -2.50405 | 0.61414 |
| C | 2.610806 | -1.03364 | 1.188566 | H | 1.804724 | 2.741432 | 1.398422 |
| C | 3.474439 | 1.380094 | 1.312051 | H | 3.278501 | 3.478114 | 0.806462 |
| C | 3.539776 | -1.62864 | 0.167285 | H | 3.093568 | 2.428202 | -1.35146 |
| C | 2.66937 | 2.570098 | 0.748847 | H | 1.838754 | 1.339545 | -0.81651 |
| C | 2.215401 | 2.359499 | -0.69994 | H | 1.523237 | 4.377156 | -1.05081 |
| C | 1.155587 | 3.35473 | -1.19608 | H | 1.039672 | 3.22526 | -2.27815 |
| C | -0.23157 | 3.227903 | -0.54384 | H | -0.17523 | 3.483389 | 0.519877 |
| C | -0.86212 | 1.839077 | -0.69584 | H | -0.89318 | 3.975165 | -0.99635 |
| C | -2.31782 | 1.78802 | -0.22143 | H | -0.81535 | 1.542244 | -1.74859 |
| C | -2.97558 | 0.391028 | -0.20202 | H | -0.26139 | 1.111135 | -0.14655 |
| C | -2.40768 | -0.48834 | 0.950924 | H | -2.39308 | 2.217411 | 0.782946 |
| O | -2.72099 | -0.31637 | -1.43857 | H | -2.9224 | 2.429453 | -0.87054 |
| C | -4.49543 | 0.527141 | -0.13284 | H | -1.77988 | -0.56892 | -1.47801 |
| O | -2.68141 | -1.88725 | 0.664759 | H | -4.79574 | 1.162281 | 0.701037 |
| C | -2.92445 | -0.17955 | 2.345604 | H | -4.85534 | 0.984248 | -1.0559 |
| C | 2.865394 | -2.08118 | -1.14448 | H | -4.98133 | -0.44409 | -0.01994 |
| C | 1.731109 | -3.10743 | -0.97303 | H | -3.00152 | -1.90711 | -0.25416 |
| C | 0.376601 | -2.49583 | -0.69792 | H | -2.42946 | -0.82883 | 3.070149 |
| O | -0.29333 | -3.09436 | 0.285062 | H | -2.70948 | 0.853671 | 2.623565 |
| O | -0.07639 | -1.56212 | -1.34036 | H | -3.99972 | -0.34601 | 2.417265 |
| H | -1.32001 | -0.37407 | 0.947276 | H | 2.478649 | -1.21455 | -1.68534 |
| H | 1.837602 | 0.442091 | 2.414053 | H | 3.635138 | -2.52868 | -1.77762 |
| H | 1.873 | -1.721 | 1.596912 | H | 1.608559 | -3.66049 | -1.91002 |
| H | 3.987672 | 1.709913 | 2.223119 | H | 1.967286 | -3.84037 | -0.20065 |
| H | 4.254576 | 1.094499 | 0.601943 | H | -1.18743 | -2.66409 | 0.414651 |
| H | 4.337766 | -0.92771 | -0.08502 |  |  |  |  |

**Table S7:** (continued)

| **Conformer 11** | | | | | | | |
| --- | --- | --- | --- | --- | --- | --- | --- |
| Atom | X | Y | Z | Atom | X | Y | Z |
| C | -4.70233 | 0.748631 | -0.19997 | H | -4.15028 | -2.01111 | 1.214958 |
| C | -4.67756 | -0.58243 | -0.29323 | H | -2.08859 | 1.488805 | -0.74597 |
| C | -3.76644 | 1.614301 | 0.599845 | H | -3.1106 | 2.884022 | -1.03223 |
| C | -3.68764 | -1.51261 | 0.353338 | H | -2.32259 | 3.919158 | 1.101976 |
| C | -2.65843 | 2.274692 | -0.241 | H | -1.31082 | 2.530162 | 1.418563 |
| C | -1.72679 | 3.141799 | 0.610334 | H | -1.01641 | 4.480143 | -0.92411 |
| C | -0.5865 | 3.823113 | -0.15937 | H | -0.04777 | 4.478581 | 0.534553 |
| C | 0.430675 | 2.889484 | -0.83361 | H | -0.0529 | 2.32007 | -1.63464 |
| C | 1.13687 | 1.914962 | 0.117511 | H | 1.185242 | 3.513652 | -1.32584 |
| C | 2.325415 | 1.21957 | -0.55645 | H | 1.483883 | 2.462524 | 0.999625 |
| C | 2.951062 | 0.043465 | 0.210201 | H | 0.427078 | 1.164961 | 0.47669 |
| C | 4.17242 | -0.52165 | -0.57018 | H | 2.015648 | 0.84746 | -1.54122 |
| O | 1.993515 | -1.05515 | 0.28086 | H | 3.102836 | 1.963612 | -0.74965 |
| C | 3.29337 | 0.379228 | 1.657328 | H | 1.532228 | -1.16831 | -0.56877 |
| O | 4.570467 | -1.78202 | -0.01857 | H | 3.933139 | 1.260876 | 1.710453 |
| C | 5.409226 | 0.361596 | -0.59557 | H | 2.384409 | 0.583465 | 2.222756 |
| C | -3.18335 | -2.59576 | -0.61441 | H | 3.813776 | -0.45435 | 2.130317 |
| C | -2.00855 | -3.41039 | -0.04565 | H | 3.752916 | -2.27102 | 0.154182 |
| C | -0.72731 | -2.61222 | -0.04132 | H | 6.187415 | -0.125 | -1.18637 |
| O | -0.20385 | -2.42349 | 1.175209 | H | 5.201411 | 1.330393 | -1.05033 |
| O | -0.21124 | -2.17002 | -1.05342 | H | 5.798935 | 0.522577 | 0.41089 |
| H | 3.832704 | -0.6837 | -1.60318 | H | -4.0007 | -3.2816 | -0.85116 |
| H | -5.46951 | 1.278639 | -0.76054 | H | -2.87188 | -2.1406 | -1.55794 |
| H | -5.43573 | -1.06382 | -0.90842 | H | -2.22905 | -3.76589 | 0.961308 |
| H | -4.34943 | 2.402059 | 1.09056 | H | -1.82891 | -4.28185 | -0.68046 |
| H | -3.30063 | 1.036416 | 1.402048 | H | 0.619339 | -1.88345 | 1.084845 |
| H | -2.84119 | -0.94618 | 0.747534 |  |  |  |  |

**Table S7:** (continued)

| **Conformer 12** | | | | | | | |
| --- | --- | --- | --- | --- | --- | --- | --- |
| Atom | X | Y | Z | Atom | X | Y | Z |
| C | 4.371927 | 0.748782 | -0.47418 | H | 2.850884 | -0.46116 | 1.429865 |
| C | 4.535331 | -0.44941 | 0.08992 | H | 2.537544 | 1.74858 | -2.13306 |
| C | 3.348624 | 1.79933 | -0.13258 | H | 1.574525 | 0.890663 | -0.95655 |
| C | 3.732528 | -1.06405 | 1.204452 | H | 1.813197 | 3.918255 | -1.32709 |
| C | 2.155614 | 1.8052 | -1.10796 | H | 0.433597 | 2.925228 | -1.73201 |
| C | 1.245248 | 3.037609 | -1.00561 | H | 1.461461 | 3.613938 | 1.066183 |
| C | 0.653014 | 3.340269 | 0.380245 | H | 0.025714 | 4.234649 | 0.289748 |
| C | -0.17655 | 2.225858 | 1.033468 | H | 0.459798 | 1.361267 | 1.250471 |
| C | -1.39041 | 1.755263 | 0.222394 | H | -0.52153 | 2.592278 | 2.007279 |
| C | -2.31221 | 0.869696 | 1.072792 | H | -1.94244 | 2.632349 | -0.13302 |
| C | -3.52505 | 0.185016 | 0.381081 | H | -1.05402 | 1.220279 | -0.66593 |
| C | -3.13619 | -1.18378 | -0.22728 | H | -1.71707 | 0.085441 | 1.550154 |
| O | -4.0014 | 0.952234 | -0.74845 | H | -2.69835 | 1.48907 | 1.889098 |
| C | -4.65395 | 0.021001 | 1.403636 | H | -4.09317 | 1.875697 | -0.48604 |
| O | -2.14048 | -0.99803 | -1.25599 | H | -4.34167 | -0.62334 | 2.229832 |
| C | -4.29189 | -1.99967 | -0.79764 | H | -4.91486 | 0.995696 | 1.821765 |
| C | 3.310928 | -2.51689 | 0.911235 | H | -5.54751 | -0.4039 | 0.948187 |
| C | 2.404564 | -2.68441 | -0.30932 | H | -2.43127 | -0.25997 | -1.81071 |
| C | 0.997901 | -2.15758 | -0.13053 | H | -3.88948 | -2.89019 | -1.28298 |
| O | 0.420409 | -1.85528 | -1.29849 | H | -4.9781 | -2.32559 | -0.0153 |
| O | 0.428369 | -2.04563 | 0.936812 | H | -4.85375 | -1.42555 | -1.53668 |
| H | -2.63321 | -1.76089 | 0.550055 | H | 4.209384 | -3.1199 | 0.749424 |
| H | 5.041887 | 1.009275 | -1.29143 | H | 2.81144 | -2.92846 | 1.790826 |
| H | 5.338637 | -1.07721 | -0.29353 | H | 2.838892 | -2.2215 | -1.19703 |
| H | 2.990004 | 1.670665 | 0.890491 | H | 2.29477 | -3.74904 | -0.54594 |
| H | 3.836104 | 2.780787 | -0.16704 | H | -0.51227 | -1.5401 | -1.16332 |
| H | 4.339057 | -1.07716 | 2.118482 |  |  |  |  |

**Table S7:** (continued)

| **Conformer 13** | | | | | | | |
| --- | --- | --- | --- | --- | --- | --- | --- |
| Atom | X | Y | Z | Atom | X | Y | Z |
| C | 4.534836 | 0.712868 | 0.58427 | H | 4.530068 | -1.34543 | -1.79074 |
| C | 4.570758 | -0.56279 | 0.193664 | H | 1.788446 | 1.135295 | 0.481939 |
| C | 3.74898 | 1.82997 | -0.05077 | H | 2.488882 | 2.212889 | 1.672968 |
| C | 3.830447 | -1.18459 | -0.96072 | H | 2.108021 | 4.167913 | 0.284565 |
| C | 2.369952 | 2.053681 | 0.595175 | H | 1.685248 | 3.192489 | -1.10712 |
| C | 1.61082 | 3.238608 | -0.01361 | H | 0.030391 | 3.192642 | 1.464112 |
| C | 0.127047 | 3.302004 | 0.376706 | H | -0.26583 | 4.295996 | 0.135374 |
| C | -0.738 | 2.249169 | -0.32637 | H | -0.6669 | 2.400273 | -1.41007 |
| C | -2.20877 | 2.304655 | 0.093326 | H | -0.33418 | 1.254553 | -0.13181 |
| C | -3.18274 | 1.436925 | -0.72155 | H | -2.29251 | 2.076386 | 1.160734 |
| C | -3.13159 | -0.10016 | -0.61562 | H | -2.55527 | 3.339359 | -0.0097 |
| C | -3.04438 | -0.61679 | 0.843808 | H | -4.1991 | 1.735489 | -0.45126 |
| O | -1.91575 | -0.55723 | -1.3036 | H | -3.07193 | 1.6799 | -1.78316 |
| C | -4.3239 | -0.69409 | -1.36647 | H | -2.13276 | -1.3906 | -1.74072 |
| O | -2.91668 | -2.04057 | 0.866 | H | -5.26832 | -0.36355 | -0.93406 |
| C | -4.23973 | -0.24723 | 1.713056 | H | -4.30214 | -0.37417 | -2.41039 |
| C | 3.221276 | -2.55587 | -0.61353 | H | -4.29965 | -1.78513 | -1.32883 |
| C | 2.20483 | -2.57637 | 0.541539 | H | -1.97373 | -2.26635 | 0.763413 |
| C | 0.783592 | -2.17659 | 0.232711 | H | -4.0578 | -0.57859 | 2.73657 |
| O | 0.656979 | -1.1973 | -0.65682 | H | -4.41259 | 0.829431 | 1.730685 |
| O | -0.17644 | -2.6968 | 0.782513 | H | -5.14804 | -0.73695 | 1.356907 |
| H | -2.14179 | -0.17985 | 1.283597 | H | 2.768325 | -2.98681 | -1.51013 |
| H | 5.122254 | 0.987087 | 1.457952 | H | 4.035345 | -3.22957 | -0.33016 |
| H | 5.200718 | -1.2463 | 0.761363 | H | 2.1465 | -3.57282 | 0.979209 |
| H | 4.330561 | 2.755776 | 0.020372 | H | 2.541713 | -1.90837 | 1.343444 |
| H | 3.606216 | 1.638833 | -1.11779 | H | -0.30156 | -0.96053 | -0.78331 |
| H | 3.055836 | -0.51465 | -1.33253 |  |  |  |  |

**Table S7:** (continued)

| **Conformer 14** | | | | | | | |
| --- | --- | --- | --- | --- | --- | --- | --- |
| Atom | X | Y | Z | Atom | X | Y | Z |
| C | 4.64683 | 0.753412 | -0.20129 | H | 3.972102 | -2.19004 | -1.25115 |
| C | 4.592602 | -0.56547 | -0.00338 | H | 3.354909 | 3.144678 | 0.485622 |
| C | 3.700589 | 1.602978 | -1.00651 | H | 2.218113 | 3.145778 | -0.84857 |
| C | 3.539973 | -1.51876 | -0.49784 | H | 1.288363 | 0.933659 | 0.063133 |
| C | 2.761167 | 2.489031 | -0.16235 | H | 2.327866 | 1.132123 | 1.450033 |
| C | 1.771323 | 1.689464 | 0.690979 | H | 0.18162 | 1.904845 | 2.12302 |
| C | 0.697376 | 2.534177 | 1.390897 | H | 1.186083 | 3.324591 | 1.972355 |
| C | -0.34185 | 3.188705 | 0.46444 | H | -1.03455 | 3.770053 | 1.083929 |
| C | -1.1588 | 2.234317 | -0.42336 | H | 0.156068 | 3.915327 | -0.18522 |
| C | -2.0103 | 1.234916 | 0.369067 | H | -1.80647 | 2.846978 | -1.05589 |
| C | -2.9203 | 0.32338 | -0.46634 | H | -0.49598 | 1.693741 | -1.10355 |
| C | -3.60862 | -0.72754 | 0.438442 | H | -1.36357 | 0.594156 | 0.974719 |
| O | -2.05966 | -0.43182 | -1.36962 | H | -2.64151 | 1.790501 | 1.068322 |
| C | -3.93047 | 1.104801 | -1.31071 | H | -2.62098 | -1.14563 | -1.71279 |
| O | -4.23533 | -1.66963 | -0.46335 | H | -4.52167 | 1.78247 | -0.69222 |
| C | -4.62935 | -0.18776 | 1.431069 | H | -3.41179 | 1.696249 | -2.06432 |
| C | 2.96844 | -2.38432 | 0.638573 | H | -4.61329 | 0.424557 | -1.82183 |
| C | 1.751424 | -3.24198 | 0.224889 | H | -4.30423 | -2.52132 | -0.01764 |
| C | 0.473845 | -2.43582 | 0.190076 | H | -5.04258 | -1.01393 | 2.013829 |
| O | 0.239407 | -1.85231 | -0.9899 | H | -4.17325 | 0.513543 | 2.131921 |
| O | -0.26399 | -2.30269 | 1.148002 | H | -5.45396 | 0.31242 | 0.920895 |
| H | -2.80779 | -1.24439 | 0.974983 | H | 3.749142 | -3.05738 | 1.002818 |
| H | 5.466579 | 1.295813 | 0.266728 | H | 2.689303 | -1.75145 | 1.485936 |
| H | 5.378262 | -1.01724 | 0.600295 | H | 1.929178 | -3.69875 | -0.75079 |
| H | 4.295566 | 2.255233 | -1.65554 | H | 1.602648 | -4.03567 | 0.957053 |
| H | 3.093525 | 0.982577 | -1.67025 | H | -0.59745 | -1.31463 | -0.95483 |
| H | 2.733036 | -0.97852 | -0.99207 |  |  |  |  |

**Table S7:** (continued)

| **Conformer 15** | | | | | | | |
| --- | --- | --- | --- | --- | --- | --- | --- |
| Atom | X | Y | Z | Atom | X | Y | Z |
| C | 4.516312 | -0.40432 | 0.154292 | H | 3.436746 | 2.310471 | 1.102596 |
| C | 4.23491 | 0.841331 | -0.23196 | H | 4.494398 | -3.12005 | 0.3867 |
| C | 3.801106 | -1.24176 | 1.178021 | H | 3.229322 | -3.28102 | 1.587165 |
| C | 3.103975 | 1.717251 | 0.240465 | H | 2.774088 | -2.21965 | -1.24797 |
| C | 3.543619 | -2.68977 | 0.720184 | H | 2.543183 | -3.8857 | -0.75823 |
| C | 2.502474 | -2.85254 | -0.39522 | H | 0.819124 | -3.14077 | 0.919768 |
| C | 1.064475 | -2.54169 | 0.033792 | H | 0.990385 | -1.49519 | 0.342367 |
| C | 0.035712 | -2.83553 | -1.06299 | H | 0.270017 | -2.24175 | -1.95464 |
| C | -1.42664 | -2.58349 | -0.66041 | H | 0.136682 | -3.8846 | -1.36152 |
| C | -1.76916 | -1.09526 | -0.51792 | H | -2.07455 | -3.04201 | -1.41156 |
| C | -3.19853 | -0.7598 | -0.04117 | H | -1.61706 | -3.11896 | 0.278698 |
| C | -3.32652 | 0.774198 | 0.152086 | H | -1.0692 | -0.62402 | 0.173587 |
| O | -3.42897 | -1.29065 | 1.28833 | H | -1.6227 | -0.61027 | -1.48832 |
| C | -4.26035 | -1.29437 | -1.00333 | H | -3.14637 | -2.21222 | 1.313873 |
| O | -2.45789 | 1.187223 | 1.231741 | H | -4.17765 | -0.814 | -1.98162 |
| C | -4.73582 | 1.285292 | 0.427848 | H | -4.1425 | -2.36915 | -1.14693 |
| C | 2.623387 | 2.685652 | -0.84879 | H | -5.26177 | -1.1186 | -0.61283 |
| C | 1.477002 | 3.614885 | -0.4026 | H | -2.51329 | 0.497475 | 1.911657 |
| C | 0.162135 | 2.880558 | -0.28288 | H | -4.68839 | 2.352441 | 0.650112 |
| O | -0.16675 | 2.568368 | 0.973107 | H | -5.38531 | 1.152083 | -0.43818 |
| O | -0.52769 | 2.581308 | -1.23969 | H | -5.18383 | 0.773566 | 1.281913 |
| H | -2.92689 | 1.262302 | -0.73806 | H | 3.462452 | 3.314849 | -1.15885 |
| H | 5.389629 | -0.87885 | -0.29108 | H | 2.309765 | 2.128013 | -1.73618 |
| H | 4.902453 | 1.301784 | -0.95831 | H | 1.725911 | 4.089942 | 0.54763 |
| H | 4.428399 | -1.28427 | 2.077823 | H | 1.337003 | 4.394801 | -1.15277 |
| H | 2.86825 | -0.76694 | 1.489512 | H | -1.01794 | 2.048333 | 0.988915 |
| H | 2.271488 | 1.10803 | 0.600259 |  |  |  |  |

**Table S7:** (continued)

| **Conformer 16** | | | | | | | |
| --- | --- | --- | --- | --- | --- | --- | --- |
| Atom | X | Y | Z | Atom | X | Y | Z |
| C | 4.670855 | -0.77427 | 0.151694 | H | 3.810058 | 1.949674 | 1.335405 |
| C | 4.496998 | 0.523206 | -0.10738 | H | 4.204275 | -3.5089 | 0.051235 |
| C | 3.877238 | -1.64311 | 1.086806 | H | 2.918144 | -3.56243 | 1.2401 |
| C | 3.431197 | 1.434659 | 0.442777 | H | 2.764487 | -2.22493 | -1.50917 |
| C | 3.353159 | -2.94385 | 0.44735 | H | 2.045702 | -3.75431 | -1.04848 |
| C | 2.314196 | -2.76239 | -0.66706 | H | 0.67299 | -2.47931 | 0.698856 |
| C | 1.03852 | -2.03321 | -0.23241 | H | 1.27344 | -0.98981 | 0.000593 |
| C | -0.06574 | -2.06376 | -1.29579 | H | 0.377044 | -1.82963 | -2.27043 |
| C | -1.2208 | -1.0798 | -1.05322 | H | -0.4599 | -3.08377 | -1.3796 |
| C | -1.98352 | -1.33051 | 0.252606 | H | -0.82508 | -0.06046 | -1.05956 |
| C | -3.06627 | -0.31143 | 0.638612 | H | -1.90644 | -1.14723 | -1.90313 |
| C | -4.07677 | -0.03467 | -0.49636 | H | -2.44534 | -2.32186 | 0.220771 |
| O | -2.39199 | 0.952919 | 0.898813 | H | -1.28114 | -1.35419 | 1.090177 |
| C | -3.75504 | -0.74945 | 1.93511 | H | -3.08204 | 1.633564 | 0.858231 |
| O | -4.88533 | 1.072317 | -0.03569 | H | -4.23596 | -1.7225 | 1.822677 |
| C | -4.95189 | -1.21411 | -0.89688 | H | -3.01032 | -0.82807 | 2.728824 |
| C | 2.996464 | 2.500661 | -0.57324 | H | -4.51097 | -0.02479 | 2.240173 |
| C | 1.82717 | 3.385105 | -0.09118 | H | -5.25255 | 1.524492 | -0.80373 |
| C | 0.495005 | 2.682559 | -0.213 | H | -5.60877 | -0.92183 | -1.71897 |
| O | 0.065373 | 2.138211 | 0.930802 | H | -4.34888 | -2.0576 | -1.23872 |
| O | -0.12407 | 2.598267 | -1.25617 | H | -5.57541 | -1.54529 | -0.06542 |
| H | -3.50429 | 0.30527 | -1.36271 | H | 3.847207 | 3.152553 | -0.78952 |
| H | 5.500906 | -1.27574 | -0.34396 | H | 2.721821 | 2.028318 | -1.521 |
| H | 5.203623 | 0.997292 | -0.78658 | H | 1.99196 | 3.699468 | 0.94071 |
| H | 4.531193 | -1.92677 | 1.921209 | H | 1.768858 | 4.27557 | -0.71849 |
| H | 3.052686 | -1.08402 | 1.532778 | H | -0.80007 | 1.672429 | 0.790108 |
| H | 2.566236 | 0.857343 | 0.774995 |  |  |  |  |
